# LINC01235 Promotes Clonal Evolution through DNA Replication Licensing‐Induced Chromosomal Instability in Breast Cancer

**DOI:** 10.1002/advs.202413527

**Published:** 2025-02-14

**Authors:** Qi Zhang, Xuliren Wang, Zhibo Shao, Yi Zhang, Liyi Zhang, Ming Chen, Xujie Zhou, Han Zhu, Yue Zhou, Xinya Lu, Pei Li, Weiru Chi, Lun Li, Zhi‐Ming Shao, Shenglin Huang, Jingyan Xue, Yayun Chi, Jiong Wu, Bingqiu Xiu

**Affiliations:** ^1^ Department of Breast Surgery Key Laboratory of Breast Cancer in Shanghai Fudan University Shanghai Cancer Center Shanghai 200032 China; ^2^ Department of Oncology Fudan University Shanghai Medical College Shanghai 200032 China; ^3^ Department of General Surgery The Second Xiangya Hospital Central South University Hunan 410011 China; ^4^ Fudan University Shanghai Cancer Center Key Laboratory of Medical Epigenetics and Metabolism Institutes of Biomedical Sciences Fudan University Shanghai 200032 China; ^5^ Pathology Center Shanghai General Hospital Shanghai Jiaotong University School of Medicine Shanghai 200080 China; ^6^ Collaborative Innovation Center for Cancer Medicine Shanghai Medical College Fudan University Shanghai 200032 China

**Keywords:** ATR inhibitors, chromosomal instabilities, DNA replication licensing, HER2‐positive breast cancers, LINC01235

## Abstract

Despite the development of HER2‐targeting drugs such as trastuzumab and T‐DXd, treatment resistance is a substantial challenge, often leading to relapse and distant metastasis. Tumor heterogeneity in HER2‐positive breast cancer drives the evolution of resistant clones following therapeutic stress. However, the targetable drivers of anti‐HER2 treatment resistance are not thoroughly identified. This study aims to use neoadjuvant‐targeted therapy cohorts and a patient‐derived organoid in vitro treatment model to uncover the potential targetable drivers of anti‐HER2 treatment resistance. it is found that LINC01235 significantly enhances DNA replication licensing and chromosomal instability, fostering clonal expansion and evolution, and ultimately increasing resistance to therapeutic interventions. LINC01235 regulates global H3K27ac, H3K9ac, and H3K36me3 modifications, promotes H2A.Z expression in regulatory regions, and increases the accessibility of DNA licensing factors to their promoter regions. XRCC5 is identified as a key component for maintaining genomic stability, crucial for LINC01235's role in replication licensing. Furthermore, therapeutic strategies targeting LINC01235, including the use of antisense oligonucleotides or ATR inhibitors, which showed promise in overcoming treatment resistance are explored. These findings underscore the pivotal role of LINC01235 in driving resistance mechanisms and highlight novel avenues for targeted therapies to improve the outcomes of patients with HER2‐positive breast cancer.

## Graphical abstract

This study elucidates the mechanism underlying LINC01235 promotes clonal evolution and selection under treatment pressure by facilitating DNA replication licensing and CIN, leading to increased therapeutic resistance in HER2+ breast cancer. Targeting LINC01235‐related pathways with ATR inhibitors may be a viable strategy for clinical application.

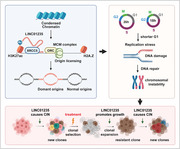

## 1 Introduction

The primary therapeutic approach for early‐stage HER2‐positive breast cancer involves HER2‐targeted therapy combined with chemotherapy.^[^ ^1^ ^]^ HER2‐targeting drugs include monoclonal antibodies (mAbs), such as trastuzumab and pertuzumab; small molecule tyrosine kinase inhibitors (TKIs), such as lapatinib and neratinib; and antibody‐drug conjugates (ADCs), such as ado‐trastuzumab emtansine (T‐DM1) and trastuzumab deruxtecan (T‐DXd).^[^ ^1^, ^2^ ^]^ The rapid evolution of HER2‐targeted therapeutics has significantly improved patient prognoses. However, ≈15–31% of early‐stage patients remain susceptible to relapse or distant metastasis.^[^ ^2a^ ^]^ Moreover, 45–62% of patients with early‐stage breast cancer exhibit intrinsic resistance to targeted therapies, increasing their risk of tumor recurrence and metastasis, thus impacting mortality rates. In late‐stage HER2+ breast cancer, despite favorable responses to T‐DXd treatment, a subset of patients develops secondary resistance to targeted therapies within a year of treatment initiation.^[^ ^3^ ^]^ Consequently, there is an enduring unmet therapeutic need for this subset of patients with HER2‐positive breast cancer, necessitating further elucidation of resistance mechanisms, identification of novel therapeutic targets, and development of innovative therapeutics to enhance patient survival.

Neoadjuvant therapy prior to surgery for locally advanced breast cancer is crucial for investigating drug resistance.^[^ ^4^ ^]^ Biomarker analysis of samples collected before and after neoadjuvant treatment can facilitate the prediction of treatment efficacy and prognosis. Trials such as CREATE‐X and KATHERINE have demonstrated that patients who do not achieve a pathological complete response (pCR) could benefit from additional intensive treatment.^[^ ^5^ ^]^ Therefore, leveraging neoadjuvant chemotherapy clinical cohorts and biological specimen analysis could provide a great opportunity for exploring treatment resistance mechanisms in HER2‐positive breast cancer.

Treatment resistance in cancer commonly emerges under different treatment pressures.^[^ ^6^ ^]^ Cancer cells undergo a cyclical process of clonal expansion, genetic diversification, and clonal selection in response to various stressors, including chemotherapy, radiation therapy, and targeted therapy.^[^ ^7^ ^]^ Within this dynamic framework, certain resistant clones may arise, either intrinsically or through acquired mechanisms.^[^ ^6c^ ^]^ The intrinsic heterogeneity of breast cancer is characterized by a plethora of genetic mutations and subsequent cellular subclones.^[^ ^7^, ^8^ ^]^ After treatment, the waning of sensitive subclones facilitates the expansion of treatment‐resistant cells, thereby establishing a dominant resistant subpopulation.^[^ ^9^ ^]^ The identification and comprehension of pivotal genes orchestrating resistance, coupled with an understanding of the intricate mechanisms underlying the development of resistance, are imperative for devising effective strategies to mitigate the resistant phenotype in breast cancer.

Among the cancer hallmarks, chromosomal instability (CIN) feeds on the clonal diversity from which resistant clones have evolved.^[^ ^10^ ^]^ In normal cells, fidelity of DNA replication is essential for maintaining genomic integrity.^[^ ^11^ ^]^ Eukaryotic cells harbor a complex network of replication origins that are synchronously activated during the S phase of the cell cycle.^[^ ^12^ ^]^ This activation is orchestrated by the origin recognition complex (ORC), facilitating the assembly of crucial proteins such as minichromosome maintenance protein (MCM)2‐7, cell division cycle 6 (CDC6), and chromatin licensing and DNA replication factor 1 (CDT1) onto the DNA, forming a pre‐replication complex—a process known as “replication licensing”.^[^ ^13^ ^]^ A recent study revealed that unscheduled replication due to over‐licensing in the G1 phase markedly intensifies CIN during subsequent S‐phase replication.^[^ ^14^ ^]^ In addition, oncogenes such as c‐Myc and Cyclin E can cause CINs by promoting DNA licensing, fueling the oncological transformation of normal cells.^[^ ^15^ ^]^ Therefore, dysregulation of this replication machinery has been implicated in tumorigenesis,^[^ ^16^ ^]^ where excessive licensing leading to over‐replication results in the accumulation of mutations and chromosomal instability, which diversifies the repertoire of new resistant clones.

However, the targetable drivers of anti‐HER2 treatment resistance have not been sufficiently unraveled. Therefore, this study aims to provide insights into the drivers of resistance in HER2‐positive breast cancer. By analyzing the expression profiles of treatment‐sensitive and‐resistant tumors using clinical samples and patient‐derived organoids before and after treatment, we identified several genes that are either proven or newly identified to be involved in treatment resistance. Our study identified LINC01235 as a pivotal regulator of the incomplete response of HER2+ tumors to therapy. We elucidated its role in licensing DNA replication and chromosomal instability, underscored its interactions with key proteins, and explored potential strategies to target this breast‐expressing long non‐coding RNA (lncRNA). These findings will enhance our understanding of the molecular drivers of resistance and offer promising directions for targeted therapeutic approaches for the treatment of breast cancer.

## 2 Results

### 2.1 Exploration of the Genes Involved in Multi‐Drug Resistance in HER2‐Positive Breast Cancer

**Figure 1:**
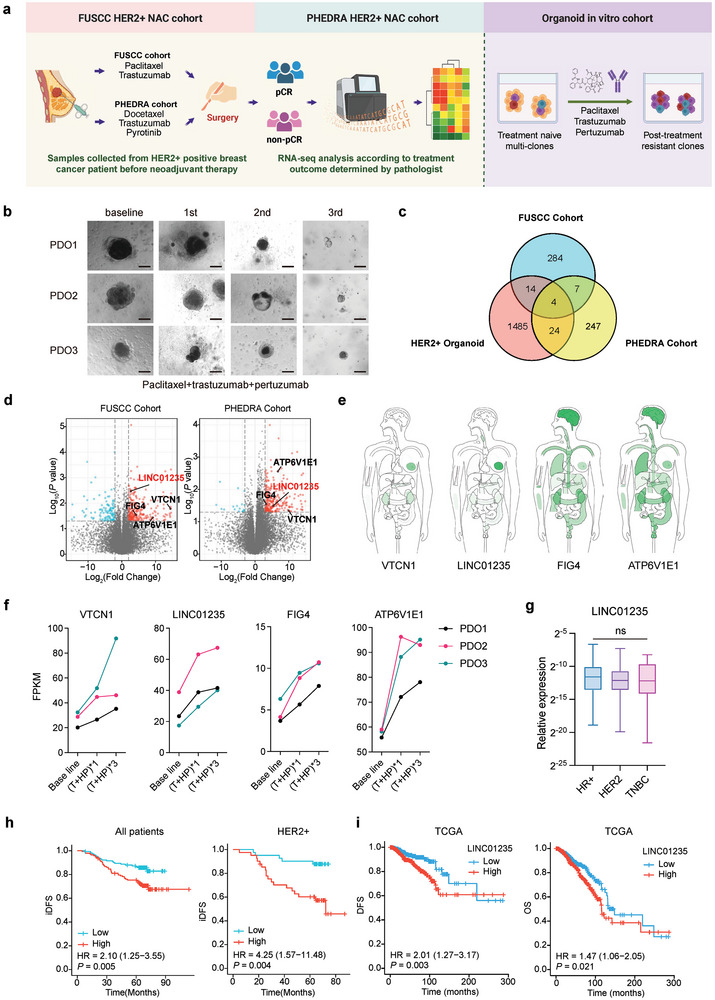
Exploration of genes involved in multi‐drug resistance in HER2‐positive breast cancer. a) The schematic view of the exploration of drug‐resistant candidate drivers through HER2+ breast cancer (BC neoadjuvant cohort. According to the post‐surgical pathological examination results, core needle biopsy specimens from the FUSCC and PHEDRA cohort before neoadjuvant treatment were divided into pCR and non‐pCR groups. Three patient‐derived organoids (PDO) from patients with HER2+ BC were used for in vitro screening. Before and after the indicated treatment, samples were collected and assayed by RNA‐seq. b) Example of the organoids before and after the first, second, and third cycles of treatments. Scale bar = 50 µm. c) The number of upregulated genes in each of the three datasets. d) Volcano plots showing the four potential drivers in the FUSCC and PHEDRA cohorts. e) Relative expression of VTCN1, LINC01235, Figure 4, and ATP6V1E1 in normal tissues using transcript per million (TPM)+1 data from Genotype‐Tissue Expression (GTEx) and plotted by Gene Expression Profiling Interactive Analysis (GEPIA). f) RNA‐seq FPKM value of VTCN1, LINC1235, Figure 4, and ATP6V1E1 of the three organoids before and after the first and third cycles of trastuzumab (H), pertuzumab (P), and paclitaxel (T). g) Relative expression of LINC01235 in different subtypes of BC from the FUSCC early BC cohorts. Box and whisker plots indicated the median, interquartile range, and min to max range. Kruskal–Wallis test was used for analysis. ns, not significant. h) Kaplan–Meier curves for invasive disease‐free survival (iDFS) of LINC01235‐low and ‐high patients from the FUSCC early BC cohorts. Median splits were used as cut‐off values. i) Kaplan–Meier curves for DFS and overall survival (OS) of LINC01235‐low and ‐high patients from TCGA datasets. For h,i), *P*‐values and hazard ratios (HR) were calculated using the log‐rank test. HR, hormone receptor; TNBC, triple‐negative breast cancer. *P‐*values and hazard ratios (HR) were calculated using a log‐rank test (Additional details on Figure S1, Supporting Information).

To investigate the cellular subclones and key driver genes associated with multidrug resistance in HER2+ breast cancer, we analyzed the differentially expressed genes at baseline between samples that achieved a total pathological complete response (tpCR) and those that did not (non‐tpCR). Our analysis included a consecutive cohort of 63 patients from our center, all of whom were treated with trastuzumab and chemotherapy and achieved a tpCR rate of 55.5% (**Figure** **1**a, Table S1, Supporting Information). Additionally, we examined samples from a phase III PHEDRA anti‐HER2 neoadjuvant clinical trial (NCT03588091) in which patients received either pyrotinib or a placebo along with trastuzumab and docetaxel, yielding a tpCR rate of 52% (Table S2, Supporting Information). Given that resistant clones and driver genes may initially be present in a minority and become enriched post‐treatment, we conducted in vitro simulations of anti‐HER2 therapy combined with chemotherapy using three organoids derived from HER2+ patients (Figure 1b). RNA‐seq was performed before and after the first and third treatment cycles with trastuzumab, pertuzumab, and paclitaxel to capture the early and late‐treatment gene expression changes. By integrating the top‐ranking genes identified across the three cohorts, we identified four genes, VTCN1, LINC01235, ATP6V1E1, and Figure 4, as primary candidates for further investigation of their roles in drug resistance (Figure 1c–f).

To identify future targets specific to the breast tissue that could potentially limit toxic and off‐target effects, we assessed the expression levels of these genes in normal tissues by using Gene Expression Profiling Interactive Analysis (GEPIA). Our findings revealed that VTCN1 and LINC01235 were predominantly expressed in breast tissue, whereas Figure 4 and ATP6V1E1 were expressed more broadly across various tissues (Figure 1e). The VTCN1 gene, which encodes the B7‐H4 receptor, was notably overexpressed in the non‐pCR group following treatment and has been widely implicated in epithelial cell transformation, tumorigenesis, and modulation of the tumor microenvironment.^[^ ^17^ ^]^ Due to its therapeutic potential, ADCs targeting B7‐H4 are currently under preclinical evaluation.^[^ ^18^ ^]^ We focused on another target, the long non‐coding RNA LINC01235, to explore new potential therapeutic targets. Recent developments in RNA treatment have potentiated the translational use of these druggable targets.^[^ ^19^ ^]^ To examine the role of LINC01235 in breast cancer, we first analyzed its expression profile and clinical correlations. Among the different subtypes, LINC01235 was not differentially expressed in our early‐stage breast cancer cohort (Figure 1g). Survival analysis showed that higher LINC01235 expression was correlated with poor invasive disease‐free survival (iDFS) in all patients except triple‐negative breast cancer (TNBC), which may be due to few samples and events, while it was most significant in the HER2+ subtype, indicating that its role in treatment resistance is shared among all breast cancers (Figure 1h; Figure S1c, Supporting Information). The TCGA database also supports the correlation between high LINC01235 expression and poor DFS and overall survival (OS) (Figure 1i). Since we mainly focused on the treatment resistance of HER2‐positive breast cancers, we further investigated the role of LINC01235 in this subtype in the following study.

### 2.2 LINC01235 Promotes Treatment Resistance through Clonal Selection

**Figure 2:**
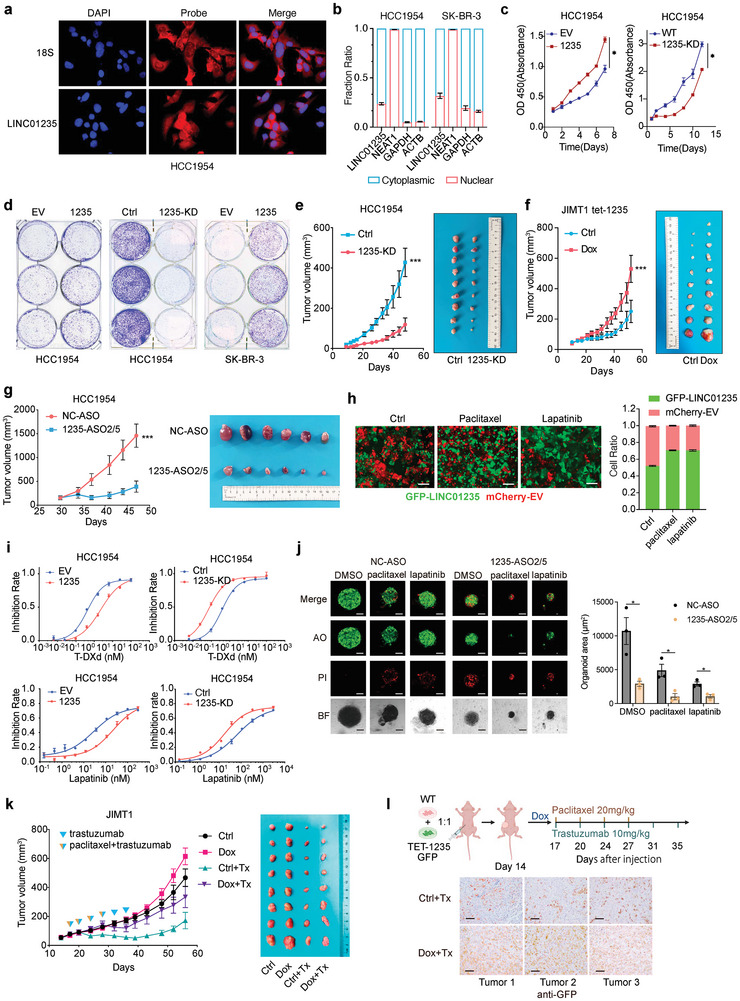
LINC01235 promotes treatment resistance through clonal selection. a) RNA FISH with LINC01235 probe showed LINC01235 located in both cytoplasmic and nuclear fraction. The cell nucleus was stained with DAPI. 18S RNA was probed as the positive control. b) RNA from cytoplasmic and nuclear fractions were extracted. LINC01235, Glyceraldehyde‐3‐phosphate dehydrogenase (GAPDH), and NEAT1 expression were analyzed by RT‐qPCR. *n* = 3 biological replicates. c) Cell proliferation assay of LINC01235 (1235) overexpression compared to empty vector (EV) HCC1954 cells, and LINC01235‐knockdown (1235‐KD) compared to wildtype (WT) cells. *n* = 6 for each time point. d) Clonal formation assay of HCC1954 and SK‐BR‐3 cells. e) Analysis of tumor volume in nude mice subcutaneously injected with HCC1954 control (Ctrl) and LINC01235‐KD cells, *n *= 9 for each group. Left, growth curve. Right, the tumor was dissected at the endpoint. f) Analysis of tumor volume in nude mice subcutaneously injected with JIMT1 dox‐induced 1235 cells. The mice were randomly divided into two groups based on size. Doxycycline (dox) diets were given as indicated. *n* = 9 for control group, *n* = 10 for dox group. Left, growth curve. Right, the tumor was dissected at the endpoint. g) Analysis of tumor volume in nude mice subcutaneously injected with HCC1954 cells. ASO scramble control (NC‐ASO) or targeting LINC01235 (1235‐ASO2/5) were given intravenously through tail vein injection after tumor formation. *n* = 6 for each group. Left, growth curve. Right, the tumor was dissected at the endpoint. h) clonal competition assay between GFP‐LINC01235 and mCherry‐EV JIMT1 cells. The growth was monitored by Cytation5. Paclitaxel (10nM) and lapatinib (200 nm) were given for 3 days. The ratio of GFP and mCherry‐positive cells were analyzed. Scale bar 300 µm. i) Growth inhibition assays of lapatinib and T‐DXd were performed in HCC1954 cells. *n* = 3 for each concentration. j) Human HER2+BC organoids were treated with ASO‐NC or ASO‐1235. Representative images of live (AO) or dead staining (PI) are shown on the left. BF, bright field. As shown, Paclitaxel (70 nm), lapatinib (500 nm), and DMSO control were given. Tumor growth was measured by live organoid area. *n* = 3 for each sample. Statistical analysis was performed using a two‐sided *t‐*test. k) Analysis of tumor volume in nude mice subcutaneously injected with JIMT1 dox‐induced LINC01235 cells. After tumor formation, the mice were randomized into four groups by size. dox diets were given to induce LINC01235 expression. Trastuzumab (10 mg k^−1 ^g) and paclitaxel (20 mg k^−1 ^g) treatments (Tx) were given as indicated. Left, growth curve. Right, tumor dissected at the endpoint. l) Analysis of clonal competition in nude mice subcutaneously injected with JIMT1‐induced LINC01235 cells mixed in a 1:1 ratio with JIMT1 WT cells. After tumor formation, mice were given dox diets and treated as illustrated. Immunohistochemistry was performed targeting GFP. *n* = 3 for each group. Scare bar 100 µm. For (a–g), (i), and (k–l), *n* = 2 experiments. For (c),(e–g), and (k), statistical analysis was performed using a two‐sided *t*‐test at the endpoint. *p < 0.05, **p < 0.01, ***p < 0.001. Error bars represent mean ± SEM (standard error of the mean). (see Figure S2, Supporting Information for more details).

We analyzed the expression levels and splicing isoforms of LINC01235 in breast cancer cells and observed that HCC1954 cells overexpressed LINC01235, whereas SK‐BR‐3 and JIMT1 cells displayed the lowest expression levels (Figure S2a, Supporting Information). Rapid amplification of cDNA ends (RACE) PCR of HCC1954 cells identified four LINC01235 isoforms that differed from those reported in the Ensembl database (Figure S2b, Supporting Information). Northern blot analysis confirmed these findings and identified the full‐length transcript as the most abundant isoform (Figure S2c, Supporting Information). Using the Coding Potential Calculator (CPC),^[^ ^20^ ^]^ Coding‐Potential Assessment Tool (CPAT),^[^ ^21^ ^]^ and PhyloCSF^[^ ^22^ ^]^ tools, we confirmed that these transcripts of LINC01235 lack coding potential (Figure S2d,e, Supporting Information). The Pipeline for lncRNA annotation from RNA‐seq data (PLAR)^[^ ^23^ ^]^ also indicates that LINC01235 is not conserved across organisms, suggesting recent human‐specific evolutionary developments (Table S3, Supporting Information). FISH and cell fraction RNA analyses demonstrated that LINC01235 was expressed in both the cytoplasm and nucleus (**Figure** **2**a,b). We established cell lines with ectopic expression of the full‐length LINC01235 isoform via lentiviral infection, and a constitutively expressed doxycycline (dox)‐induced LINC01235 knockdown was generated using CRISPR‐Cas9 (Figure S2f, Supporting Information). HCC1954 was selected for knockdown and knockout experiments due to its high LINC01235 expression, while BT474, SK‐BR‐3, and JIMT1, with lower expression levels, were used for overexpression studies, all of which are HER2‐positive cell lines. We use MCF10A as normal cells. Our findings suggest that LINC01235 promotes the proliferation and clone formation of breast cancer cells (Figure 2c,d; Figure S2g, Supporting Information). In vivo, xenograft models showed that LINC01235 knockdown significantly inhibited the growth of HCC1954 xenografts (Figure 2e), whereas doxycycline treatment accelerated tumor growth in tet‐LINC01235 transfected JIMT1 xenografts (Figure 2f). Additionally, we developed antisense oligonucleotides (ASO) to target LINC01235. An In vitro study demonstrated the efficacy of ASO‐2 and ASO‐5(Figure S2h, Supporting Information). Next, we combined these two ASOs to treat LINC01235 highly expressed HCC1954 cells. Consistent with the above results, in vivo LINC01235 knockdown through ASO tail vein injection suppressed tumor growth (Figure 2g). Additionally, we treated human HER2+ breast cancer organoids with ASO‐NC or ASO‐1235, alongside Paclitaxel, lapatinib, and a DMSO control. Tumor growth, assessed by measuring the live organoid area, further confirmed that LINC01235 knockdown enhanced the sensitivity of organoids to drug treatment (Figure 2j).

To assess LINC01235's role in multi‐drug resistance, we measured the half‐maximal inhibitory concentration (IC50) values of lapatinib, pyrotinib, neratinib, T‐DXd, and paclitaxel in cells with high and low LINC01235 expression. LINC01235 overexpression conferred resistance to these drugs, whereas knockdown (KD) cells exhibited increased sensitivity (Figure 2i; Figure S2i,j, Supporting Information). Next, we explored whether LINC01235 expression was increased after treatment. We conducted a clonal competition assay with dox‐induced overexpression of LINC01235. Cells with high LINC01235 expression coexpressed with eGFP, whereas empty vector (EV) control cells coexpressed with mCherry. After doxycycline induction, the proportion of green clones significantly surpassed that of red clones treated with lapatinib and paclitaxel (Figure 2h), suggesting that LINC01235‐overexpressing clones promoted clonal expansion and treatment resistance. Since the efficacy of trastuzumab depends on antibody‐dependent cellular cytotoxicity, we monitored the growth of dox‐induced LINC01235‐overexpressing cells following treatment with trastuzumab and paclitaxel in vivo (Figure 2k). Notably, LINC01235‐overexpressing cells were more resistant to combination therapy than non‐overexpressing ones.

To further evaluate the clonal selection advantage of LINC01235‐overexpressing cells, we injected a 1:1 mixture of wild‐type and dox‐induced LINC01235‐GFP JIMT1 cells to form xenografts. After two weeks, the tumors were randomized by size and treated with paclitaxel and trastuzumab. Consistent with the organoid results, LINC01235‐overexpressing cells showed a higher treatment resistance. Post‐treatment, tumor analysis by immunohistochemistry (IHC) revealed a higher proportion of GFP+ cells in the induced group than in the controls, indicating the selective enrichment of LINC01235‐expressing cells (Figure 2l).

These data suggest that LINC01235 overexpression selects treatment‐resistant cells in vivo and in vitro.

### 2.3 LINC01235 Promotes Cell Cycle Progression through DNA Licensing

**Figure 3:**
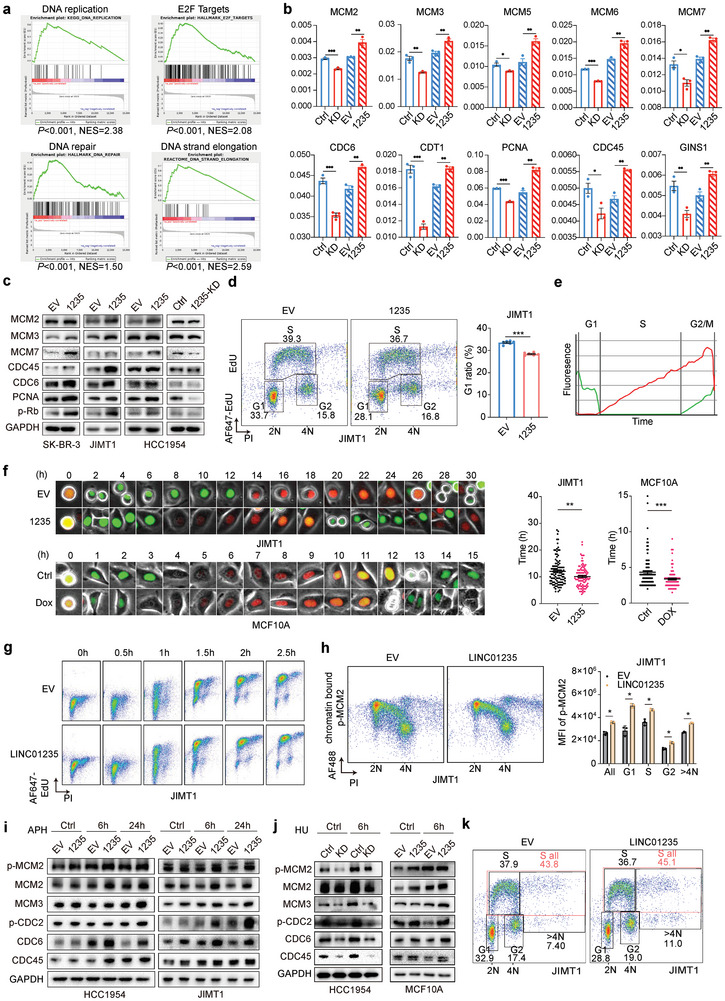
LINC01235 promotes cell cycle progression through DNA licensing. a) Gene Set Enrichment Analysis (GSEA) showed the indicated gene signatures in LINC01235‐overexpressing BT474 cells compared to empty vector control. NES, normalized enrichment score. b) RNA levels of DNA replication licensing factors in HCC1954 cells were analyzed by RT‐qPCR. Glyceraldehyde‐3‐phosphate dehydrogenase (GAPDH) was used as a reference gene. LINC01235 knockdown cells (KD) were compared with the control (Ctrl). LINC01235 (1235) overexpression compared with empty vector (EV) (Figure S3a, Supporting Information). c) Immunoblot showing indicated DNA replication licensing‐related proteins in SK‐BR‐3, JIMT1, and HCC1954 cells (Figure S3b, Supporting Information). d) Flow cytometry analysis of cell cycle in JIMT1 cells. G1 ratios were calculated as shown. *n* = 6 biological replicates. (See Figure S3d, Supporting Information for more details). e) Schematic view of cell cycle phase against fluorescence intensity using PIP‐FUCCI. f) Selected images from wide‐field time‐lapse imaging of one cell. Images were captured every 30 min for one cell cycle. JIMT1 and MCF10A cells were analyzed. Doxycycline (dox)‐induced MCF10A cells were treated with dox (500 ng ml^−1^) or PBS for 48 h before imaging. Statistical analyses were performed for the length of the G1 phase. *n* = 100. g) Flow cytometric analysis of double thymidine blockade and released JIMT1 cells at the indicated time. Cell cycles were analyzed by staining with propidium Iodide and Click‐it AF647 EdU (See Figure S3e, Supporting Information for more details) h) Flow cytometric analysis of chromatin‐bound phosphorylated MCM2 and cell cycles in JIMT1 cells. Cell cycle determination is the same as in Figure 3d. Mean fluorescence intensities (MFI) were calculated according to the cell phases. i) Immunoblot showing indicated proteins in JIMT1 and HCC1954 cells overexpressing EV and LINC01235 treated with aphidicolin (APH, 1 µm) or control for 6 and 24 h (See Figure S3g, Supporting Information for more details). j) Immunoblot showing indicated proteins in HCC1954 and MCF10A cells treated with hydroxyurea (HU, 10 mm) or control for 6 h. LINC01235 knockdown compared to control. LINC01235 overexpression compared to empty vector. k) Flow cytometric analysis of cell cycle in JIMT1 cells overexpressing LINC01235 or EV. *n* = 3 biological replicates. Statistical analyses are shown in Figure S3h (Supporting Information). For (a), (c–g), and (i,j), *n* = 2 experiments. For (b), (d–f), and (h), statistical analysis was performed using a two‐sided *t*‐test. * *p *< 0.05, ** *p *< 0.01, *** *p *< 0.001. Error bars represent mean ± SEM. (See Figure S3, Supporting Information).

To elucidate the role of LINC01235 in promoting tumor progression, we used RNA sequencing to explore the pathways that might be modulated by this lncRNA. Our analysis revealed that genes involved in DNA replication, E2F targeting, DNA repair, and DNA strand elongation were markedly overexpressed in breast cancer cells with high levels of LINC01235 both in vitro and in vivo (**Figure** **3**a–c; Figure S3a–c, Supporting Information), suggesting its pivotal role in the regulation of DNA replication and cell cycle dynamics. Notably, flow cytometric analysis demonstrated that LINC01235 significantly reduced the percentage of cells in the G1 phase, while causing a slight increase in the percentage of cells in the S phase (Figure 3d; Figure S3d, Supporting Information), potentially indicating altered cell phase timing. To substantiate these observations, we used a PCNA‐interacting protein (PIP)‐degron‐based Fluorescent Ubiquitination‐based Cell Cycle Indicator (FUCCI) reporter system (Figure 3e). This innovative system facilitates precise visualization of cell cycle transitions in live cells. Live‐cell imaging and tracking revealed that LINC01235 overexpression shortened the G1 phase duration in both normal (MCF10A) and tumor mammary cells (Figure 3f), supporting its influence on cell cycle regulation.

Given that previous research has linked the relationship between truncated G1 phase and premature origin licensing, we explored whether LINC01235 influences replication origins. By employing a double‐thymidine block to synchronize cells at the G1/S transition, followed by release, we observed that LINC01235 hastened G1 to S phase progression, with only a minor reduction in S phase duration (Figure 3g; Figure S3e, Supporting Information). The initiation of DNA replication involves the attachment of ORCs to replication origins, followed by the binding of licensing factors CDC6 and CDT1 and the subsequent formation of a heterohexameric MCM2‐7 complex. Origin activation during the S phase necessitates the binding of CDC45 and the GINS complex to MCMs, establishing the CMG (Cdc45, Mcm2–7, GINS) helicase. Additionally, MCM2‐7 is phosphorylated by CDC7. Our findings demonstrate that genes regulating the pre‐replicative complex and licensing factors were markedly increased following LINC01235 overexpression and decreased upon its knockdown, signifying a potential regulatory role of LINC01235 in DNA replication and cell cycle progression. Furthermore, we assessed chromatin‐bound phosphorylated MCM2 (p‐MCM2) using flow cytometry and observed elevated levels of p‐MCM2 (Figure 3h; Figure S3f, Supporting Information).

To further examine LINC01235's role in replication licensing, we used aphidicolin to inhibit RNA polymerase activity and observed the expression of licensing factors in both normal and cancer cells post‐treatment. Despite this inhibition, LINC01235 upregulated the phosphorylation of MCM2 (Figure 3i; Figure S3g, Supporting Information). Additionally, the expression levels of MCM2 and phosphorylated CDC2, CDC6, and CDC45 increased, implying that LINC01235 overexpression could promote DNA licensing even under replication stress (Figure 3i; Figure S3g (, Supporting Information). The use of hydroxyurea (HU) to halt DNA replication provided further evidence; following HU treatment, knockdown of LINC01235 significantly counteracted HU‐induced overexpression of p‐MCM2, MCM2, CDC6, and CDC45 (Figure 3j).

In addition to the increase in licensing proteins bound to chromatin, flow cytometric analysis of DNA content and synthesis revealed that LINC01235 significantly increased the proportion of cells with DNA content greater than twice the normal (>4 N), indicating the promotion of DNA re‐replication by LINC01235 (Figure 3k; Figure S3h,I, Supporting Information). These findings suggest that LINC01235 enhances DNA licensing during the G1 phase, leading to re‐replication in the S phase, thereby potentiating dysregulated origin firing in the context of DNA replication stress.

### 2.4 LINC01235 Increases Chromosomal Instability and Polyclonality in Breast Cancer

**Figure 4:**
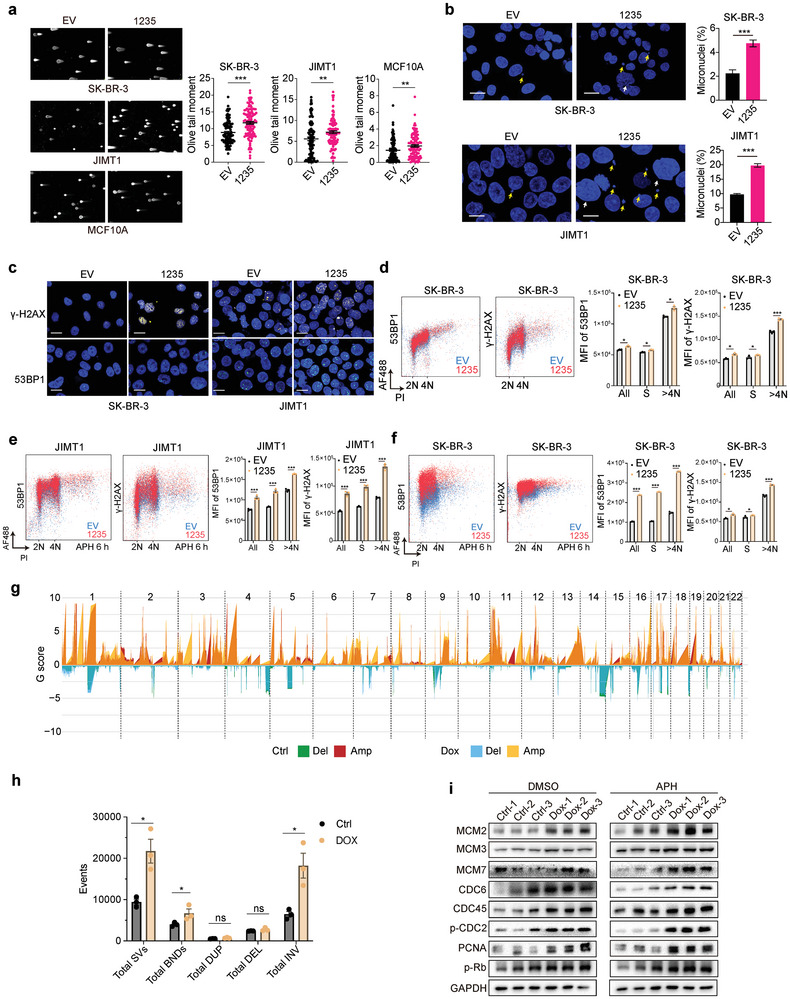
LINC01235 increases chromosomal instability and polyclonality in breast cancer. a) Neutral comet assays showed DNA breaks in SK‐BR‐3, JIMT1, and MCF10A cells overexpressed with empty vector (EV) or LINC01235 (1235). Representative comet images are shown on the top. Tail moments were quantified in 100 cells from two independent experiments using CaspLab software. b) Micronuclei analysis in SK‐BR‐3 and JIMT1 cells. Yellow arrows in representative images show the micronuclei. The bar graph represents the percentage of micronuclei‐containing cells after counting 100 cells. *n* = 3 biological replicates. c) Images of 53BP1 and γ‐H2AX immunostaining in SK‐BR‐3 and JIMT1 cells with overexpressed 1235 or EV. *n* = 3 experiments. d) Flow cytometric analysis of chromatin‐bound γ‐H2AX and 53BP1 in SK‐BR‐3 cells with overexpressed 1235 or EV. The bar graph represents the MFI of each protein. Cell cycle phases were determined by Click‐it AF488 EdU and propdium iodide (PI) staining. *n* = 3 biological replicates. e,f) After 1 µm aphidicolin (APH) treatment for 6 hours, JIMT1 (e) and SK‐BR‐3 (f) cells with LINC01235 overexpression were analyzed with the same conditions as in Figure 4d. *n* = 3 biological replicates. g) copy number variation (CNV) analysis of whole genomic sequencing data. Xenografts from doxycycline (dox) induced LINC01235 JIMT1 tumors compared to non‐induced (Ctrl) LINC01235 tumors were used. Deletions (del) and amplifications (amp) are shown in the graph. *n* = 3 biological replicates. h) The bar plot shows structural variation events in dox and Ctrl tumors of (g). BND, break‐end; DUP, duplication; DEL, deletion; INV, inversion. i) Immunoblot showing indicated proteins in cells dissociated from dox and Ctrl xenografts of Figure 4g. Either group was not treated with dox when cultured in vitro. APH or DMSO mock controls were given for 24 h before protein extraction. For (a), (b), (d–f), and (h), statistical analysis was performed using a two‐sided *t‐*test. * *p *< 0.05, ** *p *< 0.01, *** *p *< 0.001, ns, not significant. Error bars represent mean ± sem (standard error of the mean).

Excessive and dysregulated origin firing can lead to DNA replication stress, potentially resulting in DNA damage, copy number variation, and chromatin instability. Given the role of LINC01235 in DNA licensing, we hypothesized that its upregulation in breast cancer cells might contribute to chromosomal instability. To verify this assertion, neutral comet assays were conducted following LINC01235 overexpression in both normal and cancerous breast cancer cell lines (**Figure** **4**a), which revealed a marked increase in double‐stranded DNA breaks (DSBs). Previous reports have shown that micronuclei arise from genomic and chromosomal instabilities and cause aneuploidy and chromothripsis.^[^ ^24^ ^]^ This study found that LINC01235 overexpression significantly increases the occurrence of micronuclei, deformed nuclei, and the formation of γ‐H2AX and 53BP1 foci within cells (Figure 4b,c), further indicating that LINC01235 could upregulate chromosomal instability.

To further quantify the expression of γ‐H2AX and 53BP1, we employed flow cytometry to examine these chromatin‐bound DNA damage markers. Our findings revealed that LINC01235 significantly elevated the binding capacity of γ‐H2AX and 53BP1 to chromatin, particularly in cells with DNA content >4 N (Figure 4d). This enhancement was more pronounced under DNA replication stress induced by aphidicolin (Figure 4e,f). Additionally, western blotting and comet assays corroborated these results, further substantiating the role of LINC01235 in amplifying DNA damage under replication stress (Figure S4a,b, Supporting Information).

To further explore how the promotion of DNA damage by LINC01235 could increase chromosomal instability (CIN), we sequenced the whole genome of mouse tumors from JIMT1 xenografts. After doxycycline induction, the LINC01235 overexpression group exhibited significantly higher copy number variations (CNVs) across all chromosomes than the control group, further supporting the notion that LINC01235 promotes CIN in breast cancer (Figure 4g,h). To explore whether LINC01235's regulation of CIN contributes to tumor heterogeneity and the emergence of drug‐resistant subclones, we processed tumors collected from dox‐induced LINC01235 and control JIMT1 xenograft models. During the in vitro culture, neither cell group was treated with doxycycline. Western blotting analysis showed that DNA replication‐ and licensing‐related proteins were significantly upregulated in the dox‐treated group in vivo, although LINC01235 expression levels remained constant between the two groups (Figure 4i).

These findings indicate that under replication stress, the overexpression of LINC01235 enhances CIN and induces tumor heterogeneity, potentially enabling subclones to gain survival advantages.

### 2.5 LINC01235 Regulates H2A.Z and H3K27ac Modifications on the Chromosome

**Figure 5:**
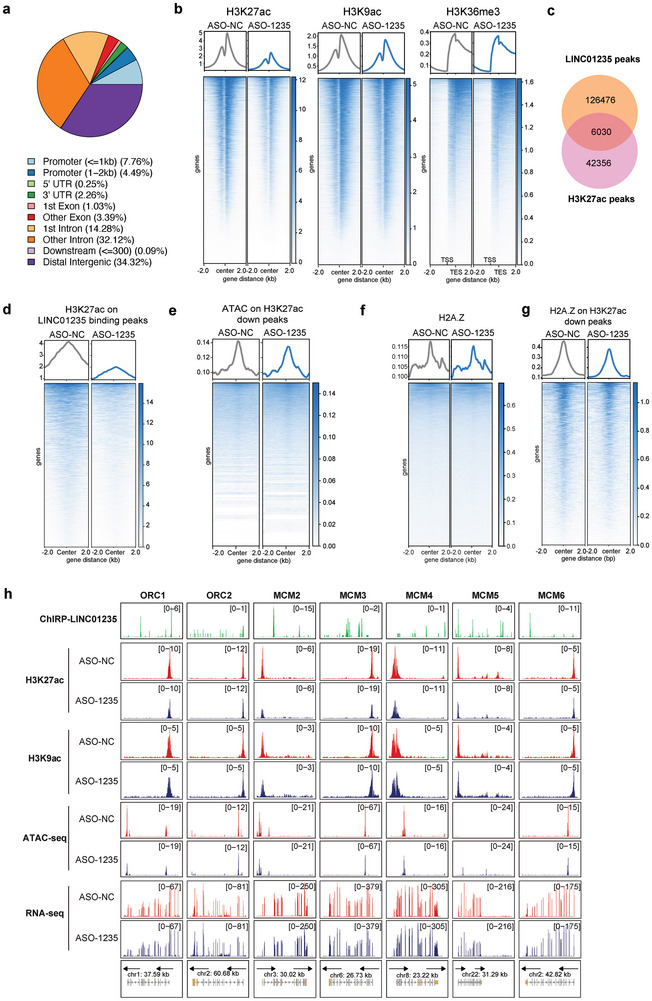
LINC01235 regulates H2A.Z and H3K27ac modifications on the chromosome. a) Genomic distribution of regions showing significantly increased ChIRP‐seq signal of HCC1954 cells. b) Heatmaps of all H3K27ac, H3K9ac, and H3K36me3 ChIP‐seq signals with or without LINC01235 depletion (top heatmap panel). c) Number of peaks of LINC01235 ChIRP‐seq and H3K27ac peaks in control HCC1954 cells. A total of 6030 peaks were co‐localized. d) Heatmaps of H3K27ac peaks in the co‐localized regions of Figure 5c. e) Heatmaps of ATAC‐seq signals on H3K27ac downregulated regions after LINC01235 depletion. f) Heatmaps of all H2A.Z ChIP‐seq signals with or without LINC01235 depletion. g) Heatmaps of H2A.Z signals on H3K27ac downregulated regions after LINC01235 depletion. h) Representative LINC01235 ChIRP‐seq, H3K27ac, and H3K9ac ChIP‐seq tracks, ATAC‐seq, and RNA‐seq tracks of the gene bodies of indicated genes in cells with or without LINC01235 depletion. The schematic views of the gene body are shown (bottom panel).

To explore the regulatory role of LINC01235 at replication origins, we used Chromatin Isolation by RNA Purification sequencing (ChIRP‐seq) to identify its binding sites across chromosomes. The ChIRP‐seq analysis in this study revealed that LINC01235 binds to genome‐wide promoter regulatory regions at a relatively high level, accounting for 12.25% of the total binding sites, indicating a significant regulatory role (**Figure** **5**a). To further characterize the distribution of LINC01235's binding sites, we used the Toolkit for Cistrome Data Browser to identify potential transcription factors and histone modification markers associated with LINC01235. Analysis of the 20 000 most differentially enriched peaks showed a substantial overlap of LINC01235 binding sites with key factors, such as POLR2A, EP300, H2AFZ, and histone modifications, including H3K36me3, H3K9ac, and H3K27ac (Figure S5a, Supporting Information), indicating that LINC01235 primarily binds to transcriptionally active and euchromatin regions.

Subsequently, we performed ChIP‐seq analysis to determine whether LINC01235 regulated the modifications of H3K27ac, H3K9ac, and H3K36me3. We found that the knockdown of LINC01235 reduced these epigenetic markers on the chromatin (Figure 5b; Figure S5b, Supporting Information), with a particularly notable reduction in H3K27ac. This marker distinguishes active enhancers from poised enhancers and is correlated with chromatin openness. We further investigated H3K27ac levels at LINC01235 binding sites and identified 6030 peaks bound to both LINC01235 and H3K27ac (Figure 5c; Figure S5c, Supporting Information). Among these shared peaks, we observed significantly reduced H3K27ac levels following LINC01235 knockdown (Figure 5d), indicating that LINC01235 may directly regulate H3K27ac in these regions. To investigate the role of LINC01235 in regulating chromatin accessibility at H3K27ac‐downregulated peaks, we conducted ATAC‐seq analysis. This analysis revealed that LINC01235 knockdown decreased the accessibility of LINC01235‐regulated genes (Figure 5e), reinforcing its role in modulating chromatin structure.

To unveil the regulatory role of LINC01235 in licensing DNA replication, we focused on the histone marker H2A.Z, which has been shown to significantly overlap with those involved in the initiation of DNA replication.^[^ ^25^ ^]^ We found that LINC01235 globally downregulated H2A.Z levels, specifically in the LINC01235‐regulated regions (Figure 5f,g). These results indicate that LINC01235 promotes DNA replication by regulating chromatin accessibility.

In addition to directly regulating LINC01235 binding regions, we investigated how LINC01235 regulates licensing factors at the epigenetic level. Consistent with the RNA‐seq data, we found that the promoter regions of DNA licensing factors such as ORC1, ORC2, and MCM2‐6 were regulated by LINC01235 (Figure 5h). These findings indicated that LINC01235 promotes the overexpression of these genes by upregulating chromatin accessibility, thereby enhancing DNA licensing and cell proliferation.

In summary, LINC01235 regulates DNA replication by upregulating licensing factors and increasing chromatin accessibility by enhancing H3K27ac.

### 2.6 LINC01235 Promotes Chromosome Instability by Interacting with XRCC5

**Figure 6:**
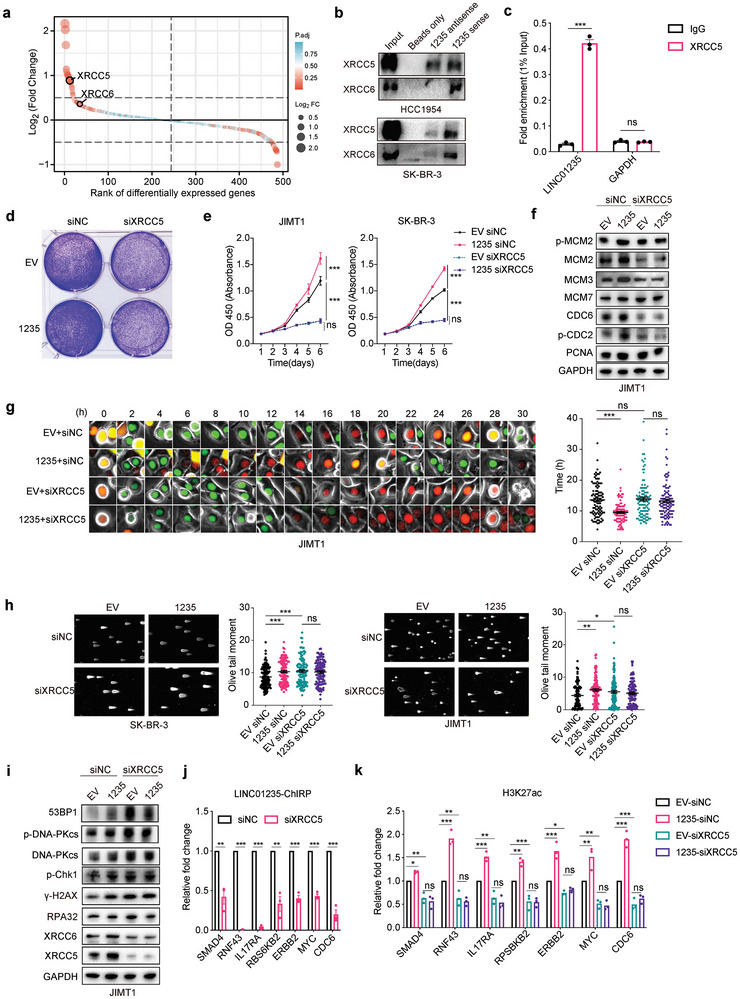
LINC01235 promotes chromosomal instability by interacting with XRCC5. a) Distribution of log2 fold change for proteins enriched after LINC01235 RNA pull‐down compared to the control. TMT‐labeled quantification methods were used. *n* = 3 biological replicates for each group. b) immunoblot of XRCC5 and XRCC6 after LINC01235 RNA pull‐down with the sense and antisense strand of RNA in HCC1954 and SK‐BR‐3 cells. *n *= 2 experiments. c) RNA immunoprecipitation with XRCC5 and IgG quantified by RT‐qPCR was performed in HCC1954 cells. *n *= 3 biological replicates. d) clonal formation assay of JIMT1 cells with the depletion of XRCC5 or control after overexpression with empty vector (EV) or LINC01235 (1235). *n* = 2 experiments. e) Cell proliferation assay of LINC01235 (1235) overexpression compared to empty vector (EV) in JIMT1 and SK‐BR‐3 cells. Cells were also treated with siRNA targeting XRCC5 or non‐targeting negative controls (NC). *n* = 2 experiments. *P*‐values were calculated using a one‐way analysis of variance (ANOVA) at the endpoint. f) Immunoblot showing indicated DNA replication licensing‐related proteins in JIMT1 cells overexpressing EV and LINC01235 treated with siXRCC5 or siNC for 72 h (See Figure S6c, Supporting Information for more details). g) Selected images from wide‐field time‐lapse imaging of one cell. Images were captured every 30 min for one cell cycle. JIMT1 cells with the depletion of XRCC5 or control after overexpressing empty vector (EV) or LINC01235 (1235) were assayed. Statistical analyses were performed for the length of the G1 phase. *n* = 100. *P*‐values were calculated using one‐way ANOVA. h) Neutral comet assays showed DNA breaks in SK‐BR‐3 and JIMT1 cells overexpressing EV or 1235 after treatment with siNC and siXRCC5. Representative comet images are shown on the top. Tail moments were quantified in 100 cells from two independent experiments using CaspLab software. *P*‐values were calculated using one‐way ANOVA. i) Immunoblot showing indicated DNA damage proteins in JIMT1 cells overexpressing EV and LINC01235 treated with siXRCC5 or siNC for 72 h. *n* = 2 experiments. j) LINC01235 ChIRP‐qPCR results with or without XRCC5 depletion in HCC1954 cells. Seven genes with LINC01235 binding at the promoter regions were selected. The data represents the fold change of peak enrichment relative to siNC cells. *n* = 3 biological replicates. *P* values were calculated using multiple unpaired *t*‐tests. k) H3K27ac ChIP‐qPCR results were analyzed in HCC1954 cells. *n *= 3 biological replicates. *P*‐values were calculated using one‐way ANOVA. * *p *< 0.05, ** *p *< 0.01, *** *p *< 0.001. Error bars represent mean ± SEM (standard error od mean).

To delineate the specific molecular interactions of LINC01235, we employed RNA pull‐down assays coupled with tandem mass tag (TMT) proteomics to quantitatively detect proteins associated with LINC01235. We established a 1.2‐fold change threshold and a *p‐*value below 0.05 to discern significant interactions between proteins and LINC01235. Our comprehensive analysis identified 72 differentially interacting proteins enriched in pathways related to RNA splicing, protein translation, and DNA double‐strand unwinding (**Figure** **6**a; Figure S6a, Supporting Information).

Of particular interest was the discovery of a substantial interaction between LINC01235 and the XRCC5/XRCC6 complex, which was corroborated by western blot analysis (Figure 6b). Further validation was performed using RNA immunoprecipitation assays, which confirmed an interaction between XRCC5 and LINC01235 (Figure 6c). This direct association between LINC01235 and components of the DNA repair machinery underscores the potential mechanistic pathways through which LINC01235 may influence genomic stability and cellular processes related to cancer progression.

The Ku heterodimer composed of Ku70 and Ku80 subunits encoded by XRCC6 and XRCC5, respectively, is a multifunctional complex that is pivotal in various cellular processes, such as non‐homologous end joining (NHEJ), V(D)J recombination, apoptosis, telomere maintenance, and DNA replication. The interaction of Ku protein with components of DNA replication‐origin licensing proteins, including DNA polymerase, proliferating cell nuclear antigen (PCNA), topoisomerase II, replication factor C (RFC), and replication protein A (RPA), has been well documented, signifying its integral role in the formation of the pre‐replication complex.

Our study initially revealed that the modulation of XRCC5 expression, either by knockdown or overexpression, did not significantly alter LINC01235 levels (Figure S6b, Supporting Information). Conversely, LINC01235 overexpression led to a significant increase in both XRCC5 mRNA and protein expression, indicating a regulatory effect of LINC01235 on XRCC5 (Figure 6i; Figure S6b, Supporting Information). To assess the dependence of LINC01235 function on XRCC5, we overexpressed LINC01235 in the context of XRCC5 knockdown using siRNA. These experiments showed that LINC01235's ability to enhance cell proliferation and colony formation was impeded when XRCC5 was downregulated (Figure 6d,e).

Furthermore, we evaluated the effect of XRCC5 on the regulation of replication‐licensing protein expression and found that XRCC5 knockdown could reduce the upregulation of these markers by LINC01235 (Figure 6f; Figure S6c, Supporting Information). Next, we utilized the PIP‐FUCCI system to measure the duration of the G1 phase in cells with XRCC5 knockdown (Figure 6g). The results indicated that XRCC5 knockdown alone did not alter the G1 phase length; however, in cells overexpressing LINC01235, XRCC5 knockdown led to a considerable extension of the G1 phase compared to the controls. This suggests that the G1 phase shortening mediated by LINC01235 is contingent upon the presence of XRCC5, reinforcing the fact that LINC01235 exerts its influence on the cell cycle through XRCC5.

To further substantiate the role of XRCC5 in mediating the effects of LINC01235 on DNA damage, we conducted comet assays using SK‐BR‐3 and JIMT1 breast cancer cells. Silencing XRCC5 via small interfering RNA (siRNA) significantly increased the number of DSBs in control cells (Figure 6h). Interestingly, in cells overexpressing LINC01235, siRNA‐mediated knockdown of XRCC5 did not exacerbate DNA damage, suggesting that XRCC5 does not mitigate the LINC01235‐induced DNA damage. This was further corroborated by Western blotting analysis, which indicated significant upregulation of γ‐H2AX, phosphorylated DNA‐PKcs (p‐DNA‐PKcs), and 53BP1 following XRCC5 knockdown (Figure 6i). However, LINC01235 overexpression did not increase the levels of these markers.

These findings demonstrate that LINC01235's ability to shorten the G1 phase depends on the functional presence of XRCC5. Moreover, XRCC5 appeared to play a protective role against the cytotoxic effects of LINC01235‐induced DNA damage, highlighting the complex interplay between LINC01235 and XRCC5, which affects both cell cycle progression and genomic stability in breast cancer cells.

To assess whether LINC01235's genomic binding relies on XRCC5, we performed ChIRP‐qPCR analyses following XRCC5 knockdown using siRNA. The results indicated a significant reduction in LINC01235 enrichment peaks upon XRCC5 depletion, underscoring the dependence of LINC01235's genomic association on XRCC5 (Figure 6j; Figure S6d, Supporting Information). Additionally, to determine whether changes in histone modification mediated by LINC01235 also depend on XRCC5, we overexpressed LINC01235 in JIMT1 cells while concurrently knocking down XRCC5. The ChIP‐qPCR results showed that LINC01235 overexpression elevated the levels of H3K27ac and H2A.Z modifications; however, these enhancements were mitigated by XRCC5 knockdown (Figure 6k; Figure S6e, Supporting Information). Notably, the overexpression of LINC01235 after XRCC5 knockdown did not restore the modification levels of H3K27ac and H2A.Z, suggesting that LINC01235's effects on chromatin openness and its ability to bind to the genome are dependent on XRCC5 (Figure 6k; Figure S6e, Supporting Information). This interdependence highlights a complex interaction between LINC01235 and XRCC5, which is crucial for regulating DNA replication through the modification of chromatin architecture.

### 2.7 Exploring Potential Therapeutic Strategies to Target LINC01235‐Overexpressing Cancers

Given that LINC01235 promotes cell cycle progression and chromosomal instability, we hypothesized that cells overexpressing LINC01235 would be particularly susceptible to DNA damage repair pathway inhibitors, leading to synthetic lethality. Therefore, we tested the sensitivity of these cells to PARP1 inhibitors, such as olaparib, which have demonstrated clinical efficacy in tumors deficient in homologous recombination (HR). Notably, LINC01235‐overexpressing cells exhibited marginal sensitivity to olaparib (Figure S7a, Supporting Information).

**Figure 7:**
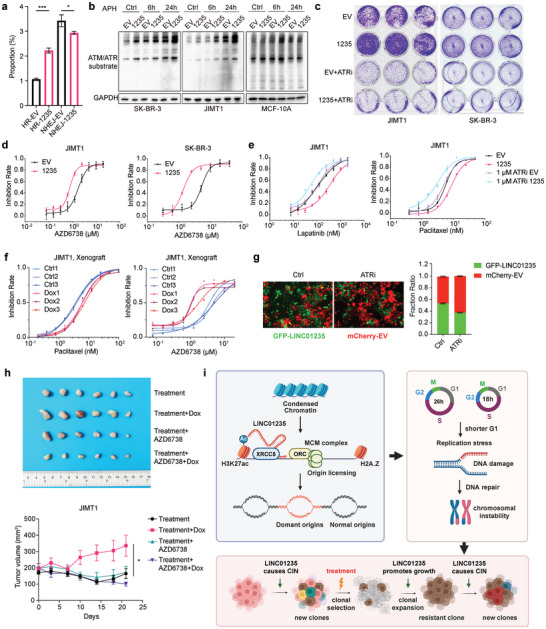
Exploring potential therapeutic strategies to target LINC01235‐overexpressing cancers. a) Homologous recombination (HR) and non‐homologous end joining (NHEJ) efficiency were determined by flow cytometric analysis of the % of GFP+ (HR) and mCherry+ (NHEJ) cells, respectively. *n* = 3 biological replicates. *P*‐values were calculated using a two‐sided *t*‐test. b) Immunoblot of ATM/ATR substrate after the cells were treated with 1 µm aphidicolin for the indicated time. *n* = 2 experiments. c) Clonal formation assay of JIMT1 and SK‐BR‐3 cells treated with ATR inhibitor. *n* = 2 experiments. d) Growth inhibition assays of ATR inhibitor AZD6738 were performed in JIMT1 and SK‐BR‐3 cells. *n *= 3 for each concentration. e) Growth inhibition assays of lapatinib and paclitaxel in LINC1235‐overexpressing and empty vector JIMT1 cells. AZD6738 was given as indicated. *n* = 3 for each concentration. f) Growth inhibition assays of cells dissociated from xenografts from Figure 2f. JIMT1 cells overexpressing dox‐induced LINC01235 were injected into nude mice. Xenografts were either induced with dox or not. The cells were cultured after dissociation without dox induction. Paclitaxel and AZD6738 were given as indicated. g) clonal competition assay between GFP‐LINC01235 and mCherry‐EV JIMT1 cells. The growth was monitored using Cytation5. AZD6738 (1 µm) was given for 3 days. The ratio of GFP and mCherry‐positive cells was analyzed. *n* = 3 biological replicates. h) Analysis of tumor volume in nude mice subcutaneously injected with JIMT1 dox‐induced 1235 cells. Mice were divided into four groups based on size. Doxycycline (dox) diets were given as indicated. Paclitaxel and trastuzumab were given as described in Figure 2. AZD6738 (50 mg k^−1 ^g) were gauzed daily. The upper panel showed the tumors dissected at the endpoint and sorted by group. The lower panel showed the growth curve of the tumors. *n* = 6 for each group. *P*‐values were calculated using a one‐way analysis of variance at the endpoint. i) graphical abstract of this study. * *p *< 0.05, *** *p *< 0.001. Error bars represent mean ± sem (standard error of the mean).

To determine whether LINC01235 regulates HR and NHEJ, we used a DSB repair reporter assay to assess the event rates of these two repairs.^[^ ^26^ ^]^ The results indicated that LINC01235 upregulated HR but had negligible effects on NHEJ (**Figure** **7**a). This suggests that LINC01235 may stimulate HR to counteract chromosomal instability‐induced cell death. To confirm the role of LINC01235 in promoting HR, we assessed the phosphorylation levels of ATM/ATR (ataxia‐telangiectasia, mutated/ATM and Rad3‐related) substrates in cells with high versus normal LINC01235 expression, particularly those under replication stress conditions. We observed a significant upregulation in the phosphorylation of ATR substrates in LINC01235‐overexpressing cells (Figure 7b), indicating the activation of the ATR repair pathway, which may contribute to the survival and proliferation of these cancer cells.

ATR plays a crucial role in the regulation of the HR, replication fork stability, and late‐origin firing. It functions in the nucleus to prevent micronucleus (MN) formation and within MNs to promote their removal, thereby preventing aneuploidy and chromothripsis. To investigate the vulnerability of cells with high LINC01235 expression to ATR inhibitors (ATRi), we used AZD6738 to treat breast cancer cells with different LINC01235 expression levels. We found that ATRi effectively suppressed tumor colony formation and eliminated the growth advantage of LINC01235 (Figure 7c). IC50 experiments further demonstrated that ATRi mitigated the pro‐proliferative effects of LINC01235 and effectively reversed the drug‐resistant phenotype induced by this lncRNA (Figure 7d,e). We used cells dissociated from dox‐induced LINC01235 and control JIMT1 xenograft models. Cells collected from dox‐induced LINC01235 tumors have been shown to be resistant to paclitaxel treatment but sensitive to ATRi (Figure 7f). In addition, a clonal competition assay showed that LINC01235‐overexpressing cells were sensitive to ATR inhibitors (Figure 7g).

To further validate the sensitivity of tumors with high LINC01235 expression to ATR inhibition, we used JIMT1 dox‐induced LINC01235 mouse xenograft models to assess the efficacy of a combination therapy involving ATRis, trastuzumab, and paclitaxel. Consistent with previous experimental results, dox‐induced LINC01235 tumors exhibited resistance to both paclitaxel and trastuzumab (Figure 7h). Intriguingly, this resistance was counteracted by the addition of ATR inhibitors. Notably, cells not treated with dox for LINC01235 overexpression did not show sensitivity to ATR inhibitors, underscoring the specificity of the effects of ATRi in the context of LINC01235 overexpression.

These findings reinforce the therapeutic potential of ATR inhibitors, particularly in cancers characterized by the overexpression of LINC01235.

## 3 Discussion

HER2+ tumors exhibit inherent heterogeneity.^[^ ^27^ ^]^ This heterogeneity is spatial owing to the presence of multiple intrinsic clones, including resistant cells, and temporal, based on the clonal evolution and selection that occurs following its development and treatment. Treatment and environmental pressures selectively enhance the survival of resistant subclones, which acquire new phenotypic traits that enable them to evade therapeutic targets and adapt more effectively to their surroundings. These factors may influence the response and resistance to HER2‐targeting therapies and chemotherapy, making HER2+ tumors an appropriate model for studying treatment resistance. In this study, we identified LINC01235 as a lncRNA that is specifically overexpressed in breast tissues and contributes to treatment resistance in HER2‐positive breast cancer. LINC01235 upregulates the MCM complex and other replication‐licensing proteins, such as CDC6 and CDT1, promoting tumor growth by enhancing the licensing of replication origins. Cells possess redundant DNA replication origins; however, not all licensed origins initiate replication at the onset of the S phase. Origins that are licensed, but remain dormant, can be activated to replace stalled replication forks, thus preserving the continuity of DNA synthesis.^[^ ^28^ ^]^ Induction of replication stress by chemotherapeutic or targeted therapies challenges cellular replication processes. Elevated MCM expression can alleviate such stress, facilitating continuous DNA replication, which, in turn, supports tumor progression and contributes to resistance against cancer therapies.

Excessive and dysregulated firing of the replication origins can precipitate re‐replication within the same cell cycle, leading to DNA damage and CIN. Studies have shown that oncogenes such as cyclin E and c‐myc can provoke CIN by disrupting origin firing.^[^ ^15c–e^ ^]^ This instability increases tumor cell diversity. Although many clones are deleterious and perish rapidly, surviving clones can swiftly adapt to various external pressures. Consequently, tumor cells can rapidly develop resistance to anticancer therapies and reduce their dependency on initial driver oncogenes. Notably, studies have shown that CIN can expedite adaptation to oncogene withdrawal in KRAS‐ and BRAF‐driven mouse models.^[^ ^29^ ^]^ In our study, prolonged overexpression of LINC01235 in tumors conferred a replication advantage, independent of its expression. These cells retained the features of LINC01235 overexpression, such as the upregulation of licensing factor, CIN, and treatment resistance. Therefore, in HER2‐positive breast cancer, LINC01235‐overexpressing clones inherently resist therapeutic interventions. After treatment, these resistant clones can rapidly evolve through CIN, potentially acquiring new survival advantages independent of LINC01235, highlighting the challenges of targeting dynamic oncogenic pathways in cancer therapy.

Our study demonstrated that LINC01235 modulates the expression of histone modifications, particularly H3K27ac, H3K9ac, and H3K36me3, at the chromatin level. Among these modifications, H3K27ac exhibited the most pronounced alterations following the knockdown of LINC01235, indicating its significant regulatory impact. It has been documented that H3K27ac is primarily localized to the promoter and enhancer regions, serving as a key marker of chromatin accessibility. It has been suggested that H2A.Z epigenetically regulates DNA replication licensing and the activation of early replication origins.^[^ ^25^ ^]^ In regions where H3K27ac is influenced by LINC01235, we noted significant downregulation of H2A.Z expression after the knockdown of LINC01235. This evidence supports the hypothesis that LINC01235 enhances chromatin accessibility by regulating H3K27ac, thereby promoting DNA replication licensing at the replication origins. These results highlight the essential function of LINC01235 in the dynamic regulation of chromatin architecture and its implications for cellular replication.

We discovered that LINC01235 binds to XRCC5, a key molecule in the NHEJ pathway. Although not primarily known for its role in DNA replication, XRCC5 contributes to various facets of the replication process, underscoring its supportive but crucial function in genomic stability.^[^ ^30^ ^]^ Initially identified for its role in yeast, where it modulates the timing of replication origin activation, particularly near telomeric regions,^[^ ^31^ ^]^ Ku80 also facilitates the loading of the pre‐replicative complex at replication origins.^[^ ^32^ ^]^ Its absence leads to reduced levels and chromatin binding of essential initiator proteins, such as Orc1, Orc4, Orc6, and Cdc45, resulting in decreased origin firing.^[^ ^32^ ^]^ In addition, Ku80 helps to stabilize replication processes at stalled forks, aids recovery and prevents excessive double‐strand break resection during replication‐associated damage.^[^ ^33^ ^]^ This limitation is the key to avoiding premature engagement with the homologous recombination repair pathway, thereby maintaining replication integrity and cell cycle progression. In our study, we found that LINC01235's function was mediated by XRCC5. After XRCC5 knockdown, the ability of LINC01235 to promote DNA replication licensing was lost, indicating that the function of LINC01235 is mediated by XRCC5.

In this study, we initially used ASOs‐targeting LINC01235, which demonstrated significant efficacy both in vitro and in vivo. However, this approach does not address the survival of evolved clones that operate independently of their original oncogenic drivers, as observed in our animal models. We discovered that LINC01235 circumvents DNA damage‐induced cell death by upregulating the ATR pathway, which drives homologous recombination. Given the established efficacy of ATR kinase inhibitors in tumors with BRCA1/2 loss and homologous recombination deficiency, we integrated ATR inhibitors with HER2‐targeted therapies to treat tumors overexpressing LINC01235. This combination effectively neutralized the growth advantage conferred by LINC01235 overexpression, thereby positioning ATR inhibitors as strategic targets for these tumors.

This study has certain limitations. Although HER2‐positive breast cancer was used as a model to investigate the role of LINC01235, and we confirmed its critical contribution to treatment resistance in this subtype, our findings suggest that the molecular mechanisms of LINC01235 may be independent of the HER2 protein or its associated pathways. Survival analyses demonstrated that LINC01235 is not only a significant predictor of prognosis in HER2‐positive breast cancer but also exhibits prognostic relevance in HR+/HER2‐ breast cancer, suggesting its functional impact is not confined to HER2‐positive disease. Mechanistically, LINC01235 was shown to promote genomic instability by enhancing replication licensing, a process observed even in HER2‐negative cells, such as MCF10A, which indicates that its underlying mechanism is likely conserved across different breast cancer subtypes. These observations highlight the potential broader implications of LINC01235 in breast cancer beyond HER2‐positive subtypes, as well as in other cancers. Future research is warranted to validate its role across different molecular subtypes of breast cancer and to investigate its contribution to resistance mechanisms in other cancers.

In conclusion, our study elucidated the mechanism underlying LINC01235‐driven treatment resistance in HER2‐positive breast cancer. LINC01235's involvement in DNA replication licensing and CIN facilitates clonal evolution and selection under treatment pressure. These findings suggest that targeting LINC01235‐related pathways with ATRi may be a viable strategy for clinical application, offering a potential avenue for overcoming resistance in this cancer subtype.

## 4 Experimental Section

### Ethics

The present study was approved by the Medical Ethics Committee (050432‐4‐1911D) and the Experimental Animal Ethics Committee (2020‐ZLYY‐JS‐297) of Fudan University Shanghai Cancer Center. The informed consent for the use of the sample for research purposes was obtained by participants (050432‐4‐1911D).

### Patient Samples

A consecutive cohort of 63 HR‐, HER2+ patients with breast cancer scheduled to start neoadjuvant treatment was assembled and named as Fudan University Shanghai Cancer Center (FUSCC) neoadjuvant chemotherapy (NAC) cohort. Core needle biopsy specimens were collected before treatment, and RNA was extracted and sequenced. Clinical features are detailed in Table S1 (Supporting Information). All patients received paclitaxel‐based chemotherapy regimens and trastuzumab‐based targeted therapy, with some patients additionally receiving pertuzumab. The neoadjuvant chemotherapy course consisted of 6–8 cycles. After treatment, all patients underwent surgery. Patients were classified into pathological complete response (pCR) and non‐pathological complete response (non‐pCR) groups based on their postoperative pathological results. pCR was defined as the complete remission of both the primary tumor site and axillary metastatic lymph nodes. Among the patients, 35 achieved pCR and 28 did not, resulting in an overall pCR rate of 55.5%. Differentially expressed genes were determined by Limma through using log2FC ≥ 2 and *P*‐value < 0.05 as the cut‐off values.

For the PHEDRA NAC cohorts, samples were collected from the Clinical Study of Neoadjuvant Pyrotinib, Trastuzumab, and Docetaxel for HER2‐positive Breast Cancer (PHEDRA) (NCT03588091), with the approval of the study protocol. A total of 25 HR‐, HER2+ patients were enrolled in this cohort. The patients were treated with pyrotinib + trastuzumab + docetaxel or trastuzumab + docetaxel for 4 cycles before surgery. There were 13 cases in the pCR group and 12 cases in the non‐pCR group, with an overall pCR rate of 52%. Differentially expressed genes were determined by log2FC ≥ 2 and *P*‐value < 0.05.

The prognosis cohort samples were collected from 319 patients with early breast cancer receiving surgical resection and adjuvant treatment from 2010 to 2011 as previously described.^[^ ^34^ ^]^ Briefly, tumor samples were collected after surgery and stored in liquid nitrogen. The survival analysis was performed by using Kaplan–Meier methods. Patients were analyzed (All types) or divided into subgroups according to ER, PR, and HER2 expression. HER2 was defined as IHC HER2 3+ or HER2 2+ with HER2 FISH amplification with estrogen receptor (ER) and progesterone receptor (PR) negative. TNBC was defined as IHC ER, PR, and HER2 negative. Luminal was defined as IHC ER+ or PR+. The mid‐split cutoff was used to categorize LINC01235 high and low expression. TCGA data were extracted and analyzed by GEPIA.^[^ ^35^ ^]^ Normal tissue RNA‐seq analysis were also performed by GEPIA by using GTEx data.

### Organoid Culture and Treatments

Organoid were harvested and cultured as previously described with minor modifications.^[^ ^36^ ^]^ Tissues were minced, washed with Advanced DMEM/F12 (Gibco, 12 634 028) containing 1× GlutaMAX (Gibco, 35 050 061), 10 mm HEPES (Gibco, 15 630 130) and Penicillin/Streptomycin (Gibco, 15 070 063), and digested in 10 mL Advanced DMEM/F12 containing 1% BSA (Basalmedia, S475T7), 10 µm Y‐27632 (Selleck, S1049), 1× Primocin (Invivogen, ant‐pm‐05), 1 mg ml^−1^ Collagenase Type I (Worthington, LS004194), 2 mg ml^−1^ hyaluronidase (Sigma, H3884) on an orbital shaker at 37 °C for 1–2 h. The suspension was strained over a 100 mm filter and centrifugation at 200 g for 10 min. The pellet was lysed in 2 mL ACK buffer (Gibco, A1049201) and centrifuged. The pellet was resuspended in 10 mg ml^−1^ cold Cultrex growth factor reduced BME type 2 (Trevigen, 3533‐010‐02) and 40 mL drops of BME‐cell suspension were allowed to solidify on culture plates at 37 °C for 20 min. Organoid medium were made with Advanced DMEM/F12 supplemented with 250 ng ml^−1^ R‐spondin1 (Peprotech, 120–38), 5 nm Neuregulin 1 (Peprotech, 100–03), 5 ng ml^−1^ FGF7 (Peprotech, 100–19), 20 ng ml^−1^ FGF10 (Peprotech, 100–26), 5 ng ml^−1^ EGF (Peprotech, AF‐100‐15), 100 ng ml^−1^ Noggin (Peprotech, 120‐10C), 0.5 mm A83‐01 (Selleck, S7692), 0.5 mm SB‐202190 (Selleck, S1077), 1.25 mm N‐Acetylcysteine (Sigma, A9165), 5 mm Nicotinamide (Sigma, N0636), 1× B27 (Gibco, 17 504 044), 1 mm HEPES, 1X GlutaMax, 1× Penicillin/Streptomycin, and 1× Primocin. The medium was exchanged every 3 to 4 days and cultures were passaged by incubating in dispase solution (20% FBS in dispase with Y‐27632), followed by a wash step with base medium and a dissociation step in TrypLE Express.

For 3 HER2+ organoids, 6 µg mL^−1^ trastuzumab (Selleck, A2007), 6 µg mL^−1^ pertuzumab (Selleck, A2008), and 50 nm paclitaxel (Selleck, S1150) were treated for 3 cycles (drugs were given on Day1 of each cycle and changed with normal media after 4 days. The media were changed regularly for a total of 3 weeks). Organoids were harvested and RNA was extracted at the baseline, after the first and third cycle.

For organoids in ASO treatment, 70 nm paclitaxel or 500 nm lapatinib were given in combination with 10 nmol mL^−1^ ASOs. For live/die quantifications, the Live Dead Cell Viability Assay Kit (Millipore) was used following the manufacturer's instructions. The area of Calcein AM+ organoids was quantified using ImageJ.

### Animal Studies

The designs of animal studies and procedures were approved by the Fudan University Shanghai Cancer Center (FUSCC) Institutional Animal Care and Use Committee (IACUC) (2020‐ZLYY‐JS‐297). Ethical compliance with IACUC protocols and institute standards was maintained. All mice were maintained under pathogen‐free conditions. The animal room had a controlled temperature (18–23 °C), humidity (40–60%), and a 12‐hour light/12‐hour dark cycle. In each experiment, animals were randomly assigned into each group. No blind was used in this study. 6–8 weeks‐old female nude mice were used in this study.

For in vivo tumor growth and survival analysis, 6–8 weeks‐old female nude mice were anesthetized and shaved at the injection site. HCC1954 cells (1 × 10^6^; LINC01235 knockdown and scramble control cells) or 1 × 10^6^ JIMT1 cells (dox‐induced LINC01235 cells) were resuspended in D‐PBS solution (Gibco), mixed 1:1 by volume with BME (3632‐010‐02, Cultrex) at a final concentration of 1 × 10^7^ ml^−1^, and subcutaneously injected into the fourth mammary fat pat.

For DOX inducement, a 2000 ppm doxycycline diet was given. The doxycycline diet and the normal diet were made by Jiangsu Xietong Pharmaceutical with the same formula except doxycycline addition.

For ASO treatment, ASO2 and ASO5 were mixed 1:1. 10 nmol ASO 2 and 5 mixture (5 nmol each) or NC‐ASO (10 nmol) were intravenously injected twice a week until the endpoint.

For trastuzumab and paclitaxel treatment, 10 mg k^−1 ^g trastuzumab and/or 20 mg k^−1 ^g paclitaxel were given at the indicated timepoint shown in the figures. Trastuzumab and paclitaxel were given intraperitoneally (i.p.). Trastuzumab was formulated in 5% dextrose Paclitaxel was formulated in Cremophor EL: Ethanol (1:1) and diluted in a sterile 0.9% NaCl solution as previously described.^[^ ^37^ ^]^

For the AZD6738 treatment experiment, mice were gauzed with 50 mg k^−1 ^g AZD6738 (Selleck, S7693) daily until the end point. AZD6738 was formulated in 10% DMSO/40% Propylene Glycol (Sigma‐Aldrich, P4347)/50% deionized water as previously described.^[^ ^38^ ^]^ Trastuzumab and paclitaxel were given twice a week as described above.

Tumor dimensions were measured with an electronic caliper every 2–3 days. Tumor volume was measured using the formula (length × width^2^/2), where length represents the largest tumor diameter and width represents the perpendicular tumor diameter. Mice were sacrificed when tumors reached 2000 mm^3^ or upon ulceration/bleeding. At the endpoint, animals were euthanized by CO_2_ and re‐checked by thoracic cavity opening following the protocol of IACUC. Tumors were dissected.

To obtain cells after the treatment or growth from xenografts, xenograft tumors were minced and digested following organoid protocols as described above. Cells were cultured at the same condition to their origin cell lines. Puromycin 2 µg ml^−1^ was added to kill fibroblasts for 2 weeks.

### Cell Iines and Culture Treatments

HCC1954 (ATCC, CRL‐2338), SK‐BR‐3 (ATCC, HTB30), BT474 (ATCC, HTB‐20) cells were obtained from the ATCC. MCF10A (SCSP‐575) was obtained from the National Collection of Authenticated Cell Cultures of China. JIMT1 was purchased from Procell (CL‐0770). BT‐474 were cultured in RPMI‐1640 medium (L210KJ, BasalMedia) supplemented with 10% FBS (10 099 141, Gibco). SK‐BR‐3 which were cultured in McCOY's 5A (L630KJ, BasalMedia) + 10% FBS. HCC1954 were cultured in ATCC‐modified RPMI 1640 (30‐2001, ATCC) + 10% FBS. MCF10A were cultured in DMEM/F12 (L310‐KJ, BasalMedia) + 5% Horse Serum (16 050 122, Gibco) + 20 ng ml^−1^ EGF(PHG0311, Gibco) + 100 ng ml^−1^ Cholera toxin (C‐8052, Sigma) + 1× Insulin‐Transferrin‐Selenium (Gibco) + 0.5 µg ml^−1^ hydrocortisone (H‐0888, Sigma) as described previously.^[^ ^39^ ^]^ 293FT cells were obtained from Thermofisher (R70007) and cultured in DMEM with 1× GlutaMax (Gibco, 35 050 061), 10 mm MEM NEAA (Gibco, 11 140 050), 100 mm Sodium Pyruvate (Gibco, 11 360 070), 10% FBS. All cells were maintained at 37 °C under 5% CO2. 1× Penicillin‐Streptomycin was added to all culture mediums (Gibco, 15 140 122). All cell lines were authenticated by STR profiling and were regularly tested to exclude mycoplasma contamination.

### Plasmids, Ientiviral Production, and Cell Line Generation

LINC01235 full‐length cDNA were synthesized (Genewiz) according to 5′ and 3′ RACE results and cloned into pCDH‐CMV‐Puro (SBI, CD510B‐1) vector, pLVX‐TetOne‐puro vector (Takara, 631 847). For clonal competition assay, the puro cassette in the pCDH‐CMV‐Puro vector was replaced with eGFP or mCherry. For the in vivo clonal competition assay, the puro cassette in pLVX‐TetOne‐Puro was replaced with an eGFP sequence. The full sequences of isoforms are listed in File S1 (Supporting Information). Two single guide RNAs (sgRNA) targeting LINC01235 were designed by CRISPR‐ERA^[^ ^40^ ^]^ and cloned into PX459 (addgene, 62 988) vectors. PIP‐FUCCI plasmid (addgene, 138 715) was obtained from Addgene.

Primers for RT‐qPCR were synthesized by Sangon Biotech. ASOs were synthesized with phosphorothioate backbone+ 5′ cholesterol modification + 2 nt lock nucleic acids at each end. ChIRP primers were synthesized with 3′ Biotin‐TEG. ASOs, siRNAs, and ChIRP probes were synthesized by Hippo Bio.

To generate LINC01235 knockdown cells, PX459‐sgRNAs were transfected by Lipofectamine 3000 (Invitrogen, L3000001) followed by puromycin selection for 3 days (Invivogen, ant‐pr‐1, 2 µg ml^−1^) in HCC1954 cells. Scramble sgRNAs were used to construct control cells. Clones were selected and verified by RT‐qPCR.

RNA interference experiments were carried out by transfection of the indicated antisense oligonucleotides (ASOs) or siRNAs using RNAiMAX (Thermo, 13 778 075) according to the manufacturer's instructions. After 72 h, cells were collected for further study. The target sequences are listed in File S2 (Supporting Information).

To generate recombinant lentiviruses, pCDH and pLVX vectors containing LINC01235 sequence or not were transfected with helper plasmids psPAX2 (Addgene, 12 260) and pMD2.G (Addgene, 12 259) into 293FT (Invitrogen, R70007) cells as previously described. After 72 h, the virus was harvested by passing through a 0.45 mm filter. The collected lentivirus was used directly to infect cells with the addition of 5 mg ml^−1^ polybrene (Sigma, H9268) or stored in −80 °C. Infected cells were selected with puromycin at 2 µg ml^−1^. Experiments with infected cells were performed 3 weeks after selection.

For doxycycline inducement expression of LINC1235 in vitro, 1 µg ml^−1^ doxycycline (A426819, Sangon) was added to culture media 72 h before assays.

### RNA Isolation, RT‐qPCR, and RACE

Total RNAs from cultured cells were extracted with TRIzol (Invitrogen, 15 596 026) according to the manufacturer's protocol. Clinical samples were stored in RNAlater (Invitrogen, AM7024) and extracted with TRIzol with the facilitation of a rotor‐stator homogenizer. cDNAs were reverse transcribed with Hiscript III Reverse Transcriptase (Vazyme, R312) with oligo (dT) and random hexamers. Real‐time quantitative PCR was performed with ChamQ SYBR qPCR Master Mix (Q311, Vazyme) and QuantStudio 7 (Applied Biosystems, 4 485 701). The relative expression of different sets of genes was quantified to GAPDH. Primer sequences for RT‐qPCR and RT‐PCR used are listed in File S2 (Supporting Information).

The 3′ and 5′ Rapid Amplification of cDNA Ends (RACE) was performed using the RLM‐RACE kit (Invitrogen, AM1700) following the manufacturer's instructions. RNA was extracted from HCC1954 cells. Primers used for 3′ and 5′ RACE are listed in File S2 (Supporting Information).

### Protein Extracts and Western Blot Analysis

Cells were seeded in 6‐multiwell tissue culture plates and grown for two to three days until culture plates reached 80–90% confluency. For aphidicolin (APH, Sigma, A4487) or hydroxyurea (HU, Sigma, H8627) treatment, 1 µm APH or 10 mm HU was added for the indicated time. Cells were lysed and homogenized by RIPA (Thermo, 89 901) containing Protease Inhibitor cocktail (Selleck, B14001) and phosphatase inhibitor cocktail (Selleck, B15001), and BCA Protein Assay kit (Thermo, 23 225) was used for protein quantification. Cell lysates were separated by 10% SDS–PAGE or 4–20% Bis‐tris Precast Gel (Tanon, 180–9110H). Fractionated proteins were transferred to PVDF membranes (Millipore, ISEQ00010). After blocking with TBS, 0.1% Tween‐20, and 5% skim milk (Sangon, A600669), the membranes were probed with antibodies signals were enhanced by secondary antibodies (HRP‐anti‐mouse, 111‐035‐144, Jackson; HRP‐anti‐rabbit, 111‐035‐046, Jackson). Chemiluminescent detection (180‐5001, Tanon) was used. Primary antibodies and dilutions are listed below: phospho‐MCM2 (Cell Signaling, 12 958, 1:1000), MCM2 (Cell Signaling, 3619, 1:1000), MCM3 (Cell Signaling, 4012, 1:1000), MCM7 (Cell Signaling, 4018, 1:1000), CDC45 (Cell Signaling, 11 881, 1:1000), CDC6 (Cell Signaling, 3387, 1:1000), PCNA (Cell Signaling, 13 110, 1:1000), Phospho‐CDC2 (Cell Signaling, 4539, 1:1000), phospho‐RB (Cell Signaling, 8516, 1:1000), GAPDH (Proteintech, HRP‐60004, 1:10 000), 53BP1(Cell Signaling, 88 439, 1:1000), γ‐H2AX (Cell Signaling, 9718, 1:1000), RPA32 (Cell Signaling, 10 148, 1:1000), XRCC5 (Cell Signaling, 2180, 1:1000), XRCC6 (Cell Signaling, 4588, 1:1000), phospho‐DNA‐PKcs (Cell Signaling, 68 716, 1:1000), DNA‐PKcs (Cell Signaling, 36 168, 1:1000), p‐Chk1 (Cell Signaling, 12 302, 1:1000), RPA32 (Cell Signaling, 35 869, 1:1000), and phospho‐ATM/ATR substrate (Cell Signaling, 2851, 1:1000). Signals were detected and processed using ChemiDoc (Bio‐Rad).

### Cell Proliferation and Growth Inhibition Assay

For proliferation assay, cells were seeded in 96‐well flat‐bottomed plates with each well containing 3000 cells in 200 µl of culture medium and cultured in the ambient environment described above. Cells were assayed with Cell Counting Kit 8 (Dojindo, CK04) every 24 h. OD values were used for plotting.

For the clonal formation assay, cells were seeded in 6 well or 12 well plates. Indicated treatments were given after 24 h. Plates were stained with 1% crystal violet.

For growth inhibition assay, cells were plated in each well of 96 well plates and treated with the indicated agents 24 h later at the concentrations shown. After 5 days, growth inhibition was measured using the Cell Counting Kit 8 according to the manufacturer's instructions. Plates were read at 450 nm absorbance. Technical replicates were performed 3 times for each condition, and biological replicates were performed 2–3 times per experiment. Lapatinib (S2111), pyrotinib (S8852), neratinib (S2150), paclitaxel (S1150), trastuzumab (A2007), T‐DXd (E0200), AZD6738 (S7693) and olaparib(S1060) were obtained from Selleck Chem.

### Immunohistochemistry

Tissue or tumors were fixed in 4% paraformaldehyde overnight. Dehydration and embedding in paraffin were performed following routine methods. These paraffin blocks were cut into 5 mm slides and adhered on the slides glass. Paraffin sections were deparaffinized in xylene and then rehydrated in 100, 95, and 75% alcohol successively. Antigen was retrieved by critic acid buffer (pH 6.0) in the pressure cooker for 15 min. Endogenous peroxidase was inactivated by incubation in 3% H_2_O_2_ for 15 min. Following preincubation with 10% normal goat serum to block nonspecific sites for 30 min, the sections were incubated with anti‐GFP (1:200, 2555, CST) in a humidified chamber at 4 °C overnight. After the sections were washed with PBS three times, HRP‐conjugated secondary antibodies (anti‐rabbit, 111‐035‐046, Jackson) were applied. After washing 3 times with PBS, staining was performed with DAB kit (Vector, SK‐4105) before counterstaining with hematoxylin.

### RNA Fluorescence In Situ Hybridization (FISH) and Subcellular Location

LINC01235 RNA FISH was performed using the Ribo FISH Kit (Ribobio, C10910) according to the manufacturer's protocol. FISH probes were synthesized by Ribobio (18S, lnc110102; LINC01235, lnc1101283). Briefly, cells were grown on coverslips in a 24‐well culture plate. Cells were fixed with 4% (w/v) paraformaldehyde in 1 × PBS for 10 min. Fixed cells were permeabilized for 5 min at 4 °C. The coverslips were washed three times with PBS for 5 min at room temperature and then blocked with pre‐hybridization Buffer at 37 °C for 30 min. Cy3 labeled‐LINC01235 or 18S RNA probes were hybridized at 37 °C overnight. The coverslips were then washed three times with Wash Buffer I, once with Wash Buffer II, and once with Wash Buffer III. Cover slides were stained with DAPI and then mounted. Images were acquired with a Leica confocal microscope.

For nuclear and cytoplasmic RNA separation, 1 × 10^6^ cells were collected and extracted using a PARIS kit according to the manufacturer's instructions (AM1921, Invitrogen). RNAs were reverse transcribed and analyzed with quantitative PCR. The proportion of genes expressed in nuclear and cytoplasmic fractions was calculated.

### Clonal Competition Assay and PIP‐FUCCI Assay

Cells infected with GFP‐LINC01235 or mCherry‐EV were seeded in 24 well plates at a ratio of 1:1. After 24 h, cells were placed into Cytation 5. GFP and mCherry channels were selected for imaging with an interval of 6 h. For PIP‐FUCCI, cells were seeded in 6 well plates 24 h before imaging. Cells were imaged with an interval of 30 min. The G1 phase was determined as the interval between the first appearance cell division and the disappearance of green fluorescence. At least 100 cells were analyzed for each experiment.

### RNA Pull‐Down Assay and Mass Spectrometry

RNA pull‐down was performed as previously described.^[^ ^41^ ^]^ Briefly, LINC01235 isoforms were in vitro transcribed (E2040S, NEB) and labeled with Biotin‐16‐UTP (11 388 908 910, Roche) following the manufacturer's instruction. HCC1954 cells were used for RNA pull‐down/MS analysis. Cell lysates were prepared with modified RIPA (50 mm Tris‐HCl pH 7.4, 150 mm NaCl, 1% Igepal 630, 0.5% sodium deoxycholate). For each sample, 3 µg RNA was mixed with 1 × 10^7^ cell extract and incubated at 4 °C for 4 hours, followed by incubating with 50 µl Dynabeads C1 (Invitrogen, 65 002) at 4 °C for 1 h. Beads were washed 6 times with modified RIPA. Proteins were eluted with 1× protein loading buffer at 100 °C for 10 min. After elution, the samples were detected by immunoblot.

For MS, beads were washed 3 times with PBS after modified RIPA wash. Beads were snapped frozen and sent for LC‐MS/MS with TMT‐Label (Shanghai Applied Protein Technology, Shanghai, China). Briefly, beads were washed and re‐suspended in a wash buffer (200 mm EPPS, pH 8.5). Samples were digested overnight with Trypsin at 37 °C for 6 h. Supernatant from each sample was collected in a separate tube. Beads were washed with wash buffer and combined with wash buffer. Samples were de‐salted using a Sep‐pak. Digested peptides were labeled with TMT 10‐plex reagents. Then, 5 µL of each sample was pooled and used to shoot a ratio check in order to confirm complete TMT labeling. All samples were pooled into a single sample. The pooled peptides were desalted by stage‐tip.

Peptides were separated within 80 min at a flow rate of 400 µl min^−1^ on a reversed‐phase C18 column (15 cm × 75 µm, 3 µm, Eksigent). Mobile phases A and B were with 0.1% formic acid in 2% ACN and 0.1% formic acid in ACN. The fraction of B was linearly increased from 0 to 5% within 5 min, followed by an increase to 40% at the 45th minute and a further increase to 80% at the 46th minute, kept on until the 50 min. On the 50 min, returned to 5% until the 60 min. Flow rate 3 µl min^−1^. Peptides were analyzed on an Orbitrap Exploris 480. MS2 spectra were searched using the PD database. For proteome, Peptide spectral matches were filtered to a 1% false discovery rate (FDR) using the target‐decoy strategy combined with linear discriminant analysis. The proteins were filtered to a <1% FDR.

### RNA Immunoprecipitation Assay

For XRCC5 RIP assay, 1 × 10^7^ HCC1954 cells were harvested, resuspended in 200 µL NT‐2 buffer (50 mm Tris‐HCl pH 7.4, 150 mm NaCl, 1 mm MgCl2, 0.05% NP‐40, 1× Protease Inhibitor, and 200 U ml^−1^ RNaseOUT (Thermo, 10 777 019)), and incubated on ice for 5 min and then stored at −80 °C to promote the cell lysis. Then, 75 µl Dynabeads Protein A and G (Invitrogen, 10001D, 10009D) were added into a 1.5 ml tube and washed twice with 0.5 ml of NT‐2 buffer. The beads were resuspended in NT‐2 buffer and 5 µg of the anti‐XRCC5 antibody (Proteintech, 16389‐1‐AP) or IgG control (Proteintech, 30000‐0‐AP) were added to the tubes. The beads were incubated with rotation for 1 h at room temperature and washed six times with NT‐2 buffer. After the sixth wash, the beads with 900 µl of NT‐2 buffer were supplemented with 20 mm EDTA pH 8.0, 1 mm DTT, and 200 U ml^−1^ RNaseOUT. The lysate was thawed on ice and centrifuged at 20 000 × g for 10 min at 4 °C, and 100 µl of supernatant was added it to each antibody bead reaction. The final volume of the immunoprecipitation reaction was 1 ml. All tubes were incubated on a rotating wheel for 3 h up to overnight at 4 °C. Then, samples were washed 6 times with NT‐2 buffer. Immunoprecipitates were resuspended each in 150 µl of 1× NT‐2 buffer supplemented with 1% SDS and 1.2 mg ml^−1^ Proteinase K (NEB, P8102). All tubes were incubated at 56 °C for 30 min with shaking to digest the proteins. After 30 min of incubation, tubes were spined down and placed in a magnetic rack, and the supernatant was transferred to a new tube where 250 µl of NT‐2 was added. The RNA was purified using an RNA Clean & Concentrator‐5 (Zymoresearch, R1013). For qRT‐PCR, reverse transcription was performed using Hiscript III Reverse Transcriptase (Vazyme) with oligo (dT) and random hexamers followed by qRT‐PCR analysis.

### Chromatin Isolation by RNA Purification

Each probe was designed using LGC Biosearch's web‐based designer, biotin‐labeled at its 3′ terminus. Probes targeted luciferase were used as a negative control. For each sample, 1 × 10^7^ HCC1954 cells were used and crosslinked in 1% glutaraldehyde at room temperature for 10 min and quenched with 125 mm glycine for 5 min. The cells were washed twice with ice‐cold PBS, pelleted, snap‐frozen, and stored at −80 °C. Nextday, the pellets were thawed on ice and lysed with ChIPR lysis buffer at 100 mg pellet per mL buffer with 1× protease inhibitor(selleck). Lysed cells were sonicated by Bioruptor plus with 30 s on and 30 s off for 8 cycles. DNA was sheared to 100–500 bp fragments at 4 °C. Cleared lysates were hybridized with probes in 37 °C in a hybridization oven for 4 h. After washes and elution, RNA and DNA were purified accordingly. qPCR and RT‐qPCR were performed as described above. DNA for library construction was performed as described in the library preparation section. The probes used in the ChIRP assay are listed in File S2 (Supporting Information).

### ChIP Assay

For the ChIP assay, HCC1954 cells were transfected with ASOs as indicated. After 72 h, cells were treated with 1% formaldehyde for 10 min at RT prior to the addition of glycine at the final concentration of 0.125 m for 5 min. Cells were washed twice with PBS and treated with Buffer A (10 mm Tris‐HCl 7.5, 10 mm NaCl, and 0.5% NP‐40) with 1× Protease Inhibitor Cocktail (PIC) for 10 min on ice. Washed once with Buffer A+PIC, Cells were resuspended in nuclei lysis buffer (50 mm Tris‐HCl pH 8.0, 10 mm EDTA pH 8.0, and 1% SDS). The nuclei were sonicated for several rounds until the de‐crosslinked DNA fragment was ≈200–500 bp with Bioruptor Pico (5 cycles). Centrifuge 12 000 g at 10 °C for 15 min. For 100 µl cleared sonicated chromatin, added 900 µl ChIP dilution buffer (0.01% SDS, 1% Triton‐X100, 1 mm EDTA pH 8.0, 16.7 mm Tris‐HCl pH 8.0, and 167 mm NaCl) with PIC. Then, 30 µg sonicated chromatin was incubated 7.5 µl of H3K27ac (Cell Signaling, 8173), H3K9ac (Cell Signaling, 9649), H3K36me3 (Cell Signaling, 9733) antibody or 5 µg H2A.Z (Abcam, ab4174) antibody with rotation at 4 °C overnight. For each reaction, mixed 25 µl Protein A and 25 µl Protein G dynabeads at a 1:1 ratio and blocked with 1% BSA in ChIP dilution buffer overnight. Beads were washed 3 times with ChIP dilution buffer before added into the lysates. Incubated at 4 °C for 4 h with rotation. The beads were washed twice with RIPA‐150 (50 mm Tris‐HCl, pH 8.0, 157 150 mm NaCl, 1 mm EDTA, pH 8.0, 0.1% SDS, 1% Triton X‐100, and 0.1% sodium deoxycholate), then twice with RIPA‐500 (50 mm Tris‐HCl, pH 8.0, 500 mm NaCl, 1 mm EDTA, pH 8.0, 0.1% SDS, 1% Triton X‐100, and 0.1% sodium deoxycholate). The beads were further washed twice with RIPA LiCl (50 mm Tris‐HCl, pH 8.0, 1 mm EDTA, pH 8.0, 1% NP‐40, 0.7% sodium deoxycholate, and 500 mm LiCl). The beads were washed twice with TE and resuspended with 150 µl freshly made elution buffer (0.1 m NaHCO_3_, 1% SDS) with 5 µl Proteinase K and 2 µl RNase A (Thermo, R1253). Incubated at 56 °C overnight shaked at 900 rpm. DNA was purified with PCR and DNA Cleanup Kit (NEB, T1030) following instructions. The immunoprecipitated DNA samples were then quantified by RT‐qPCR or for library preparation. The DNA library was prepared using a Next Ultra DNA Library Prep Kit (NEB, E7645) according to the manufacturer's instructions. The libraries were purified with AMPure XP beads (Beckman, A63881) according to the manufacturer's instructions, and sequenced using Illumina platforms. DNA oligo sequences used in this study are available in File S2 (Supporting Information).

### ATAC‐Seq

For ATAC‐seq, 1 × 10^5^ HCC1954 cells treated with indicated ASOs were harvested and counted. Washed twice with PBS, cells were lysed in Lysis buffer (10 mm Tris‐HCl pH 7.5, 10 mm NaCl, 3 mm MgCl_2_, 0.1% NP‐40, 0,1% Tween‐20, and 0.01% Digitonin) on ice for 5 min then centrifuge for 10 min at 500 g. Pellets were resuspended in 50 µl 1× TD Buffer (Illumina, 15 027 865) with 7.5 µl Tn5 Transposase (Illumina, 15 027 865), 0.01% Digitonin, 0.1% Tween and incubated at 37 °C for 30 min. DNA was purified by Monarch PCR & DNA Cleanup Kit (NEB, T1030). Eluted DNA was amplified with NEBNext High‐Fidelity 2X PCR Master Mix (NEB, M0541L) and TruePrep Index Kit V2 for Illumina (Vazyme, TD202) for 10 cycles and purified by AMPure XP beads (0.5*x*−0.7*x*).

### Neutral Comet Assay

Cells were treated at indicated conditions before collection. Comet assays were performed using the CometAssay kit (Trevigen, 4250‐050‐ESK) per the manufacturer's instructions. Briefly, cells were washed once in ice‐cold PBS, resuspended to 1 × 10^5^ cells mL^−1^ in PBS, mixed 1:10 (v/v) with molten low‐melt agarose, spread onto Comet slides, allowed to set at 4 °C for 30 min, and lysed in Lysis Solution overnight. Slides were immersed in Neutral Electrophoresis Buffer for 30 min, subjected to electrophoresis in the same buffer at 21 V for 45 min at 4 °C, and immersed in DNA Precipitation Solution for 30 min at RT. Slides were immersed for 30 min in 70% ethanol then dried at 37 for 15 min. Dried slides were stained with a 1:30 000 dilution of SYBR‐GOLD (Invitrogen, S11494) and visualized and analyzed. To assess the amount of DNA damage, computer‐generated tail moment values by CometScore Software Version 2.0 were used. A minimum of 100 cells were analyzed per experimental point. Apoptotic cells (small comet head and very large comet tail) were excluded from the analysis.

### Cell Cycle Synchronization

For synchronization to G1/S, the cells were treated with 2 mm of thymidine for 18 h following washing cells with PBS, and a fresh medium was added. Following 9 h of incubation of the cells, 2 mm thymidine was added and the cells were cultured for an additional 18 h to synchronize cells at G1/S boundary. Cells were washed with PBS, replaced with fresh media following collection at the indicated time for cell cycle analysis by flow cytometry. EdU (10 µm, Thermo, A10044) was added 30 min before collection.

### Flow Cytometry

For cell cycle analysis and chromatin‐bound protein analysis, growing cells or treated cells were added with EdU (10 µm, Thermo, A10044) for 30 min before collection. Cells were collected by trypsinization. After centrifugation (500 × g at 4 °C for 5 min), harvested cells were washed with FBS buffer (PBS+1% FBS) twice, and fixed in 4% paraformaldehyde for 10 min at room temperature. For chromatin‐bound protein analysis, harvested cells were washed with FBS buffer twice and lysed in cold CSK buffer (10 mm HEPES, pH 7.0, 300 mm sucrose, 100 mm NaCl, 3 mm MgCl2, 1 mm EGTA, 1% BSA, 0.2% Triton X‐100, 1 mm DTT, and 1× protease inhibitors and phosphatase inhibitors) for 10 min on ice, then washed twice with FACS buffer (PBS+1% FBS) before fixed in 4% paraformaldehyde for 15 min at room temperature. Fixed cells were washed twice with FACS buffer and permeabilized in Perm buffer (FACS buffer + 0.1% Saponin) at 4 °C for 15 min. After washing once with Perm buffer, cells were resuspended with 500 µl Click‐it reaction mix added (439 µl PBS, 10 µl CuSO4, 50 µl 1 m sodium ascorbate, 1 µl 0.5 mm Alexa Fluor 647 Azide (Thermo, A10277)) and incubated for 30 min at room temperature. Cells were washed twice with Perm buffer. For chromatin‐bound protein staining, cells were blocked with 10% goat serum and incubated with primary antibodies overnight. Washed twice and incubated with secondary antibodies for 1 h at room temperature. For all the experiments that need cell cycle analysis, cells were washed twice with Perm buffer and stained with propidium iodide (Thermo, P3566) + RNase A at room temperature for 15 min. After washing twice with FACS buffer, the samples were analyzed on CytoFLEX Flow Cytometer, and data analysis was conducted with the FlowJo software. Phospho‐MCM2 (Ser139) (Cell Signaling, 12 958, 1:200), γ‐H2AX (Cell Signaling, 9718, 1:400), 53BP1 (ab175933, 9718, 1:200), Rabbit IgG Isotype Control (Cell Signaling, 3900, 1:200), Goat Anti‐Rabbit IgG Alexa Fluor 488 (abcam, ab150077, 1:2000).

DNA reporter cells were constructed by infection cells with pLCN DSB Repair Reporter (addgene, 98 895) with G418 selection for one month until killing control was all dead. For the NHEJ DNA repair assay, one million cells containing integrated reporter were transfected with 2.5 µg of pCBASceI plasmid (addgene, 26 477). For HR DNA repair reporter analysis, one million cells containing integrated reporter were transfected with 4 µg of pCAGGS‐DRR‐mCherry plasmid (addgene, 98 896) and 2.5 µg of pCBASceI plasmid (addgene, 26 477). Forty‐eight hours after transfection, cells were trypsinized and resuspended in FBS buffer and directly subjected to flow cytometry analysis.

### Immunofluorescence

Cells were plated the day before the experiment in 12 well plates with round cover glass (VWR, 48380‐046). Treatments were given as indicated in the figures.

For micronuclei analysis, cells were fixed with 4% paraformaldehyde for 10 min at room temperature. Washed twice with PBS, slides were counterstained with DAPI (0.1 µg ml^−1^, Thermo, 62 248), rinsed with PBS, and sealed with VECTASHIELD Vibrance (Vector, H‐1700).

For chromatin‐bound protein identification, slides were immersed in ice‐cold CSK buffer for 10 min on ice and washed twice with PBS, then fixed in 4% paraformaldehyde for 15 min at room temperature. The slides were washed 2 times with PBS+0.1% Tween 20 (PBST). Then, slides were immersed in Perm buffer for 10 min at 4 °C. Blocked with 10% goat serum and 0.1% Triton X‐100 in PBS for 1 h at 4 °C. Primary antibodies against 53BP1(Cell Signaling, 4937, 1:100) or γ‐H2AX (Cell Signaling, 9718, 1:800) were incubated at 4 °C in a humid chamber overnight, followed by three PBST washes and subsequent incubation with secondary antibodies for 1 hour at room temperature. Slides were washed in PBS three times and counterstained with DAPI (0.1 µg ml^−1^), rinsed with PBS, and mounted with VECTASHIELD Vibrance. The slides were imaged using a Leica STELLARIS 8 confocal microscope and analyzed using LAS X or ImageJ.

### Library Preparation and Deep Sequencing

The total RNA samples were treated with VAHTS mRNA Capture Beads (Vazyme) to enrich polyA+ RNA before constructing the RNA‐seq libraries. RNA‐seq libraries were prepared using VAHTS mRNA‐seq v2 Library Prep Kit for Illumina (Vazyme) following the manufacturer's instructions. Briefly, polyA+ RNA samples were fragmented and then used for first‐ and second‐strand cDNA synthesis with random hexamer primers. The cDNA fragments were treated with a DNA End Repair Kit to repair the ends, then modified with Klenow to add an A at the 3′ end of the DNA fragments, and finally ligated to adapters. Purified dsDNA was subjected to 12 cycles of PCR amplification, and the libraries were sequenced by Illumina sequencing platform on a 150 bp paired‐end run. Sequencing reads from RNA‐seq data were aligned using the spliced read aligner HISAT2, which was supplied with the Ensembl human genome assembly (Genome Reference Consortium GRCh38) as the reference genome. Gene expression levels were calculated by the FPKM (fragments per kilobase of transcript per million mapped reads).

The WGS library preparation was carried out by Shbio Company. WGS was performed achieving 20–30× coverage of the human genome with paired‐end sequencing (2 × 150 and 2 × 100 bp). Quality control was performed with fastqc software^[^ ^42^ ^]^ and alignment to the human genome (GRCh38/hg38 version) was performed with the bowtie2 algorithm.^[^ ^43^ ^]^ Samtools^[^ ^44^ ^]^ was used to convert sam files to bam and for sorting bam files. Breakdancer software^[^ ^45^ ^]^ (breakdancer‐1.12011_02_21 version) was utilized to identify SV (intra‐ and inter‐chromosomal translocations, deletions, insertions and inversions).

ChIP and ChIRP DNA libraries were prepared using the NEBNext DNA Library Prep Kit for Illumina (New England Biolabs) according to the manufacturer's instructions. The raw reads were aligned by Bowtie2 (v2.2.9) with a human reference genome (hg38). The uniquely mapped reads were subjected to the peak calling algorithm, MACS (v1.4.2) with default parameters.

### Biological Materials

There were no restrictions on the availability of unique biological materials except human samples upon reasonable request to the corresponding author. The request of patient samples was available after approval by following The Approval for International Cooperation in Scientific Research on Human Genetic Resources in China.

### Statistics and Reproducibility

Statistical analyses were performed as described in the figure legend for each experiment. Statistical analysis was performed using GraphPad Prism software (v10.0) unless otherwise specified. All measurements were taken from distinct samples. All data were presented as mean ± s.e.m. unless otherwise noted in the legends. Differences were considered statistically significant at *P <* 0.05. In all cases: ns not significant; * *p* < 0.05; ** *p* < 0.01; *** *p* < 0.001.

### Analysis of lncRNA Coding Ability and Species

CPC, CPAT, and phyloCSF were used for RNA coding ability assessment as described previously.^[^ ^20^, ^21^, ^22^, ^34^ ^]^ The full length of LINC01235 was used for prediction. PLAR database was used for conservation assessment between species as described previously.^[^ ^23^, ^34^ ^]^ Hg38 was used for the reference genome.

### Analysis of WGS Data

The WGS library preparation was carried out by Shbio Company. WGS was performed achieving 20–30× coverage of the human genome with paired‐end sequencing (2 × 150 and 2 × 100 bp). Quality control was performed with fastqc software^[^ ^42^ ^]^ and alignment to the human genome (GRCh38/hg38 version) was performed with the bwa algorithm.^[^ ^43^ ^]^ Samtools^[^ ^44^ ^]^ was used to convert sam files to bam and for sorting bam files. Local realignment, duplicate removal, and base quality adjustment were conducted by the Genome Analysis Toolkit (GATK,v4.1.7).^[^ ^46^ ^]^ Mutect2(v4.1.7) was used to call Somatic single‐nucleotide variants (SNVs). CNV was detected by CNVkit^[^ ^47^ ^]^ (v0.9.11) and further analysis and visualization were performed by GISTIC (v 2.0.22) and ggplot2 (v3.4.2). Breakdancer software^[^ ^45^ ^]^ (breakdancer‐1.12011_02_21 version) was utilized to identify SV (intra‐ and inter‐chromosomal translocations, deletions, insertions and inversions).

### Analysis of ChIP‐Seq, ChIRP‐Seq and ATAC‐Seq Data

The ChiLin pipeline 2.0.052 was used for quality control and pre‐processing of the data. Bowtie2 (v2.2.9) was used as a read mapping tool to align to hg38, and Model‐based Analysis of ChIP‐Seq (MACS2)(v2.1.0.20140616) as a peak caller using the command line parameters “‐SPMR ‐B ‐q 0.01 –keep‐dup 1 –nomodel ‐hs ”. The read counts of these peaks were obtained using HTseq‐count (v2.0.3).^[^ ^48^ ^]^ The differential analyses between the NC group and the Si‐1235 group were performed using peak counts for ChIP‐seq following the DEseq2 (v1.22.2) pipeline with a False Discovery Rate threshold of 0.05 and |log2FC| > 1. And BEDTools (v2.31.1)^[^ ^49^ ^]^ was used to extract interested peaks for further analysis. ATAC peaks were called using MACS2 with an FDR < 0.01 cut‐off. After calling peaks using MACS2 to generate a bedgraph file of these peaks, it was converted to a bigWig file using the bedGraphToBigWig tool. Cistrome Toolkit (dbtoolkit.cistrome.org) was used to probe which factors might regulate the user‐defined genes.

### Visualization of ChIP‐Seq, ChIRP‐Seq, and ATAC‐Seq Data

Normalized profiles corresponding to read coverage per 1 million reads were used for heatmaps and for visualization using the integrative genomics viewer (IGV) and BioSeqUtils (https://github.com/junjunlab/BioSeqUtils). Wiggle tracks were visualized using the integrative genomics viewer. Heat maps were prepared using deepTools (v2.5.4).^[^ ^50^ ^]^

### Analysis and Visualization of RNA‐Seq Data

RNA‐seq reads were mapped by STAR (v2.7.11a)^[^ ^51^ ^]^ to hg38, and gene counts were generated through featureCounts (v2.0.6). Differential gene expression analyses were performed on absolute gene counts for RNA‐seq data using DESeq2 (v1.22.2)^[^ ^52^ ^]^ in R. For GSEA, the ranked gene list was analyzed with the GSEA Preranked v4.3.2 tool using MSigDB Hallmarks v2023.2, C2 v2023.2, C3 gene set collections. Mountain plots were created using the Broad Institute GSEA software.^[^ ^53^ ^]^ To generate bigWig files of RNAseq data, the bamCoverage function from the deepTools (v2.5.4) package with default parameters was used to process the BAM files.

### Statistical Analysis

Statistical analysis was performed using GraphPad Prism software (v10.0) unless otherwise specified. Statistical analysis details for the different experiments are reported in figure legends or the methods section. In all cases: ns not significant; * *p* < 0.05; ** *p* < 0.01; *** *p *< 0.001.

### Software

RStudio (v3.6), QuantStudio, FlowJo (v10.7), BioTek Gen5 Software, LASX Office 1.4.6 28 433, ImageJ v1.52, Image Lab, QuantStudio 7 software and GraphPad Prism (v10.0) were utilized in this study.

## Conflict of Interest

The authors declare no conflict of interest.

## Author Contributions

Q.Z., X.W., and Z.S. contributed equally to this work. Q.Z., X.W., and Z.S. designed and performed most of the experiments and analyzed the data. Q.Z. and B.X. wrote the manuscript with the help of all co‐authors. L.Z. and Y.Z. performed the mouse tumor growth experiments. M.C. and W.C. did the cell culture. H.Z. and X.L. performed the flow cytometry. Z.S. and Y.Z. performed the NGS data analysis. L.L. and P.L. analyzed the clinical data. J.X. collected and prepared the clinical samples for RNA‐seq. X.Z. performed the organoid culture. S.H. and Z.‐M.S. assisted in this study. B.X., W.J., and Y.C. initiated the study, provided funding, and supervised the study.

## Supporting information



Supporting Information

## Data Availability

The data that support the findings of this study are available from the corresponding author upon reasonable request.
