# Supplementary material for: LINC01235 Promotes Clonal Evolution through DNA Replication Licensing‐Induced Chromosomal Instability in Breast Cancer
Source: Adv Sci (Weinh). 2025 Feb 14;12(14):2413527. doi: 10.1002/advs.202413527 (PMC11984920; doi:10.1002/advs.202413527)

ADVANCED SCIENCE
Open Access

# Supporting Information

for *Adv. Sci.*, DOI 10.1002/advs.202413527

LINC01235 Promotes Clonal Evolution through DNA Replication Licensing-Induced Chromosomal Instability in Breast Cancer

*Qi Zhang, Xuliren Wang, Zhibo Shao, Yi Zhang, Liyi Zhang, Ming Chen, Xujie Zhou, Han Zhu, Yue Zhou, Xinya Lu, Pei Li, Weiru Chi, Lun Li, Zhi-Ming Shao, Shenglin Huang, Jingyan Xue, Yayun Chi*, Jiong Wu* and Bingqiu Xiu**

Figure S1

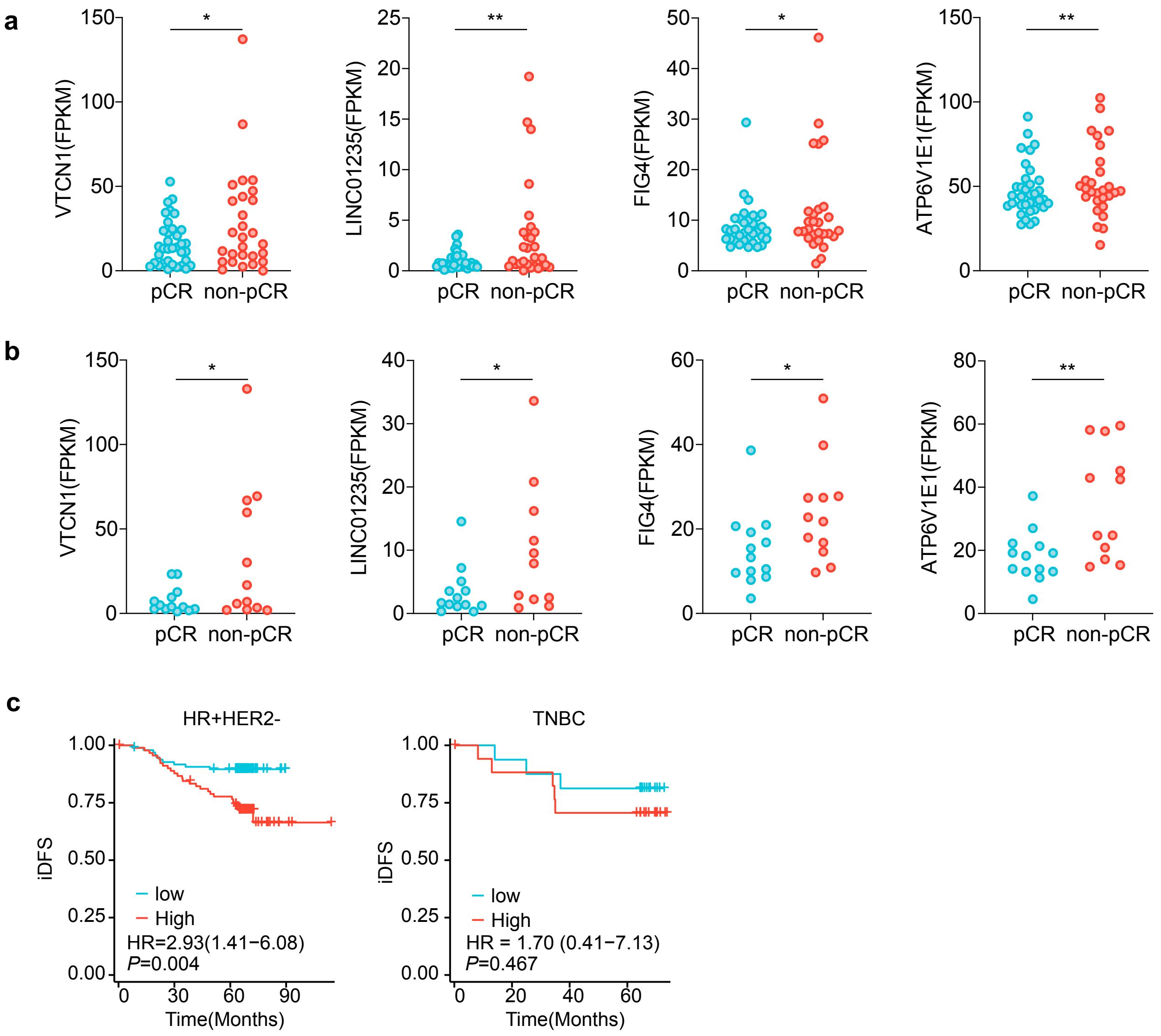


**Figure S1.** **a**, Expression of VTCN1, LINC001235, FIG4, and ATP6VE1 in the FUSCC HER2+ NAC and (**b**) PHEDRA HER2+ NAC cohorts. *P* values were calculated using DESeq2. **c**, Kaplan-Meier curves for invasive disease-free survival (iDFS) of LINC01235-under and -overexpressing patients from the FUSCC early BC cohorts.

Figure S2

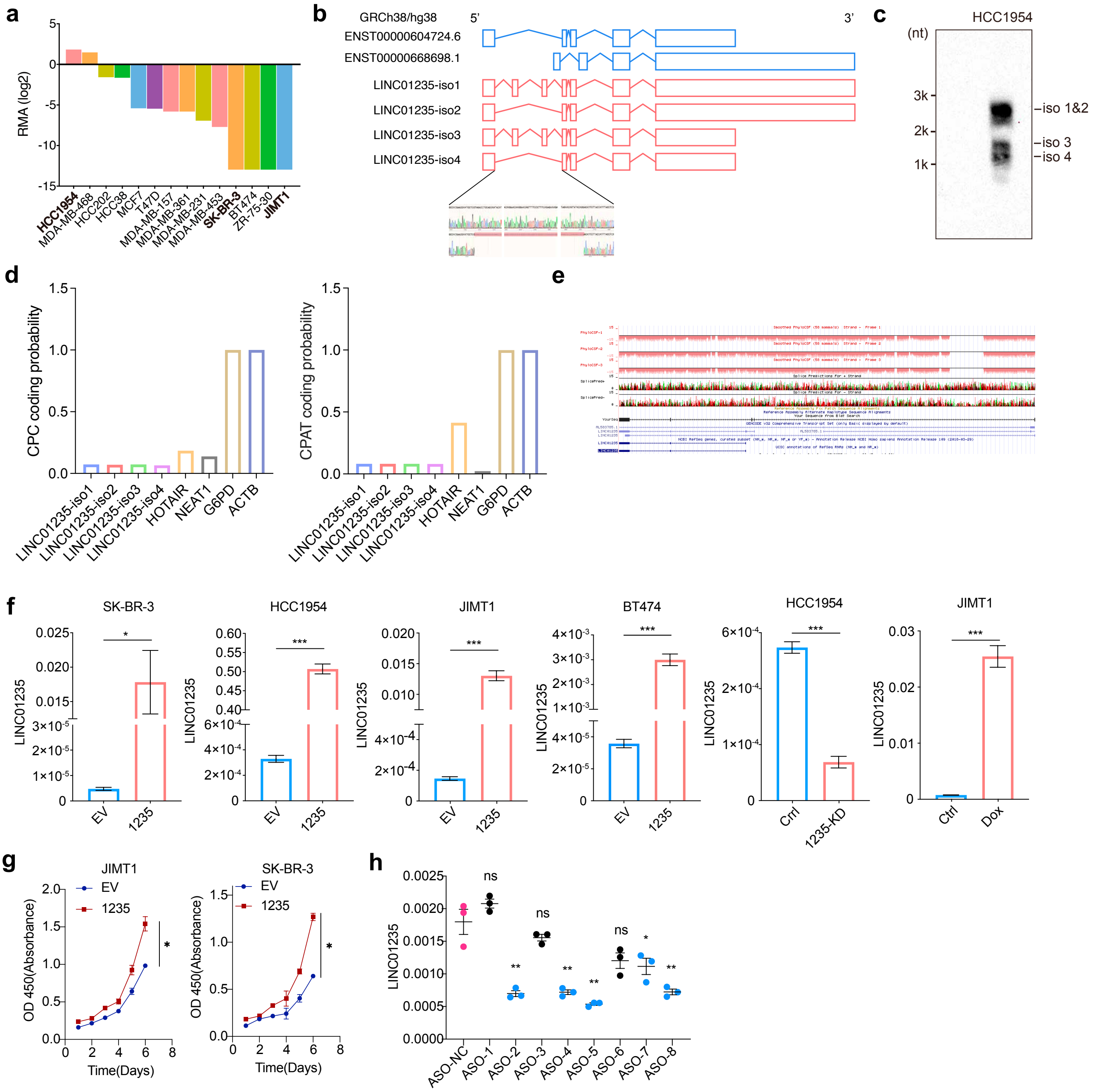

a
RMA (log2)
HCC1954
MDA-MB-468
HCC202
HCC38
MCF7
T47D
MDA-MB-157
MDA-MB-361
MDA-MB-231
MDA-MB-453
SK-BR-3
BT474
ZR-75-30
JIMT1
b
GRCh38/hg38
5'
3'
ENST00000604724.6
ENST00000668698.1
LINC01235-iso1
LINC01235-iso2
LINC01235-iso3
LINC01235-iso4
c
HCC1954
(nt)
3k
2k
1k
iso 1&2
iso 3
iso 4
d
CPC coding probability
CPAT coding probability
LINC01235-iso1
LINC01235-iso2
LINC01235-iso3
LINC01235-iso4
HOTAIR
NEAT1
G6PD
ACTB
e
f
SK-BR-3
HCC1954
JIMT1
BT474
HCC1954
JIMT1
LINC01235
EV
1235
Ctrl
1235-KD
Dox
g
JIMT1
SK-BR-3
EV
1235
OD 450(Absorbance)
Time(Days)
h
LINC01235
ns
ASO-NC
ASO-1
ASO-2
ASO-3
ASO-4
ASO-5
ASO-6
ASO-7
ASO-8

i

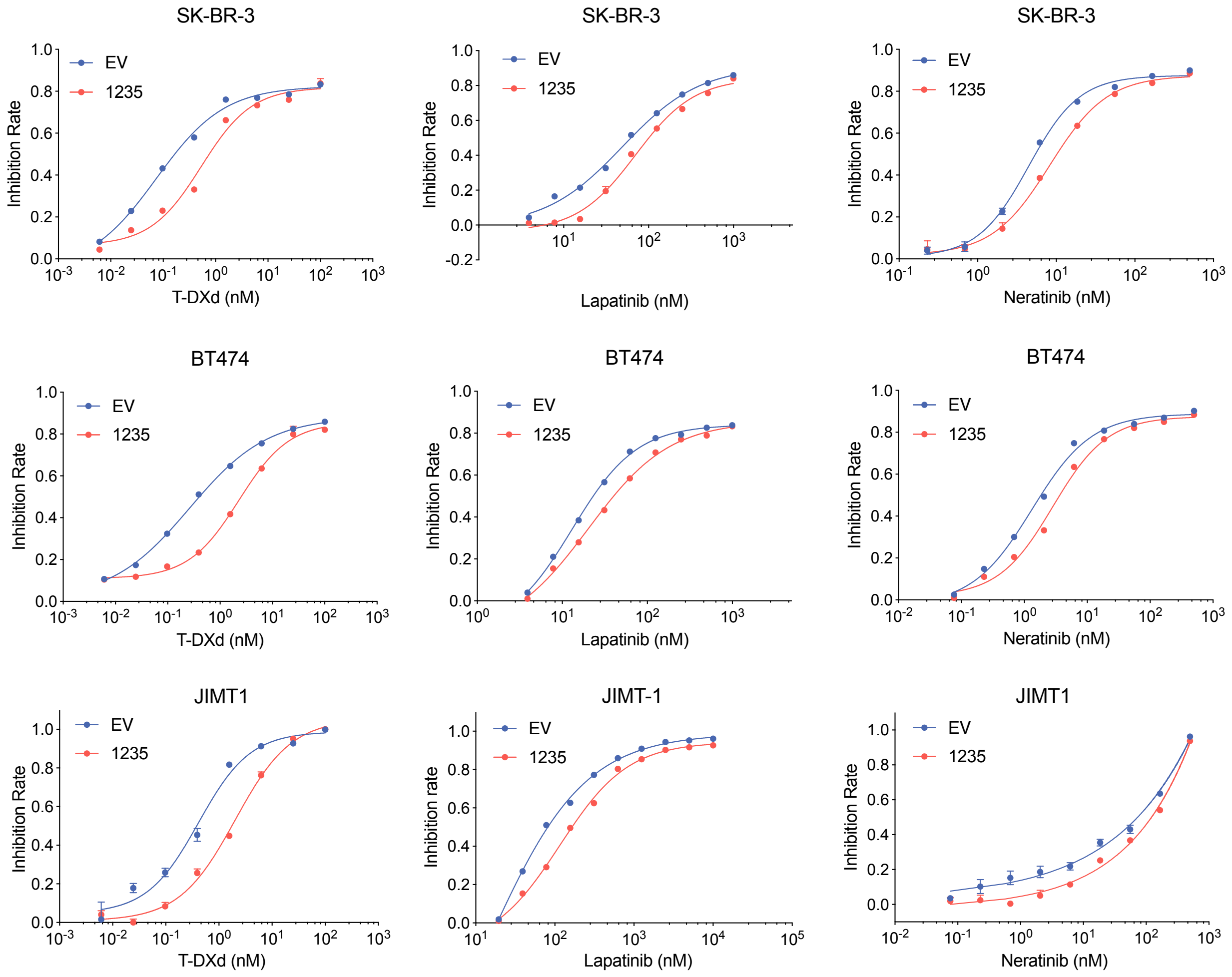
SK-BR-3
EV
1235
Inhibition Rate
T-DXd (nM)
SK-BR-3
EV
1235
Inhibition Rate
Lapatinib (nM)
SK-BR-3
EV
1235
Inhibition Rate
Neratinib (nM)
BT474
EV
1235
Inhibition Rate
T-DXd (nM)
BT474
EV
1235
Inhibition Rate
Lapatinib (nM)
BT474
EV
1235
Inhibition Rate
Neratinib (nM)
JIMT1
EV
1235
Inhibition Rate
T-DXd (nM)
JIMT-1
EV
1235
Inhibition rate
Lapatinib (nM)
JIMT1
EV
1235
Inhibition Rate
Neratinib (nM)

j

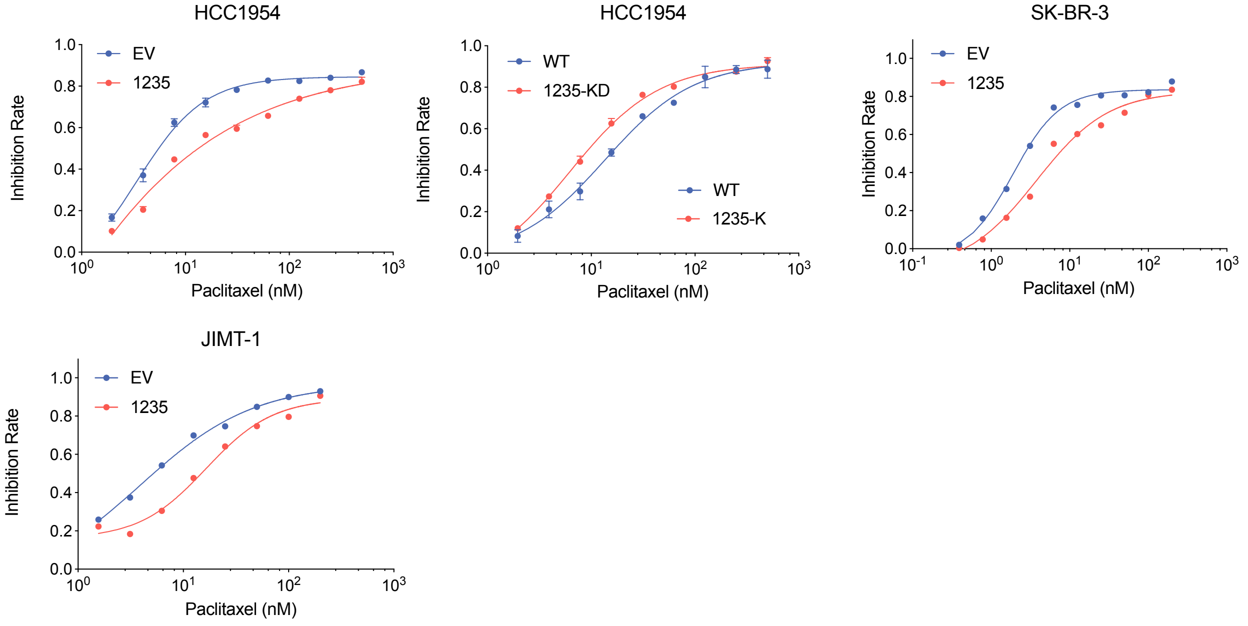
HCC1954
EV
1235
Inhibition Rate
Paclitaxel (nM)
HCC1954
WT
1235-KD
WT
1235-K
Inhibition Rate
Paclitaxel (nM)
SK-BR-3
EV
1235
Inhibition Rate
Paclitaxel (nM)
JIMT-1
EV
1235
Inhibition Rate
Paclitaxel (nM)

**Figure S2. a,** Relative expression of LINC01235 in breast cancer cell lines from the CCLE RNA-seq data. **b**, Schematic view of LINC01235 isoforms and Sanger sequencing data from exons 1 and 4 junctions. Blue: transcripts from the Ensembl database. Red: Transcript isoforms from the RACE results. **c** Northern blot analysis of LINC01235 in HCC1954 cells. **d**, CPC and CPAT coding probabilities of the LINC01235 isoforms are shown. HOTAIR, NEAT1, ACTB, and G6PD were used as negative or positive controls. **e** PhyloCSF coding scores are demonstrated at the LINC01235 region from the UCSC Genome Browser. **f**, Overexpression, knockdown, and DOX-induced strains of LINC01235 in SK-BR-3, BT474, HCC1954, and JIMT1 cells were constructed and verified by RT-qPCR. n=3 biological replicates. Statistical analyses were performed using the two-sided t-test. **g**, Cell proliferation assay of LINC01235 overexpression compared to the empty vector (EV) in JIMT1 and SK-BR-3 cells, n=6 for each time point. Statistical analysis was performed using two-sided t-test at the endpoint. **h**, the ASO efficiency was verified by transfecting HCC1954 cells with 2 nM ASOs. One-way analysis of variance (ANOVA) was performed. n=3 biological replicates. **i**, Growth inhibition assays for neratinib, lapatinib, and T-DXd were performed on SK-BR-3, BT474, and JIMT1 cells. n=3 for each concentration. **j**, Growth inhibition assays of paclitaxel were performed in HCC1954, SK-BR-3, and JIMT1 cells. For **c, f,g** and **i**-**j**, n=2 experiments. * $P<0.05$, ** $P<0.01$, *** $P<0.001$, ns not significant. Error bars represent mean ± SEM (standard error of mean).

# Figure S3

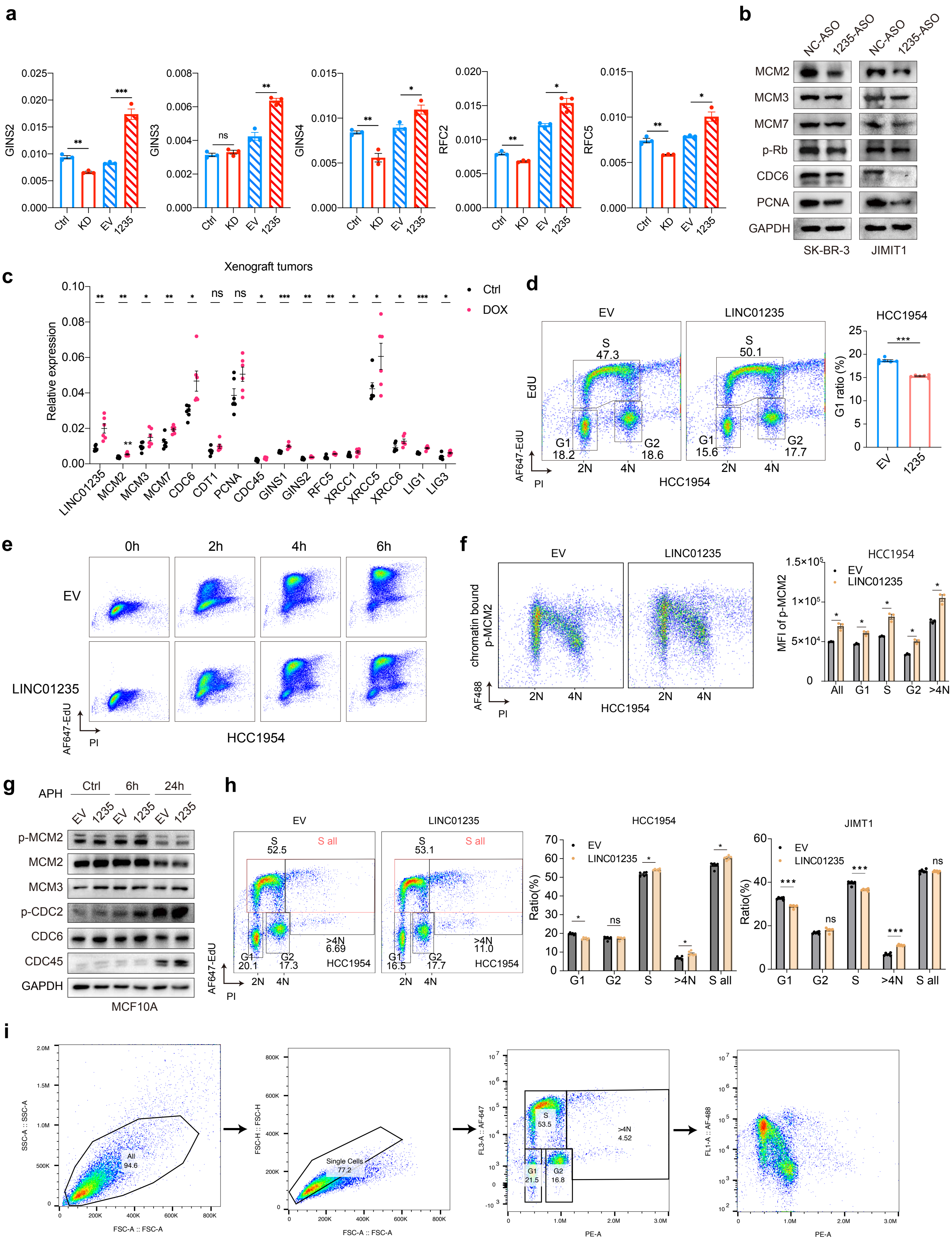
a
GINS2
GINS3
GINS4
RFC2
RFC5
Ctrl
KD
EV
1235
b
NC-ASO
1235-ASO
MCM2
MCM3
MCM7
p-Rb
CDC6
PCNA
GAPDH
SK-BR-3
JIMIT1
c
Xenograft tumors
Relative expression
Ctrl
DOX
LINC01235
MCM2
MCM3
MCM7
CDC6
CDT1
PCNA
CDC45
GINS1
GINS2
RFC5
XRCC1
XRCC5
XRCC6
LIG1
LIG3
d
EV
LINC01235
HCC1954
EdU
AF647-EdU
PI
G1 ratio (%)
e
0h
2h
4h
6h
f
chromatin bound p-MCM2
AF488
MFI of p-MCM2
g
APH
Ctrl
6h
24h
p-MCM2
MCM2
MCM3
p-CDC2
CDC6
CDC45
GAPDH
MCF10A
h
S all
JIMT1
Ratio(%)
i
SSC-A :: SSC-A
FSC-A :: FSC-A
All
94.6
FSC-H :: FSC-H
Single Cells
77.2
FL3-A :: AF-647
PE-A
FL1-A :: AF-488

**Figure S3. a,** RNA levels of DNA replication licensing factors in HCC1954 cells were analyzed by RT-qPCR. Glyceraldehyde-3-phosphate dehydrogenase (GAPDH) was used as a reference gene. LINC01235 knockdown cells (KD) were compared with control cells (Ctrl). LINC01235 overexpression compared to the empty vector (EV). **b** Immunoblot showing DNA replication licensing-related proteins in SK-BR-3 and JIMT1 cells. n=2 experiments. **c** RNA levels of DNA replication licensing factors in JIMT1 DOX-induced xenografts compared with those in non-induced control cells were analyzed by RT-qPCR. GAPDH was used as a reference gene. Samples were obtained from untreated tumors, as shown in Fig. 2k. **d**, Flow cytometric analysis of the cell cycle of HCC1954 cells. The calculation of G1 ratios is presented. n=6 biological replicates. **e**, Flow cytometric analysis of double thymidine blockade and release from HCC1954 cells at the indicated times. The cell cycles were analyzed by staining with propidium iodide and a click reaction with Alexa Fluor 647. **f**, Flow cytometric analysis of chromatin-bound phosphorylated MCM2 and the cell cycle in HCC1954 cells. **g**, Immunoblot showing indicated proteins in MCF10A cells overexpressed with EV and LINC01235 treated with aphidicolin (1 μM) or control for 6 and 24 h. **h**, Representative image of the flow cytometric analysis of the cell cycle in HCC1954 cells overexpressing LINC01235 or EV. n=3 biological replicates. Statistical analyses are presented for HCC1954 and JIMT1 cells.

n=6 for each group. **i**, gating strategy of flow cytometry for cell cycle analysis. For **a**, **d, e**, **g,** and **i**, the statistical analysis was performed using two-sided t-tests. * $P<0.05$, ** $P<0.01$, *** $P<0.001$, ns, not significant. Error bars represent mean ± SEM (Standard error of mean).

# Figure S4

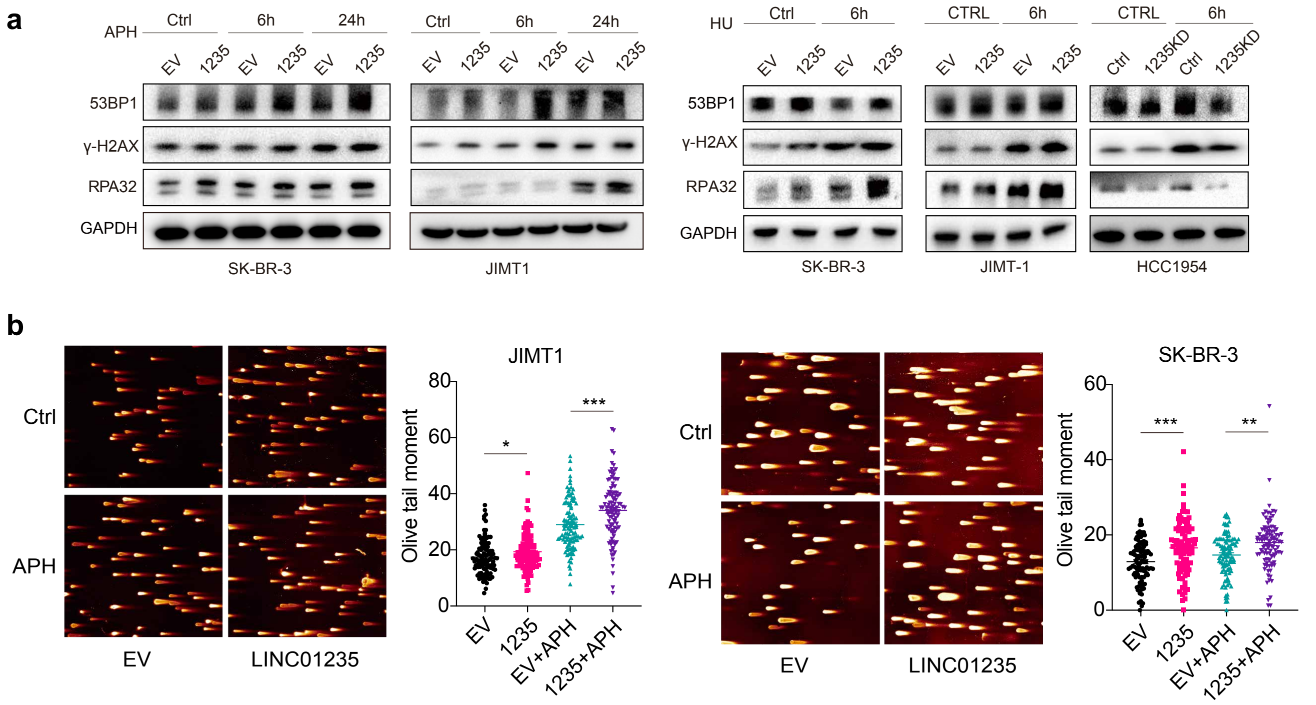


**Figure S4. a**, Left: Immunoblot showing indicated DNA damage response proteins in SK-BR-3 and JIMT1 cells overexpressed with EV and LINC01235 treated with 1 μM aphidicolin (APH) or control for 6 and 24 h. Right: cells were treated with 10 mM hydroxyurea (HU) for 6 h. n=2 experiments. **b**, Neutral comet assays showing DNA breaks in SK-BR-3 and JIMT1 cells overexpressing EV or 1235. Cells were treated with 1 μM APH or control for 24 h. The representative comet images are shown at the top of the figures. Tail moments were quantified in 100 cells from two independent experiments using the CaspLab software. Statistical analysis was performed using a two-sided t-test. * $P$<0.05, ** $P$<0.01, *** $P$<0.001. Error bars represent mean ± SEM.

# Figure S5

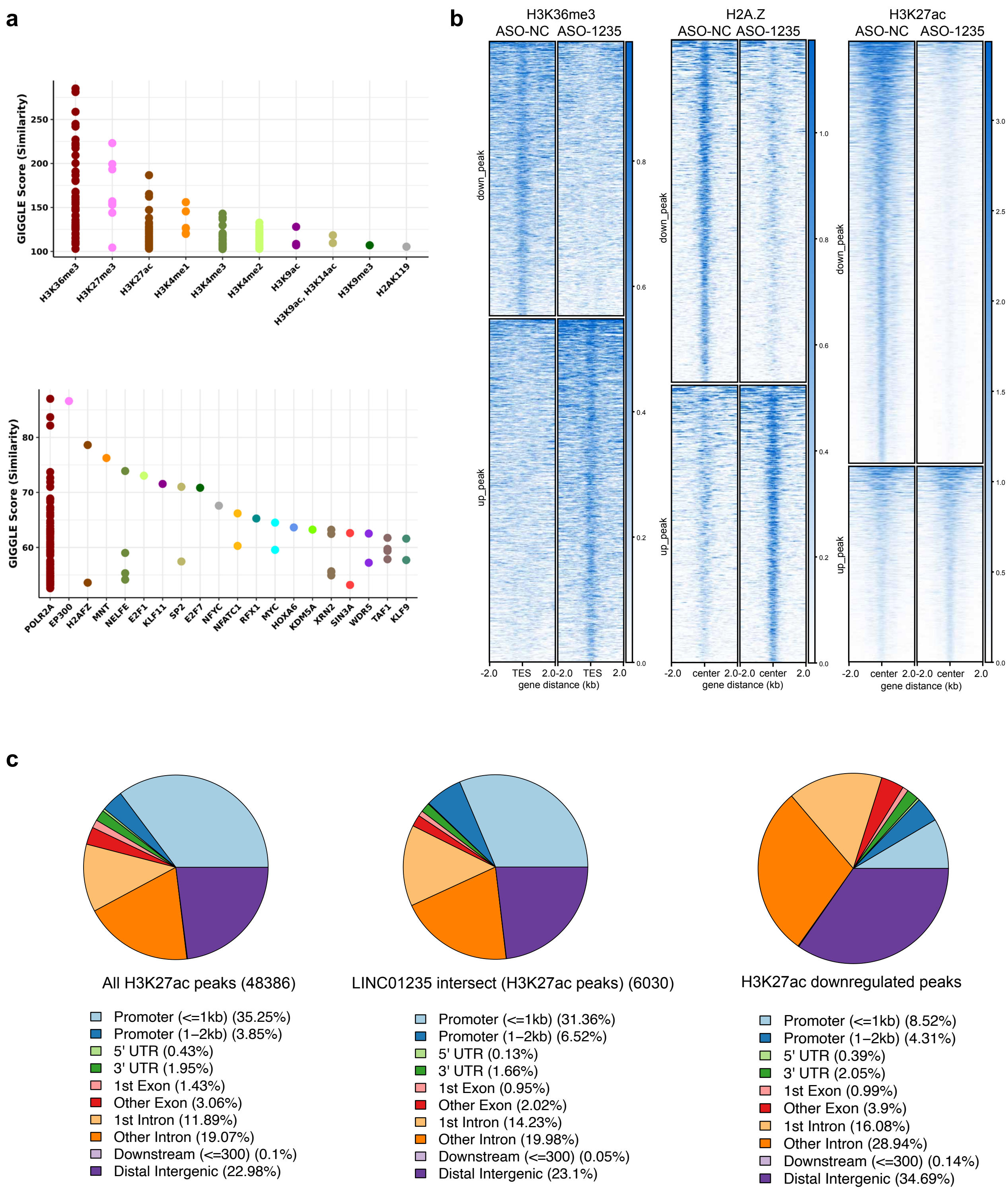


**Figure S5.** **a,** Histone markers and transcriptional factors predicted with the binding sites of the 20,000 most differentially enriched peaks of LINC01235. The ChIRP-seq data was analyzed using the Toolkit for Cistrome Data Browser. **b**, Heatmaps of H3K36me3, H2AZ, and H3K27ac ChIP-seq signals that were significantly upregulated or downregulated following LINC01235 depletion. **c**, Genomic distribution of regions showing H3K27ac binding sites (left), LINC01235 ChIRP-seq peaks intersecting with H3K27ac peaks (middle), and H3K27ac downregulation peaks after LINC01235 depletion (right) in HCC1954 cells.

Figure S6

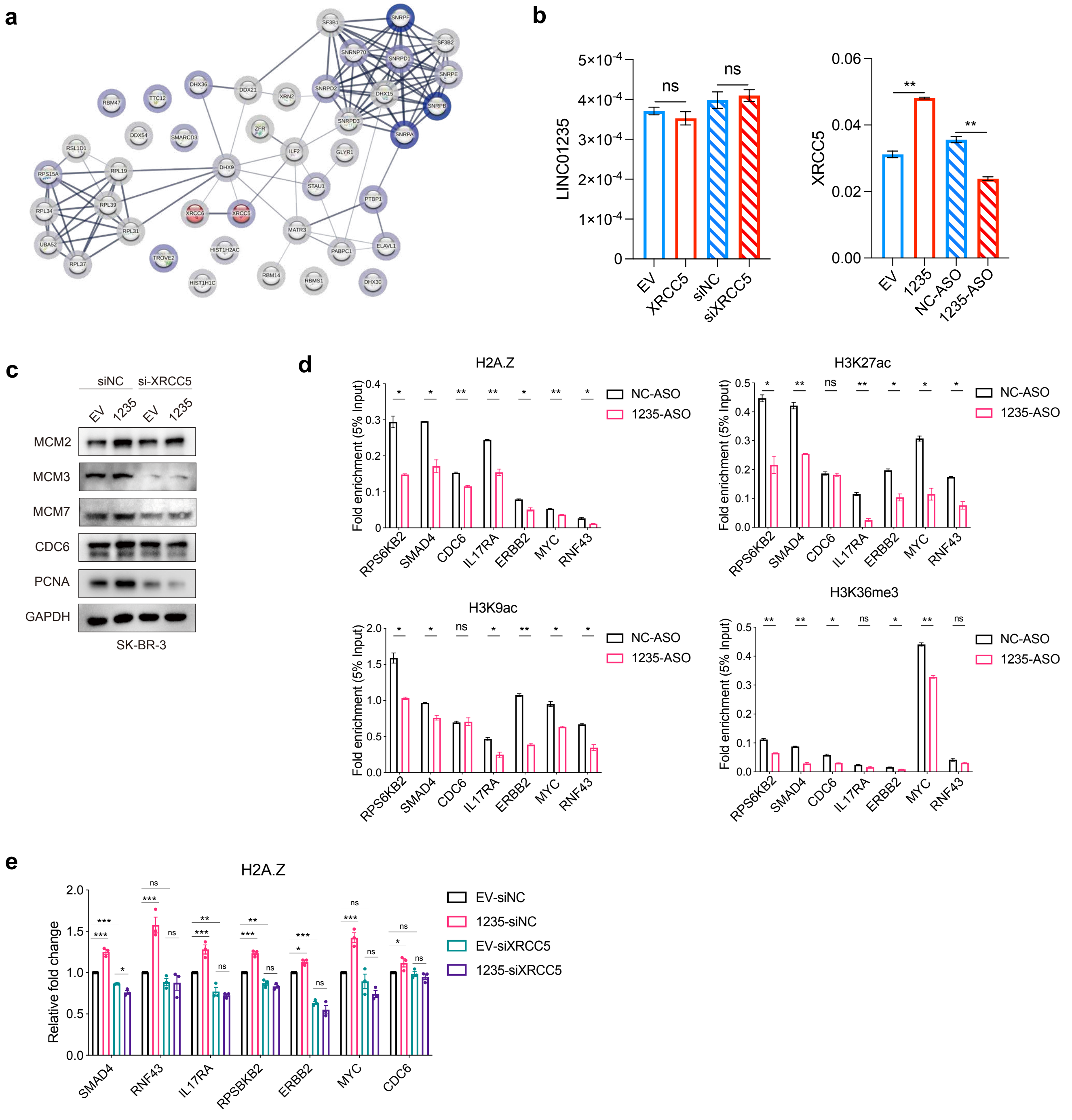


**Figure S6. a**, STRING analysis of the LINC01235 interactome determined by RNA pull-down/MS. **b**, RT-qPCR quantification of LINC01235 and XRCC5 expression after the indicated treatments. n=3 biological replicates. GAPDH was used as a reference gene. **c**, Immunoblot showing DNA replication licensing-related proteins in SK-BR-3 cells overexpressing EV and LINC01235 treated with siXRCC5 or siNC for 72 h. n=2 experiments. **d**, RT-qPCR results of H2AZ, H3K27ac, H3K9ac, and H3K36me3 ChIP experiments in HCC1954 cells. Seven genes with LINC01235 binding to the promoter regions were selected. n=3 biological replicates. **e**, H2AZ ChIP-qPCR results in HCC1954 cells. n=3 biological replicates. *P* values were calculated using one-way ANOVA. * *P*<0.05, ** *P*<0.01, *** *P*<0.001, ns not significiant. Error bars represent mean ± SEM.

# Figure S7

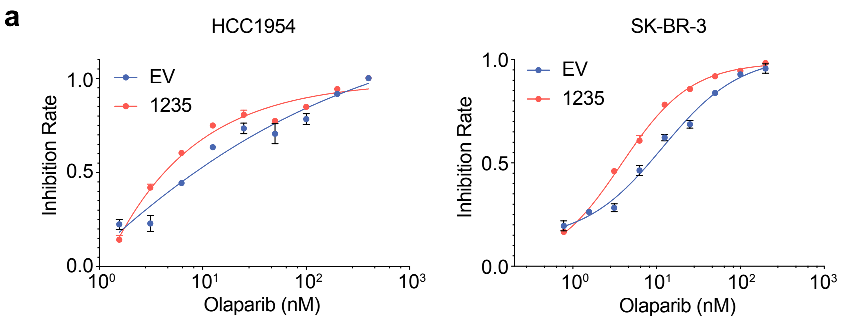


**Figure S7. a**, Growth inhibition assays for olaparib were performed in HCC1954 and SK-BR-3 cells. n=3 for each concentration. Error bars represent mean ± SEM (standard error of mean).

**Table S1. Patient Clinical information of FUSCC NAC cohort**

| ID | Sex | Age at surgery | Baseline | | | | | Clincal TNM | | | Pathological results after surgery | | | | |
|---|---|---|---|---|---|---|---|---|---|---|---|---|---|---|---|
| | | | ER | PR | HER2 | Ki-67 | HER2 FISH | T | N | M | Residual primary tumor | LN metastasis | LN results | Response | Treatment |
| 1822 | Female | 32 | 0 | 0 | 3 | 20 | 1 | 3 | 1 | 0 | 0 | 0 | 0/15 | pCR | PCbH*6 |
| 1863 | Female | 41 | 0 | 0 | 3 | 30 | 1 | 2 | 1 | 0 | 0 | 0 | 0/18 | pCR | PCbH*6 |
| 2123 | Female | 54 | 0 | 0 | 3 | 30 | NA | 2 | 1 | 0 | 0 | 0 | 0/10 | pCR | PCbH*6 |
| 2148 | Female | 37 | 0 | 0 | 3 | 50 | 1 | 2 | 1 | 0 | 0 | 0 | 0/12 | pCR | PCbH*6 |
| 2169 | Female | 52 | 0 | 0 | 3 | 50 | 1 | 2 | 1 | 0 | 0 | 1 | 2/20 | non-pCR | PCbH*6 |
| 2237 | Female | 44 | 0 | 0 | 3 | 70 | 1 | 2 | 1 | 0 | 0 | 0 | 0/3 | pCR | PCbH*6 |
| 2270 | Female | 46 | 0 | 0 | 3 | 60 | 1 | 2 | 3 | 0 | 0 | 0 | 0/9 | pCR | PCbH*6 |
| 2385 | Female | 50 | 0 | 0 | 3 | 70 | 1 | 2 | 1 | 0 | 0 | 0 | 0/12 | pCR | PCb*3-FEC*4 |
| 2438 | Female | 55 | 0 | 3% weak | 3 | 80 | 1 | 2 | 1 | 0 | 0 | 0 | 0/4 | pCR | PCbH*4-ECH*4 |
| 2504 | Female | 58 | 0 | 0 | 3 | 40 | 1 | 2 | 1 | 0 | 0 | 0 | 0/12 | pCR | PCbH*4 |
| 2519 | Female | 50 | 0 | 0 | 3 | 70 | 1 | 2 | 3 | 0 | 0 | 0 | 0/5 | pCR | PCbH*4-ECH*4 |
| 2564 | Female | 45 | 0 | 0 | 3 | 40 | 1 | 2 | 1 | 0 | 0 | 0 | 0/9 | pCR | PCbH*6 |
| 2586 | Female | 43 | 0 | 0 | 3 | 20 | 1 | 2 | 1 | 0 | 0 | 0 | 0/14 | pCR | PCbH*4-ECH*4 |
| 2605 | Female | 33 | 0 | 0 | 3 | 50 | 1 | 2 | 3 | 0 | 0 | 0 | 0/6 | pCR | PCbH*6 |
| 2698 | Female | 51 | 0 | 0 | 3 | 20 | NA | 2 | 0 | 0 | 0 | 0 | 0/13 | pCR | PCbH*4 |
| 2708 | Female | 52 | 0 | 0 | 3 | 60 | 1 | 3 | 1 | 0 | 0 | 0 | 0/15 | pCR | PCbH*6 |
| 2828 | Female | 61 | 0 | 0 | 3 | 80 | 1 | 4 | 3 | 0 | 0 | 1 | 1/4 | non-pCR | PH*16-ECH*4 |
| 4024 | Female | 41 | 0 | 0 | 3 | 30 | 1 | 2 | 1 | 0 | 0 | 0 | 0/4 | pCR | PCbH*6 |
| 4034 | Female | 60 | 0 | 0 | 3 | 30 | 1 | 2 | 1 | 0 | 0 | 1 | 1/19 | non-pCR | PCbH*6 |
| 4046 | Female | 41 | 0 | 0 | 3 | 20 | 1 | 2 | 1 | 0 | 0 | 0 | 0/23 | pCR | PCbH*7 |
| 4048 | Female | 69 | 0 | 0 | 2 | 40 | 1 | 2 | 1 | 0 | 0 | 1 | 5/15 | non-pCR | PCbH*6 |
| 4050 | Female | 45 | 0 | 0 | 3 | 80 | 1 | 2 | 1 | 0 | 0 | 0 | 0/16 | pCR | PCbH*6 |
| 4304 | Female | 57 | 0 | 0 | 2 | 40 | 1 | 4 | 1 | 0 | 0 | 0 | 0/3 | pCR | PCbH*8 |
| 4362 | Female | 75 | 0 | 0 | 3 | 50 | 1 | 4 | 3 | 0 | 0 | 0 | 0/17 | pCR | PCbH*6 |
| 4383 | Female | 56 | 0 | 0 | 3 | 30 | 1 | 3 | 1 | 0 | 0 | 1 | 1/22 | non-pCR | PCbH*6 |
| 4398 | Female | 69 | 0 | 0 | 3 | 25 | 1 | 2 | 1 | 0 | 0 | 0 | 0/29 | pCR | PCbH*4 |
| 4550 | Female | 56 | 0 | 0 | 3 | 50 | 1 | 2 | 3 | 0 | 0 | 1 | 3/16 | non-pCR | PCbH*6 |
| 4599 | Female | 61 | 0 | 0 | 3 | 30 | 1 | 2 | 0 | 0 | 0 | 0 | 0/12 | pCR | PCbH*4 |
| 4607 | Female | 46 | 0 | 0 | 3 | 30 | 1 | 2 | 3 | 0 | 0 | 0 | 0/5 | pCR | PH*16-ECH*4 |
| 4618 | Female | 45 | 0 | 0 | 3 | 30 | 1 | 2 | 1 | 0 | 0 | 0 | 0/4 | pCR | PCbH*4-ECH*4 |
| 4786 | Female | 61 | 0 | 0 | 3 | 40 | 1 | 2 | 1 | 0 | 0 | 0 | 0/15 | pCR | PCbH*6 |
| 4787 | Female | 55 | 0 | 0 | 3 | 30 | 1 | 2 | 3 | 0 | 0 | 0 | 0/17 | pCR | PCbH*6 |
| 4820 | Female | 62 | 0 | 0 | 3 | 20 | 1 | 4 | 1 | 1 | 0 | 0 | 0/0 | pCR | PCbH*6 |
| 4892 | Female | 49 | 0 | 0 | 3 | 60 | 1 | 2 | 1 | 0 | 0 | 0 | 0/12 | pCR | PCbH*6 |
| 4895 | Female | 63 | 0 | 0 | 3 | 70 | 1 | 2 | 1 | 0 | 0 | 0 | 0/15 | pCR | PCbH*4 |
| 4896 | Female | 48 | 0 | 0 | 3 | 25 | 1 | 2 | 1 | 0 | 0 | 0 | 0/12 | pCR | PCbH*6 |
| 4917 | Female | 52 | 0 | 0 | 3 | 25 | 1 | 2 | 3 | 0 | 0 | 0 | 0/15 | pCR | PCbH*4 |
| 4981 | Female | 70 | 0 | 0 | 3 | 30 | 1 | 2 | 1 | 0 | 0 | 0 | 1/15 | pCR | PCbH*6 |
| 5014 | Female | 30 | 0 | 0 | 3 | 80 | 1 | 4 | 1 | 0 | 0 | 0 | 0/24 | pCR | PCbH*6 |
| 5126 | Female | 43 | 0 | 0 | 3 | 40 | 1 | 2 | 1 | 0 | 0 | 0 | 0/12 | pCR | PCbH*6 |
| 1813 | Female | 61 | 0 | 0 | 3 | 80 | NA | 2 | 0 | 0 | 1 | 0 | 0/18 | non-pCR | PCbH*6 |
| 1925 | Female | 61 | 0 | 0 | 3 | 70 | NA | 2 | 1 | 0 | 1 | 0 | 0/19 | non-pCR | PCbH*6 |
| 2069 | Female | 51 | 0 | 0 | 2 | 40 | 1 | 2 | 1 | 0 | 1 | 0 | 0/4 | non-pCR | PCbH*6 |
| 2145 | Female | 61 | 0 | 0 | 3 | 20 | 1 | 2 | 1 | 0 | 1 | 0 | 0/10 | non-pCR | PCbH*6 |
| 2216 | Female | 49 | 0 | 0 | 3 | 30 | 1 | 2 | 0 | 0 | 1 | 0 | 0/15 | non-pCR | PCbH*6 |
| 2494 | Female | 55 | 0 | 0 | 3 | 70 | 1 | 3 | 1 | 0 | 1 | 1 | 4/21 | non-pCR | PCbH*6 |
| 2529 | Female | 66 | 0 | 5% weak | 2 | 20 | 1 | 3 | 3 | 0 | 1 | 1 | 2/8 | non-pCR | PCbH*4-ECH*4 |
| 2649 | Female | 54 | 0 | 0 | 3 | 40 | 1 | 2 | 1 | 0 | 1 | 1 | 2/16 | non-pCR | PCbH*6 |
| 4482 | Female | 52 | 0 | 0 | 2 | 30 | 1 | 3 | 1 | 0 | 1 | 0 | 0/5 | non-pCR | PCbH*6 |
| 4553 | Female | 33 | 0 | 0 | 3 | 50 | 1 | 1 | 1 | 0 | 1 | 0 | 0/5 | non-pCR | PCbH*6 |
| 4617 | Female | 54 | 0 | 0 | 3 | 15 | 1 | 2 | 2 | 0 | 1 | 0 | 0/22 | non-pCR | PCbH*6 |
| 4715 | Female | 61 | 0 | 0 | 9 | 60 | 1 | 2 | 1 | 0 | 1 | 0 | 0/17 | non-pCR | PCbH*4 |
| 4761 | Female | 46 | 0 | 0 | 3 | 60 | 1 | 2 | 1 | 0 | 1 | 1 | 9/18 | non-pCR | PCbH*6 |
| 4835 | Female | 42 | 0 | 0 | 3 | 30 | 1 | 3 | 1 | 0 | 1 | 0 | 0/21 | non-pCR | PCbH*4 |
| 4863 | Female | 45 | 0 | 0 | 3 | 40 | 1 | 2 | 1 | 0 | 1 | 1 | 1/19 | non-pCR | EC*4-PH*4 |
| 4927 | Female | 45 | 0 | 0 | 3 | 40 | 1 | 2 | 1 | 0 | 1 | 0 | 0/15 | non-pCR | PCbH*6 |
| 4952 | Female | 63 | 0 | 0 | 3 | 80 | 1 | 2 | 1 | 0 | 1 | 0 | 0/19 | non-pCR | PCbH*6 |
| 4959 | Female | 64 | 0 | 0 | 3 | 30 | 1 | 2 | 1 | 0 | 1 | 1 | 2/25 | non-pCR | PCbH*6 |
| 5124 | Female | 36 | 0 | 0 | 3 | 50 | 1 | 4 | 1 | 0 | 1 | 0 | 0/14 | non-pCR | PCbH*6 |
| 4458 | Female | 56 | 0 | 0 | 3 | 20 | 1 | 3 | 1 | X | 1 | 1 | NA | non-pCR | PCbH*6 |
| 4499 | Female | 56 | 0 | 0 | 3 | 40 | 1 | 2 | 2 | X | 1 | 1 | NA | non-pCR | PCbH*6 |
| 4297 | Female | 42 | 0 | 0 | 1 | 60 | 1 | 2 | 1 | 0 | 0 | 0 | 0/20 | pCR | PCbH*6 |
| 4029 | Female | 51 | 0 | 0 | 3 | 15 | 1 | 2 | 1 | 1 | 1 | 1 | 3/14 | non-pCR | PCbH*6 |

For residual primary tumor and LN metastasis, "0" means no, "1" means yes. For HER2 FISH, "0" means no amplification, "1" means amplification. For treatment, "P" means taxane based chemotherapy, "Cb" means carboplatin, "H" means trastuzumab, "E" meas epirubicin, "C" means cyclophosphamide, "F" means 5-fluorouracil. NA, not available; ER, estrogen receptor; PR, progesterone receptor; HER2, human epidermal growth factor receptor 2; FISH, fluorescence in situ hybridization; pCR, pathological complete response.

**Table S2. Patient Clinical information of FUSCC NAC cohort**

| ID | Sex | Age_at_surgery | Baseline | | | | | Clincal TNM | | | Pathological results after surgery | | | | Treatment |
|---|---|---|---|---|---|---|---|---|---|---|---|---|---|---|---|
| | | | ER | PR | HER2 | Ki-67 | FISH | T | N | M | dual Primary T | N metastasis | LN | Response | |
| 5468 | Female | 52 | 0 | 0 | 3 | 30 | 1 | 2 | 1 | 0 | 0 | 0 | 0/18 | pCR | TPyH*4 |
| 5509 | Female | 48 | 0 | 0 | 3 | 20 | 1 | 2 | 1 | 0 | 0 | 0 | 0/13 | pCR | TPyH*4 |
| 5531 | Female | 56 | 0 | 0 | 3 | 30 | 1 | 2 | 1 | 0 | 0 | 0 | 0/18 | pCR | TPyH*4 |
| 5219 | Female | 64 | 0 | 0 | 3 | 20 | NA | 2 | 1 | 0 | 0 | 0 | 0/13 | pCR | TPyH*4 |
| 5312 | Female | 55 | 0 | 0 | 3 | 30 | 1 | 2 | 1 | 0 | 0 | 0 | 0/15 | pCR | TPyH*4 |
| 5339 | Female | 51 | 0 | 0 | 3 | 70 | 1 | 2 | 1 | 0 | 0 | 0 | 0/11 | pCR | TH*4 |
| 5226 | Female | 48 | 0 | 0 | 3 | 50 | NA | 2 | 1 | 0 | 0 | 0 | 0/4 | pCR | TPyH*4 |
| 5991 | Female | 50 | 0 | 0 | 3 | 30 | 1 | 2 | 1 | 0 | 0 | 0 | 0/3 | pCR | TPyH*4 |
| 6145 | Female | 42 | 0 | 0 | 3 | 30 | 1 | 2 | 1 | 0 | 0 | 0 | 0/4 | pCR | TPyH*4 |
| 6820 | Female | 66 | 0 | 0 | 3 | 60 | 1 | 2 | 1 | 0 | 0 | 0 | 0/2 | pCR | TPyH*4 |
| 7078 | Female | 46 | 0 | 0 | 3 | 30 | 1 | 2 | 1 | 0 | 0 | 0 | 0/13 | pCR | TPyH*4 |
| 7285 | Female | 60 | 0 | 0 | 3 | 30 | 1 | 2 | 1 | 0 | 0 | 0 | 0/16 | pCR | TH*4 |
| 5068 | Female | 35 | 0 | 0 | 3 | 60 | NA | 3 | 1 | 0 | 0 | 0 | 0/13 | pCR | TPyH*4 |
| 5202 | Female | 62 | 0 | 0 | 3 | 30 | 1 | 2 | 1 | 0 | 1 | 0 | 0/5 | non-pCR | TPyH*4 |
| 5262 | Female | 24 | 0 | 0 | 3 | 30 | 1 | 2 | 1 | 0 | 1 | 0 | 0/20 | non-pCR | TH*4 |
| 5291 | Female | 52 | 0 | 0 | 2 | 20 | 1 | 3 | 1 | 0 | 1 | 0 | 0/16 | non-pCR | TH*4 |
| 5298 | Female | 53 | 0 | 0 | 3 | 50 | 1 | 2 | 1 | 0 | 1 | 1 | 1/10 | non-pCR | TPyH*4 |
| 5347 | Female | 48 | 0 | 0 | 3 | 30 | 1 | 3 | 1 | 0 | 1 | 0 | 0/16 | non-pCR | TH*4 |
| 5354 | Female | 56 | 0 | 0 | 3 | 30 | 1 | 2 | 1 | 0 | 1 | 1 | 7/22 | non-pCR | TPyH*4 |
| 5575 | Female | 35 | 0 | 0 | 3 | 50 | 1 | 2 | 1 | 0 | 1 | 0 | 0/25 | non-pCR | TPyH*4 |
| 5642 | Female | 54 | 0 | 0 | 3 | 40 | 1 | 2 | 1 | 0 | 1 | 1 | 1/21 | non-pCR | TPyH*4 |
| 5733 | Female | 55 | 0 | 0 | 3 | 40 | 1 | 2 | 1 | 0 | 1 | 1 | 3/14 | non-pCR | TPyH*4 |
| 6006 | Female | 38 | 0 | 0 | 3 | 30/40 | 1 | 2 | 1 | 0 | 1 | 0 | 0/6 | non-pCR | TPyH*4 |
| 6329 | Female | 56 | 0 | 0 | 3 | 40 | 1 | 2 | 1 | 0 | 1 | 1 | 4/17 | non-pCR | TH*4 |
| 6559 | Female | 62 | 0 | 0 | 3 | 2 | 1 | 2 | 1 | 0 | 1 | 1 | 4/15 | non-pCR | TH*4 |

For residual primary tumor and LN metastasis, "0" means no, "1" means yes. For HER2 FISH, "0" means no amplification, "1" means amplification. For treatment, "T" means taxane based chemotherapy, "Py" means pyrotinib, "H" means trastuzumab. NA, not available; ER, estrogen receptor; PR, progesterone receptor; HER2, human epidermal growth factor receptor 2; FISH, fluorescence in situ hybridization; pCR, pathological complete response.

**Table S3 Conservation Analaysis of LINC01235 by PLAR database**

| Symbol | Transcripts With Conservation | Non-redundant Exons | Species With Seq Orthologs | Species With BLAST | Species With WGA | Species With Synteny | Species With Sequence And Synteny | Mouse BLAST | Mouse WGA | Mouse Synteny | Mouse Sequence+Synteny |
|---|---|---|---|---|---|---|---|---|---|---|---|
| NEAT1 | ENST00000499732.3_2, ENST00000501122.2_1, ENST00000601801.3_2, ENST00000612303.2_2, ENST00000616315.2_2, ENST00000642367.1_1, ENST00000645023.1_1, ENST00000646243.1_1 | 1 | Mouse, Dog, Ferret, Marmoset, Opossum, Rabbit | Mouse, Dog, Ferret, Marmoset, Opossum, Rabbit | Mouse, Dog, Ferret, Marmoset | Mouse, Dog, Ferret, Gar, Lizard, Marmoset, Opossum | Mouse, Dog, Ferret, Marmoset, Opossum | ENSMUST00000173672.1, ENSMUST00000174287.1, ENSMUST00000174829.1, ENSMUST00000232969.1 | ENSMUST00000173672.1, ENSMUST00000174287.1, ENSMUST00000174829.1 | ENSMUST00000172812.2, ENSMUST00000173314.1, ENSMUST00000173499.1, ENSMUST00000173672.1, ENSMUST00000174287.1, ENSMUST00000174808.1, ENSMUST00000174829.1, ENSMUST00000232969.1 | ENSMUST00000173672.1, ENSMUST00000174287.1, ENSMUST00000174829.1, ENSMUST00000232969.1 |
| LINC01235 | ENST00000604724.5_1, ENST00000605459.1_2 | 5 | - | - | - | Chicken, Dog, Ferret, Gar, Lizard, Marmoset, Mouse, Rabbit, Rhesus | - | - | - | ENSMUST00000137477.1, ENSMUST00000143755.1, ENSMUST00000148635.1 | - |

Data were extracted from PLAR database. BLAST: Basic Local Alignment Search Tool. WGA: Whole-Genome Amplification. NEAT1 were shown as control.

**Supplementary File 1**

**Isoform 1**

GTGCAACACCTGAAATTCCTGGGGCGAATCAAACAGCAAATTAAAGCTCAGACAACCTTCA
CCTGCAAACCATGCACAGTATAAGGAACTCATTTTAATGTTTTCTGGCTGAAGTCATAGGGG
ATAAGAAAACAAAAGAATGGCCACGAAGGAATCCTGGTAGAGCTCCAGGAATACATTCTGAA
TGCCAAAAACCATGGGAAGAGGAAGAAGGAAGAGTTTTGCCTTTGAGAGAAAGATGCATTG
AAATGAAGAAAGAGAAAATAATCTTAGAAGAGTATAGAGGACATTGTTACCATTTACCTCCAC
TGGATATCTATTTTCTTTCAAAAAGCACAATCAACGTGCCTTTTCCCTGATTTTCTCTGCCAAA
CATATCAGATTGTGGGAGGTTTATGGATACACAGTACTTCACAGGCAACCTGAAAGGTCCCA
ATTTGGGCCCCGAGTCTGCTGGAGAAAAGAGAAAGGCTAAGAAGCAGCCTACCTTACCTGT
GGCTCTGGGTCTGAGGATGGCCAGGAGAACAGAGAGCAAAGCAGAGCCAATGCAAGACCT
CTGGCCCTGGAAGCCGAAGGGTCTCTCACAGGTCAACGCAGGATCTACTCCATCATCATCT
TCACTAGCACACCCAGTTCGATTGAGGAGCCTTGACGTGGGATATTGATGGGATATTGAGAG
AAAAGAGAAATGGTGAATCAAGCACTAATCTCAAAGCTTCTGCACAGAACTACTTACATGTCA
CTTCCTCCCATGACTATTCCTAAATTCAGCCAAAAGGGGACACATAATCTCTTACGGGGGAG
GGCACCACGGGGAGAGAAACCCGAATATTTGGTGAATAGTAACACAATCTACTTTGTTATAG
AATCTGTCCTCAATGGTCTTACAGTCTAAGAGCATTACATTTAGTTACGGTAGTCTTAAATATG
ATACTAAATGAATTGAATAGCCAGACATCTGTGAAAGTAAAACTGTGATAATACAAAGTCAGT
GTGCTTTTAAGTTTCAATTGCTTAAAGTTATTTGGAAGTAAGGATCACATGAGTTCAGAGCCC
CAGGATTCAAAAATAACTCCGGAATCTTTTATCACTCCCCAAAGGAAAAGCAATAATAAGTAA
GTTACCAGACATTCATGTAATCTCTCTGAAACTGTAAGACTAACAAAGGAAAATTTTCAAAAG
GCAGAAATACTTTATTATTTATTTGCAATAGAAATTATTCAGCCGGGCGCGGTGGCTCATGCC
TGTAATCCCAGCACTTTGGGAGGCCGAGGCGAGTGGATCACCTGAGGTCGGGAGTTCGAG
ACCAGCCTGACCAACATGGAGAAACCCCGTCTCTACTAAAAATACACAATTAGCTGGGCATG
GTGGCACATGCCTGCAAGCCCAGCTACTCAGGAGGCTAAGGCAGGAGCGACAGAGCGAG
ACTCCATCTCAAAAAAAAAAGAAAGAAAGAAAGGAAAAAAAGAAAAGAAAAGAAACAAAGAA
ATTTTTCAGTAAATACATACAACAGCATTGATGGCCTGGAGACATACCACCTTCAGTTGTGAT
CCTGGCAGATGTGATAGATTCTACAAGGTTGGACAGGTTAAATTCTTGCAAGGAAGTCCTTT
CTGGAACACCCAAGAATTACAGAAAGTTTAGCTATCTTTCAGATGAGATTTCATTCTCAGTTT
TACTTGTTCCATGACCCTTCCAAACTACACACGGGATTCTTTTCATTCAGTTGCCTTGGCCA
GCATTTTATCACCAATAGTCCTATTTTATGTAACTGAGTTTGCCTTGCCCTTTACTGCATTTAG
GTAAATGGAATGGTGTTTTTTAGACTTCCGAAACTATTTGTAGACCTTTTCCTTCACAAGTTT
GAAATAGATGTGAGGCATTCATTTTACTCAGACAACATTGAGCCCAATTCACTTGGGAAAATG
CATCTTGATGTGTAATTCATCATCCAGAGCCTGTCCACTATTCTGTTCCTCCCTGAGAGTAAA
TTAGGCACTTTGAAAGGTGCATTAAACTCTTCCTGGCTACTAGGTACCATTTAGAACTGTGG
GGCAATAATTTACTTCATGGTACCTTGGTGACCTCAAAAAGTTTATATTGTTTTGAAAGTTCTT
AATTTTCTCCCACTAAATAATTTATGCATTCAGAGGTTGGGTGTGCTTATGTTTCCAGAGAGA
CAACAGCTATTGAATAGCATAAATAAGATAAAATAGTCATGCTAGTGTAGAAATTAAATGTTCA
CAAATGTGTTATCGAGTCTATCCATAGAATAAAAGTGTAGCTTATAAAACAATTATTGCTTTGG
ATTTCATGTTTTAAATTTACAAGACTGTGTTACATTTTGTTGTAATTAGTAATGCTTCATAGCTA
CAAGGATTACCAGATCCATATCACTGCATTTTTCCATAGCACAAAACACATTTATCCAAAGTAA
AAATAGTGTGCTAAACATACTGAAGTTTCTGACAGACACTTATATTTCTCTTCATTCTTTCATT

CACAGCAGAGTTTCATCCATATTAAATAAATTGGTTAAGTTTTGATACCTCCTACTTTAATCACT
CATCTTATGTACTAAAATGAACCAAATTAAGTTTGAGCCAACAAATAAAAGTGATATGCTTTGA

**Isoform 2**

TAATACGACTCACTATAGGGGTGCAACACCTGAAATTCCTGGGGCGAATCAAACAGCAAATT
AAAGCTCAGACAACCTTCACCTGCAAACCATGCACAGTATAAGGAACTCATTTTAATGTTTTC
TGGCTGAAGTCATAGGGGATAAGAAAACAAAAGAATGGCCACGAAGGAATCCTGGACATTG
TTACCATTTACCTCCACTGGATATCTATTTTCTTTCAAAAAGCACAATCAACGTGCCTTTTCCC
TGATTTTCTCTGCCAAACATATCAGATTGTGGGAGGTTTATGGATACACAGTACTTCACAGGC
AACCTGAAAGGTCCCAATTTGGGCCCCGAGTCTGCTGGAGAAAAGAGAAAGGCTAAGAAG
CAGCCTACCTTACCTGTGGCTCTGGGTCTGAGGATGGCCAGGAGAACAGAGAGCAAAGCA
GAGCCAATGCAAGACCTCTGGCCCTGGAAGCCGAAGGGTCTCTCACAGGTCAACGCAGGA
TCTACTCCATCATCATCTTCACTAGCACACCCAGTTCGATTGAGGAGCCTTGACGTGGGATA
TTGATGGGATATTGAGAGAAAAGAGAAATGGTGAATCAAGCACTAATCTCAAAGCTTCTGCA
CAGAACTACTTACATGTCACTTCCTCCCATGACTATTCCTAAATTCAGCCAAAAGGGGACACA
TAATCTCTTACGGGGGAGGGCACCACGGGGAGAGAAACCCGAATATTTGGTGAATAGTAAC
ACAATCTACTTTGTTATAGAATCTGTCCTCAATGGTCTTACAGTCTAAGAGCATTACATTTAGT
TACGGTAGTCTTAAATATGATACTAAATGAATTGAATAGCCAGACATCTGTGAAAGTAAAACTG
TGATAATACAAAGTCAGTGTGCTTTTAAGTTTCAATTGCTTAAAGTTATTTGGAAGTAAGGATC
ACATGAGTTCAGAGCCCCAGGATTCAAAAATAACTCCGGAATCTTTTATCACTCCCCAAAGG
AAAAGCAATAATAAGTAAGTTACCAGACATTCATGTAATCTCTCTGAAACTGTAAGACTAACAA
AGGAAAATTTTCAAAAGGCAGAAATACTTTATTATTTATTTGCAATAGAAATTATTCAGCCGGG
CGCGGTGGCTCATGCCTGTAATCCCAGCACTTTGGGAGGCCGAGGCGAGTGGATCACCTG
AGGTCGGGAGTTCGAGACCAGCCTGACCAACATGGAGAAACCCCGTCTCTACTAAAAATAC
ACAATTAGCTGGGCATGGTGGCACATGCCTGCAAGCCCAGCTACTCAGGAGGCTAAGGCA
GGAGCGACAGAGCGAGACTCCATCTCAAAAAAAAAAGAAAGAAAGAAAGGAAAAAAAGAAA
AGAAAAGAAACAAAGAAATTTTTCAGTAAATACATACAACAGCATTGATGGCCTGGAGACATA
CCACCTTCAGTTGTGATCCTGGCAGATGTGATAGATTCTACAAGGTTGGACAGGTTAAATTC
TTGCAAGGAAGTCCTTTCTGGAACACCCAAGAATTACAGAAAGTTTAGCTATCTTTCAGATG
AGATTTCATTCTCAGTTTTACTTGTTCCATGACCCTTCCAAACTACACACGGGATTCTTTTCAT
TCAGTTGCCTTGGCCAGCATTTTATCACCAATAGTCCTATTTTATGTAACTGAGTTTGCCTTG
CCCTTTACTGCATTTAGGTAAATGGAATGGTGTTTTTTAGACTTCCGAAACTATTTGTAGACCT
TTTCCTTCACAAGTTTGAAATAGATGTGAGGCATTCATTTTACTCAGACAACATTGAGCCCAA
TTCACTTGGGAAAATGCATCTTGATGTGTAATTCATCATCCAGAGCCTGTCCACTATTCTGTT
CCTCCCTGAGAGTAAATTAGGCACTTTGAAAGGTGCATTAAACTCTTCCTGGCTACTAGGTA
CCATTTAGAACTGTGGGGCAATAATTTACTTCATGGTACCTTGGTGACCTCAAAAAGTTTATAT
TGTTTTGAAAGTTCTTAATTTTCTCCCACTAAATAATTTATGCATTCAGAGGTTGGGTGTGCTT
ATGTTTCCAGAGAGACAACAGCTATTGAATAGCATAAATAAGATAAAATAGTCATGCTAGTGTA
GAAATTAAATGTTCACAAATGTGTTATCGAGTCTATCCATAGAATAAAAGTGTAGCTTATAAAA
CAATTATTGCTTTGGATTTCATGTTTTAAATTTACAAGACTGTGTTACATTTTGTTGTAATTAGT
AATGCTTCATAGCTACAAGGATTACCAGATCCATATCACTGCATTTTTCCATAGCACAAAACAC
ATTTATCCAAAGTAAAAATAGTGTGCTAAACATACTGAAGTTTCTGACAGACACTTATATTTCT

CTTCATTCTTTCATTCACAGCAGAGTTTCATCCATATTAAATAAATTGGTTAAGTTTTGATACCT
CCTACTTTAATCACTCATCTTATGTACTAAAATGAACCAAATTAAGTTTGAGCCAACAAATAAA
AGTGATATGCTTTGA

**Isoform 3**

GTGCAACACCTGAAATTCCTGGGGCGAATCAAACAGCAAATTAAAGCTCAGACAACCTTCA
CCTGCAAACCATGCACAGTATAAGGAACTCATTTTAATGTTTTCTGGCTGAAGTCATAGGGG
ATAAGAAAACAAAAGAATGGCCACGAAGGAATCCTGGACATTGTTACCATTTACCTCCACTG
GATATCTATTTTCTTTCAAAAAGCACAATCAACGTGCCTTTTCCCTGATTTTCTCTGCCAAACA
TATCAGATTGTGGGAGGTTTATGGATACACAGTACTTCACAGGCAACCTGAAAGGTCCCAAT
TTGGGCCCCGAGTCTGCTGGAGAAAAGAGAAAGGCTAAGAAGCAGCCTACCTTACCTGTG
GCTCTGGGTCTGAGGATGGCCAGGAGAACAGAGAGCAAAGCAGAGCCAATGCAAGACCTC
TGGCCCTGGAAGCCGAAGGGTCTCTCACAGGTCAACGCAGGATCTACTCCATCATCATCTT
CACTAGCACACCCAGTTCGATTGAGGAGCCTTGACGTGGGATATTGATGGGATATTGAGAG
AAAAGAGAAATGGTGAATCAAGCACTAATCTCAAAGCTTCTGCACAGAACTACTTACATGTCA
CTTCCTCCCATGACTATTCCTAAATTCAGCCAAAAGGGGACACATAATCTCTTACGGGGGAG
GGCACCACGGGGAGAGAAACCCGAATATTTGGTGAATAGTAACACAATCTACTTTGTTATAG
AATCTGTCCTCAATGGTCTTACAGTCTAAGAGCATTACATTTAGTTACGGTAGTCTTAAATATG
ATACTAAATGAATTGAATAGCCAGACATCTGTGAAAGTAAAACTGTGATAATACAAAGTCAGT
GTGCTTTTAAGTTTCAATTGCTTAAAGTTATTTGGAAGTAAGGATCACATGAGTTCAGAGCCC
CAGGATTCAAAAATAACTCCGGAATCTTTTATCACTCCCCAAAGGAAAAGCAATAATAAGTAA
GTTACCAGACATTCATGTAATCTCTCTGAAACTGTAAGACTAACAAAGGAAAATTTTCAAAAG
GCAGAAATACTTTATTATTTATTTGCAATAGAAATTATTCAGCCGGGCGCGGTGGCTCATGCC
TGTAATCCCAGCACTTTGGGAGGCCGAGGCGAGTGGATCACCTGAGGTCGGGAGTTCGAG
ACCAGCCTGACCAACATGGAGAAACCCCGTCTCTACTAAAAATACACAATTAGCTGGGCATG
GTGGCACATGCCTGCAAGCCCAGCTACTCAGGAGGCTAAGGCAGGAGCGACAGAGCGAG
ACTCCATCTCAAAAAAAAAAGAAAGAAAGAAAGGAAAAAAAGAAAAGAAAAGAAACAAAGAA
ATTTTTCAGTAAATACATACAACAGCATTGATGGCCTGGAGACATACCACCTTCAGTTGTGAT
CCTGGCAGATGTGATAGATTCTACAAGGTTGGACAGGTTAAATTCTTGCAAGGAAGTCCTTT
CTGGAACACCCAAGAATTACAGAAAGTTTAGCTATCTTTCAGATGAGATTTCATTCTCAGTTT
TACTTGTTCCATGACCCTTCCAAACTACACACGGGATTCTTTTCATTCAGTTGCCTTGGCCA
GCATTTTATCACCAATAGTCCTATTTTATGTAACTGAGTTTGCCTTGCCCTTTACTGCATTTAG
GTAAATGGAATGGTGTTTTTTAGACTTCCGAAACTATTTGTAGACCTTTTCCTTCACAAGTTT
GAAATAGATGTGAGGCATTCATTTTACTCAGACAACATTGAGCCCAATTCACTTGGGAAAATG
CATCTTGATGTGTAATTCATCATCCAGAGCCTGTCCACTATTCTGTTCCTCCCTGAGAGTAAA
TTAGGCACTTTGAAAGGTGCATTAAACTCTTCCTGGCTACTAGGTACCATTTAGAACTGTGG
GGCAATAATTTACTTCATGGTACCTTGGTGACCTCAAAAAGTTTATATTGTTTTGAAAGTTCTT
AATTTTCTCCCACTAAATAATTTATGCATTCAGAGGTTGGGTGTGCTTATGTTTCCAGAGAGA
CAACAGCTATTGAATAGCATAAATAAGATAAAATAGTCATGCTAGTGTAGAAATTAAATGTTCA
CAAATGTGTTATCGAGTCTATCCATAGAATAAAAGTGTAGCTTATAAAACAATTATTGCTTTGG
ATTTCATGTTTTAAATTTACAAGACTGTGTTACATTTTGTTGTAATTAGTAATGCTTCATAGCTA
CAAGGATTACCAGATCCATATCACTGCATTTTTCCATAGCACAAAACACATTTATCCAAAGTAA

AAATAGTGTGCTAAACATACTGAAGTTTCTGACAGACACTTATATTTCTCTTCATTCTTTCATT
CACAGCAGAGTTTCATCCATATTAAATAAATTGGTTAAGTTTTGATACCTCCTACTTTAATCACT
CATCTTATGTACTAAAATGAACCAAATTAAGTTTGAGCCAACAAATAAAAGTGATATGCTTTGA

**Isoform 4**

GTGCAACACCTGAAATTCCTGGGGCGAATCAAACAGCAAATTAAAGCTCAGACAACCTTCA
CCTGCAAACCATGCACAGTATAAGGAACTCATTTTAATGTTTTCTGGCTGAAGTCATAGGGG
ATAAGAAAACAAAAGAATGGCCACGAAGGAATCCTGGACATTGTTACCATTTACCTCCACTG
GATATCTATTTTCTTTCAAAAAGCACAATCAACGTGCCTTTTCCCTGATTTTCTCTGCCAAACA
TATCAGATTGTGGGAGGTTTATGGATACACAGTACTTCACAGGCAACCTGAAAGGTCCCAAT
TTGGGCCCCGAGTCTGCTGGAGAAAAGAGAAAGGCTAAGAAGCAGCCTACCTTACCTGTG
GCTCTGGGTCTGAGGATGGCCAGGAGAACAGAGAGCAAAGCAGAGCCAATGCAAGACCTC
TGGCCCTGGAAGCCGAAGGGTCTCTCACAGGTCAACGCAGGATCTACTCCATCATCATCTT
CACTAGCACACCCAGTTCGATTGAGGAGCCTTGACGTGGGATATTGATGGGATATTGAGAG
AAAAGAGAAATGGTGAATCAAGCACTAATCTCAAAGCTTCTGCACAGAACTACTTACATGTCA
CTTCCTCCCATGACTATTCCTAAATTCAGCCAAAAGGGGACACATAATCTCTTACGGGGGAG
GGCACCACGGGGAGAGAAACCCGAATATTTGGTGAATAGTAACACAATCTACTTTGTTATAG
AATCTGTCCTCAATGGTCTTACAGTCTAAGAGCATTACATTTAGTTACGGTAGTCTTAAATATG
ATACTAAATGAATTGAATAGCCAGACATCTGTGAAAGTAAAACTGTGATAATACAAAGTCAGT
GTGCTTTTAAGTTTCAATTGCTTAAAGTTATTTGGAAGTAAGGATCACATGAGTTCAGAGCCC
CAGGATTCAAAAATAACTCCGGAATCTTTTATCACTCCCCAAAGGAAAAGCAATAATAAGTAA
GTTACCAGACATTCATGTAATCTCTCTGAAACTGTAAGACTAACAAAGGAAAATTTTCAAAAG
GCAGAAATACTTTATTATTTATTTGCAATAGAAATTATTCAGCCGGGCGCGGTGGCTCATGCC
TGTAATCCCAGCACTTTGGGAGGCCGAGGCGAGTGGATCACCTGAGGTCGGGAGTTCGAG
ACCAGCCTGACCAACATGGAGAAACCCCGTCTCTACTAAAAATACACAATTAGCTGGGCATG
GTGGCACATGCCTGCAAGCCCAGCTACTCAGGAGGCTAAGGCAGGAGCGACAGAGCGAG
ACTCCATCTCAAAAAAAAAAGAAAGAAAGAAAGGAAAAAAAGAAAAGAAAAGAAACAAAGAA
A

**Supplementary File 2**

**IVT primers**

| | |
|---|---|
| T7-Northern1235-F | TAATACGACTCACTATAGGGTGTGTCCCCTTTTGGCTGAAT |
| Northern-1235-R | GACATTGTTACCATTTACCTCCACT |

**RACE primers**

| | |
|---|---|
| 5'1235-1 | AATCGAACTGGGTGTGCTAGTGAAG |
| 5'1235-2 | CTCTGCTTTGCTCTCTGTTCTCCTG |
| 5'1235-3 | CAGGTAAGGTAGGCTGCTTCTTAGC |
| 5'1235-4 | GCCTTTCTCTTTTCTCCAGCAGACT |
| 3'1235 | GGAGTAGATCCTGCGTTGACCTGT |

**RT-qPCR primer**

| | |
|---|---|
| LINC01235-F | GTCTCTCACAGGTCAACGCAG |
| LINC01235-R | TGTGTCCCCTTTTGGCTGAAT |
| qMCM3-F | GCGCAGGAAAAACGAGAAGAG |
| qMCM3-R | AATGGAGGCCACAAAATCCTTT |
| qMCM4-F | TGAACCTCTATACATGCAACGAC |
| qMCM4-R | CAGGGTAACGGTCAAAGAAGATT |
| qMCM5-F | AGCATTCGTAGCCTGAAGTCG |
| qMCM5-R | CGGCACTGGATAGAGATGCG |
| qMCM6-F | TCGGGCCTTGAAAACATTCGT |
| qMCM6-R | TGTGTCTGGTAGGCAGGTCTT |
| qCDC45-F | CTTGAAGTTCCCGCCTATGAAG |
| qCDC45-R | GCATGGTTTGCTCCACTATCTC |
| qGINS1-F | ACGAGGATGGACTCAGACAAG |
| qGINS1-R | TGCAGCGTCGATTTCTTAACA |

| | |
|---|---|
| qCDT1-F | AGGACACCATCTCTGAGCTTG |
| qCDT1-R | GCACCTGGTACTTGTAGGGC |
| qCDC6-F | ATGGCACAGTATCTGGAGGAG |
| qCDC6-R | TAAGCTGGACTCACTCTCGGA |
| qRFC5-F | GAAGCAGACGCCATGACTCAG |
| qRFC5-R | GACCGAACCGAAACCTCGT |
| qRFC2-F | GTGAGCAGGCTAGAGGTCTTT |
| qRFC2-R | TGAGTTCCAACATGGCATCTTTG |
| qGINS2-F | CCAATGCCCAGCCCTTACTAC |
| qGINS2-R | CTGCCTTCGGGATGTTGTCT |
| qGINS3-F | ACTTTTATCGGACGTTTTCGCC |
| qGINS3-R | TCTCCATCTCGTCTAGCCTGG |
| qGINS4-F | AGTTGGCCTTTGCCAGAGAG |
| qGINS4-R | GAACTGCCCGAAAGAGGTCC |
| qXRCC5-F | GCACTGACAATCCCCTTTCTG |
| qXRCC5-R | TCAATGTCCTCCAGCAAATCAAA |
| qXRCC6-F | GCTAGAAGACCTGTTGCGGAA |
| qXRCC6-R | TGTTGAGCTTCAGCTTTAACCTG |
| qXRCC1-F | TGTGGTCCTACAGTTGGAGAA |
| qXRCC1-R | AAACATGCGAACGCGGTTG |
| qLIG1-F | GAAGGAGGCATCCAATAGCAG |
| qLIG1-R | ACTCTCGGACACCACTCCATT |
| qLIG3-F | GGGAAGCCATCTAAGATCACG |
| qLIG3-R | CACACAGAACCGTTGCTCAG |
| qPCNA-F | ACACTAAGGGCCGAAGATAACG |
| qPCNA-R | ACAGCATCTCCAATATGGCTGA |
| qGAPDH-F | ACAACTTTGGTATCGTGGAAGG |

| | |
|---|---|
| qGAPDH-R | GCCATCACGCCACAGTTTC |
| qACTB-F | GCCAACCGCGAGAAGATGA |
| qACTB-R | CATCACGATGCCAGTGGTA |
| qNEAT1-F | GCTGGAGTCTTGGGCACGGC |
| qNEAT1-R | TCAACCGAGGCCGCTGTCTC |

ChIP-qPCR Primers

| | |
|---|---|
| qdnSMAD4-F | ACGCAGGTCCTCAACACAGA |
| qdnSMAD4-R | AAAGTATCCAAGGAGCGCGG |
| qdnIL17RA-F | AGTTGGGATTTCACGTCCAGTG |
| qdnIL17RA-R | CCGCAGCAACTGTCGATTTTA |
| qdnRNF43-F | TGCACTTCAACCATACATACTGCT |
| qdnRNF43-R | AGAGAAAGGAAGGGCCAAAACTAC |
| qdnRPS6KB2-F | GCCGTGTTTGATTTGGATTTGGAG |
| qdnRPS6KB2-R | GAGAAGGATCTGGGTCTGCAAAGA |
| qdnERBB2-F | GTTGCCACTCCCAGACTTGTT |
| qdnERBB2-R | GGTTTCTCCGGTCCCAATGGA |
| qdnMYC-F | TACGTTGCGGTCACACCCTT |
| qdnMYC-R | TCCGGGTCGCAGATGAAACT |
| qdnCDC6-F | ATGCGTGGTGTGAAGGAGGT |
| qdnCDC6-R | GCACCCGCCACATTTAGTCT |
| nc-1-F | CAAATACCCCTAACCTCAAGGGAG |
| nc-1-R | TTTTTCTCACCTTGAACGGGCTC |
| nc-2-F | ACTTTAAGCACTCCACCTGATACC |
| nc-2-R | TACATTTCACGTCAAGAGCTCAGT |

**CRISPR gRNAs**

LINC01235-1 GGTCTCTCACAGGTCAACGC

LINC01235-2 TGAAACTGTAAGACTAACAA

ScrambleNC AGGCGACTCGTCCCGCCTTC

**ASOs & siRNA target sequences**

ASO-1 GAGAGAAACCCGAATATTTG

ASO-2 ACCAGATCCATATCACTGCA

ASO-3 AGGAATCCTGGACATTGTTA

ASO-4 GAATAGCCAGACATCTGTGA

ASO-5 TAGAAATTATTCAGCCGGGC

ASO-6 GATTACCAGATCCATATCAC

ASO-7 TCAAGCACTAATCTCAAAGC

ASO-8 ATCACATGAGTTCAGAGCCC

hXRCC5 si-1 AGAGGAAGCCTCTGGAAGTTC

hXRCC5 si-2 CGTGGGCTTTACCATGAGTAA

hXRCC5 si-3 CCTCATATCAAGCATAACTAT

**ChIRP probes**

BioProbe2-1235-1 AGGAATTTCAGGTGTTGCAC

BioProbe2-1235-2 AGTTCCTTATACTGTGCATG

BioProbe2-1235-3 TCTACCAGGATTCCTTCGTG

BioProbe2-1235-4 ATGCATCTTTCTCTCAAAGG

BioProbe2-1235-5 TCAGGGAAAAGGCACGTTGA

BioProbe2-1235-6 CAAATTGGGACCTTTCAGGT

BioProbe2-1235-7 CTTGCATTGGCTCTGCTTTG

BioProbe2-1235-8 TGGGTGTGCTAGTGAAGATG

BioProbe2-1235-9 GACCATTGAGGACAGATTCT

| | |
|---|---|
| BioProbe2-1235-10 | TTCACAGATGTCTGGCTATT |
| BioProbe2-1235-11 | TTATTTTTGAATCCTGGGGC |
| BioProbe2-1235-12 | GTCTTACAGTTTCAGAGAGA |
| BioProbe2-1235-13 | CATCAATGCTGTTGTATGTA |
| BioProbe2-1235-14 | GTGTAGTTTGGAAGGGTCAT |
| BioProbe2-1235-15 | TGCAGTAAAGGGCAAGGCAA |
| BioProbe2-1235-16 | ATGCCTCACATCTATTTCAA |
| BioProbe2-1235-17 | GACAGGCTCTGGATGATGAA |
| BioProbe2-1235-18 | CAATAGCTGTTGTCTCTCTG |
| BioProbe2-1235-19 | GCTACACTTTTATTCTATGG |
| BioProbe2-1235-20 | TCCTTGTAGCTATGAAGCAT |
| BioProbe2-1235-21 | CTTCAGTATGTTTAGCACAC |
| BioProbe2-1235-22 | CACTTTTATTTGTTGGCTCA |
| BioProbe2-LUC-1 | CATCTTCCAGCGGATAGAAT |
| BioProbe2-LUC-2 | CCGAACGGACATTTCGAAGT |
| BioProbe2-LUC-3 | CGCAACTGCAACTCCGATAA |
| BioProbe2-LUC-4 | AGCTTTTTTTGCACGTTCAA |
| BioProbe2-LUC-5 | CTCTGGCACAAAATCGTATT |
| BioProbe2-LUC-6 | TCTCTGGCATGCGAGAATCT |
| BioProbe2-LUC-7 | AGACGACTCGAAATCCACAT |
| BioProbe2-LUC-8 | TTTGTCAATCAGAGTGCTTT |
| BioProbe2-LUC-9 | TAGCTGATGTAGTCTCAGTG |
| BioProbe2-LUC-10 | CAGTTCGCCTCTTTGATTAA |

## Supplementary file 2 - uncropped images

Figure S2c

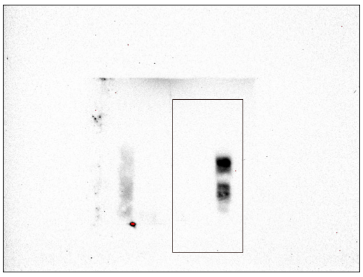

Fig.3c

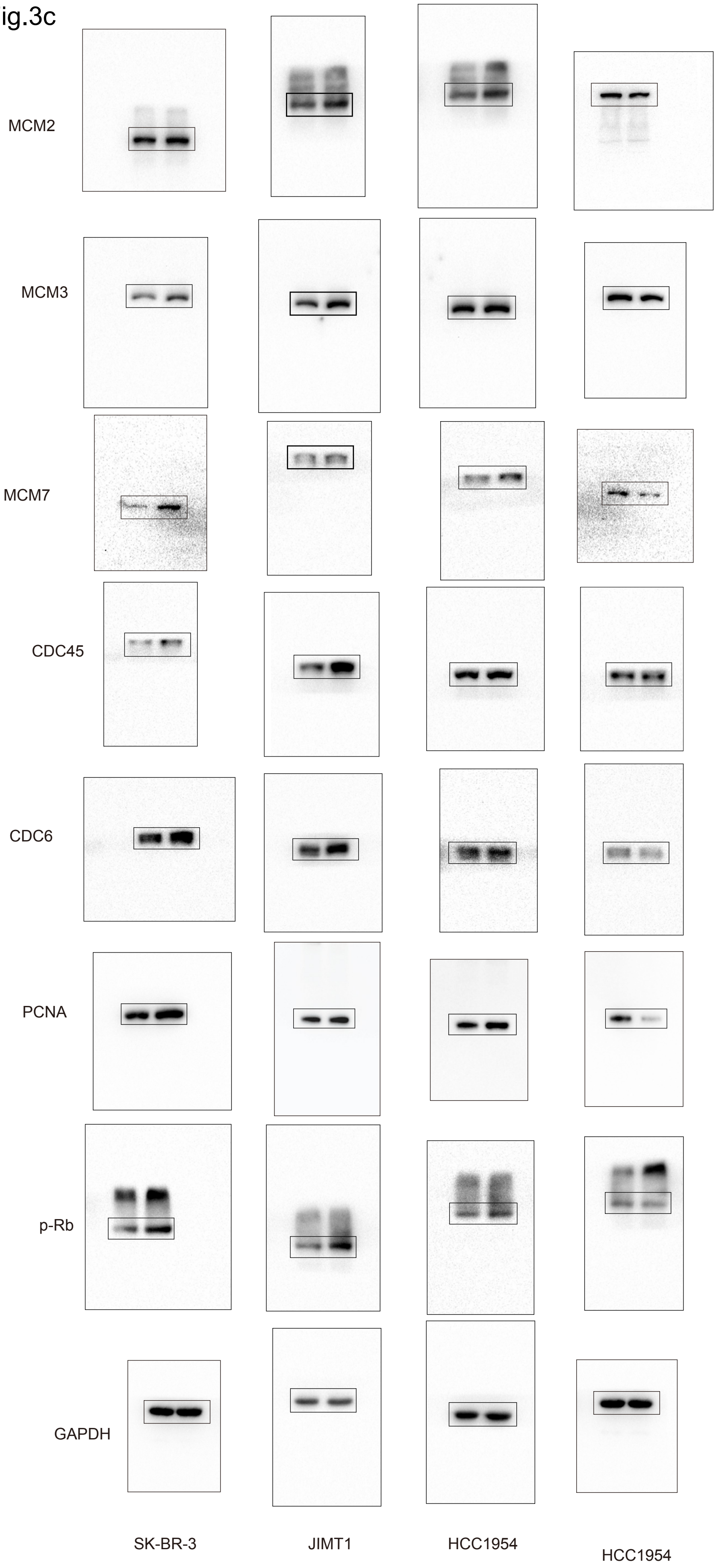

Fig.3i

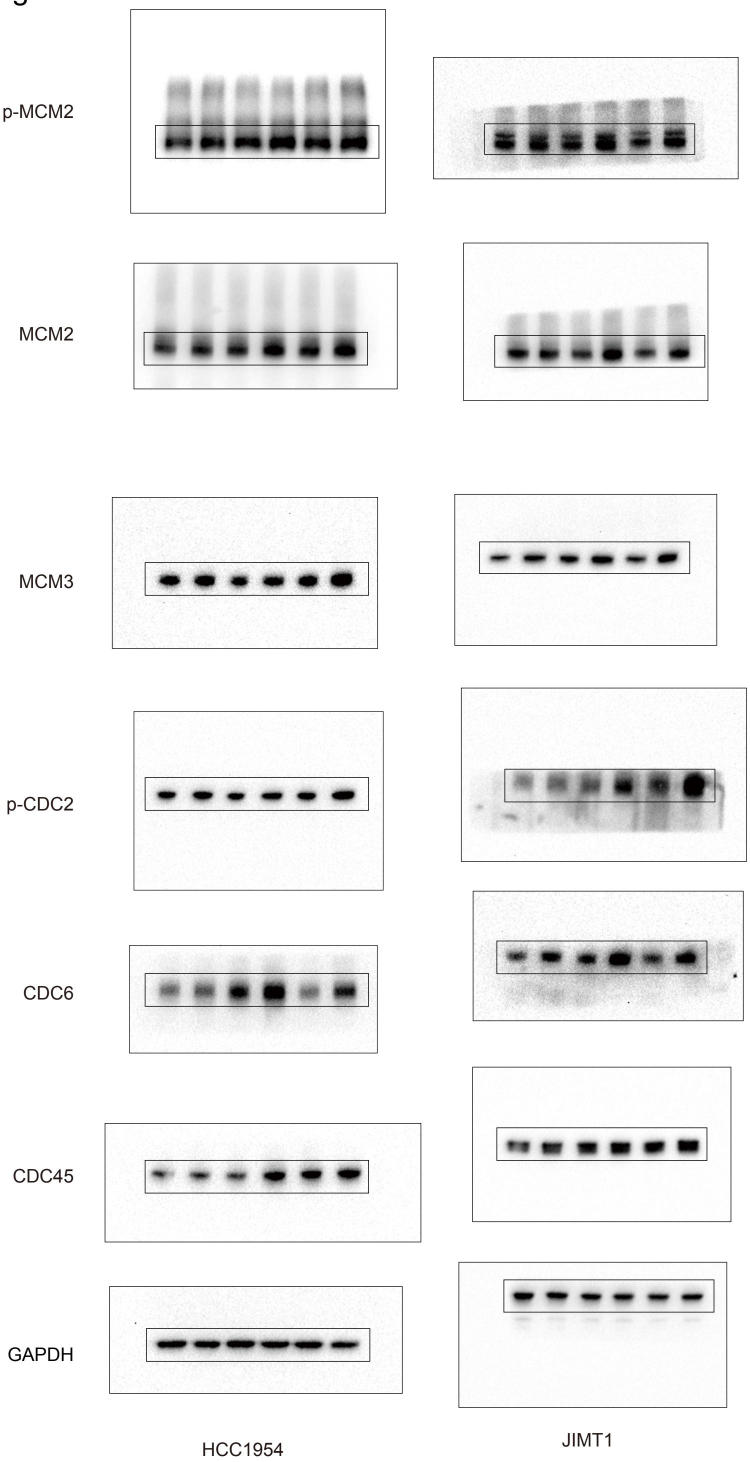
p-MCM2
MCM2
MCM3
p-CDC2
CDC6
CDC45
GAPDH
HCC1954
JIMT1

Fig. 3j

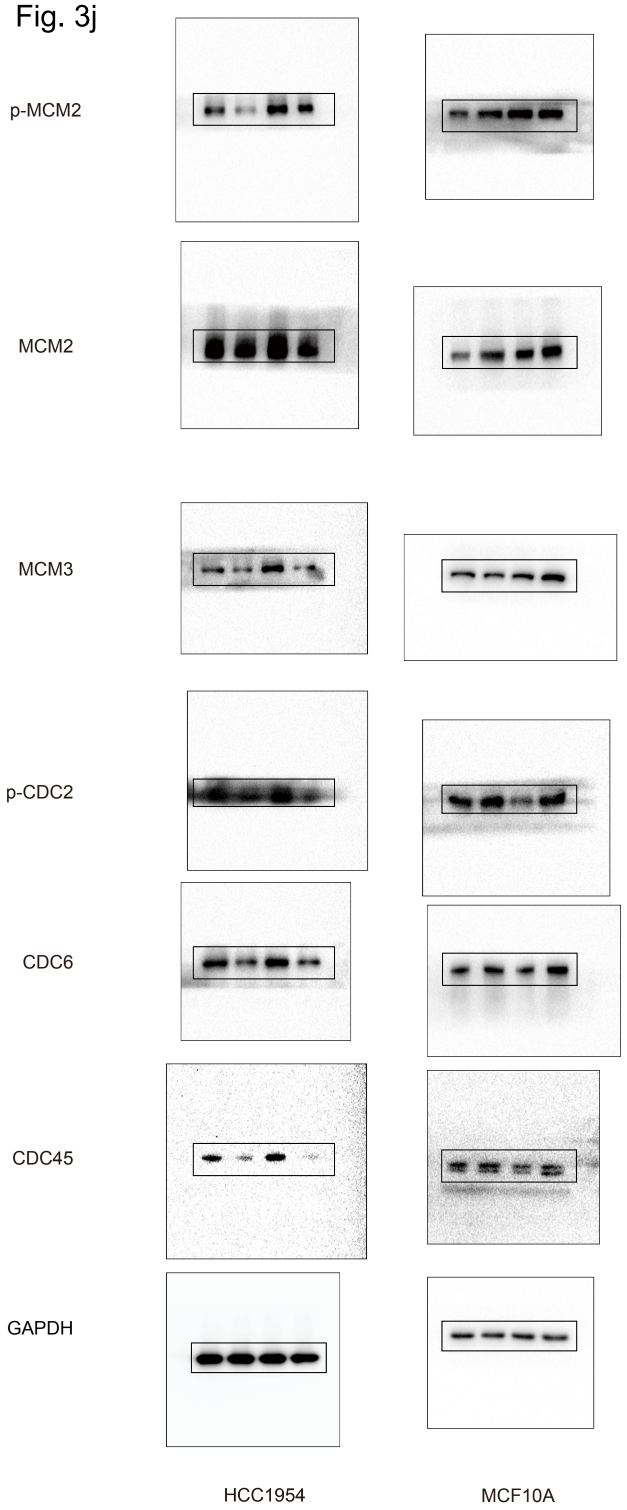
p-MCM2
MCM2
MCM3
p-CDC2
CDC6
CDC45
GAPDH
HCC1954
MCF10A

Figure S3b

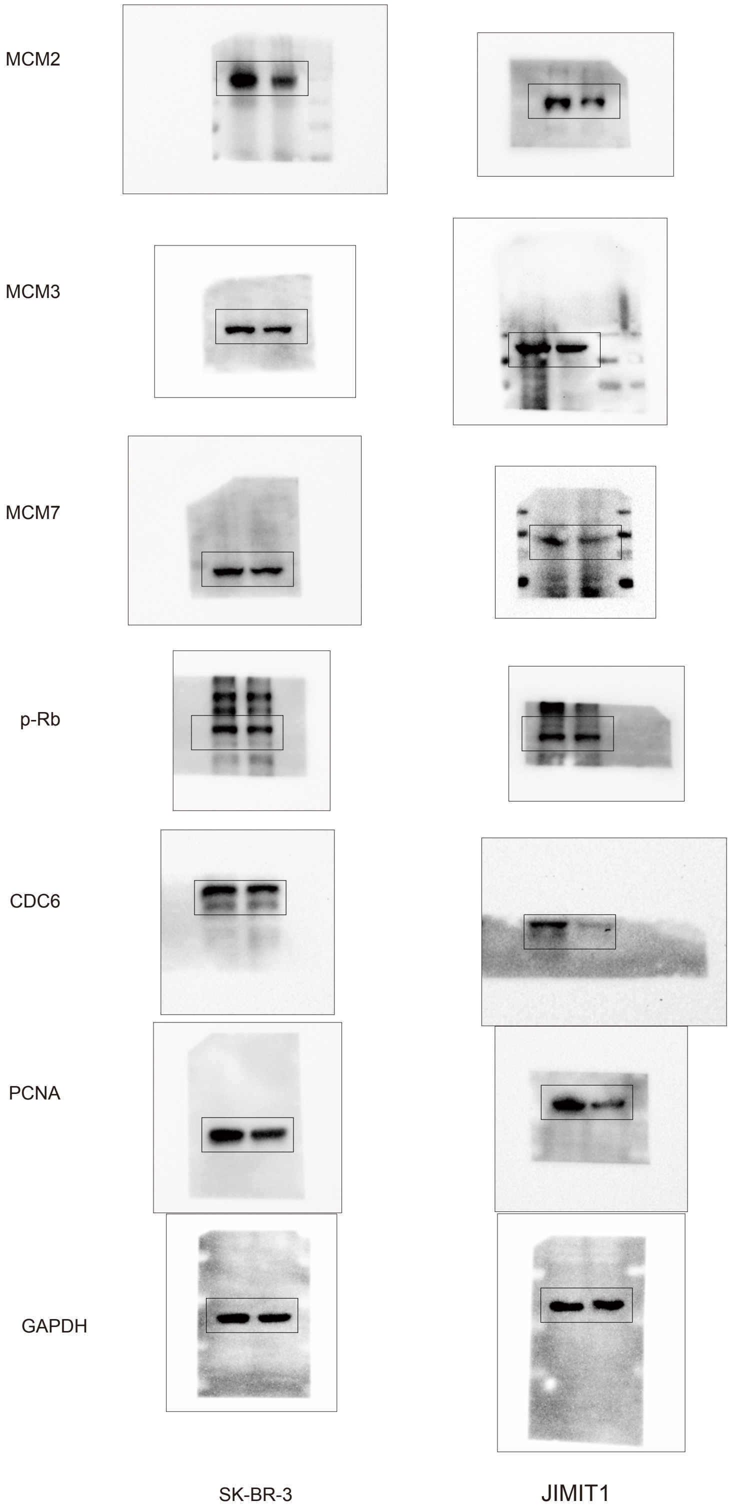


Figure S3g

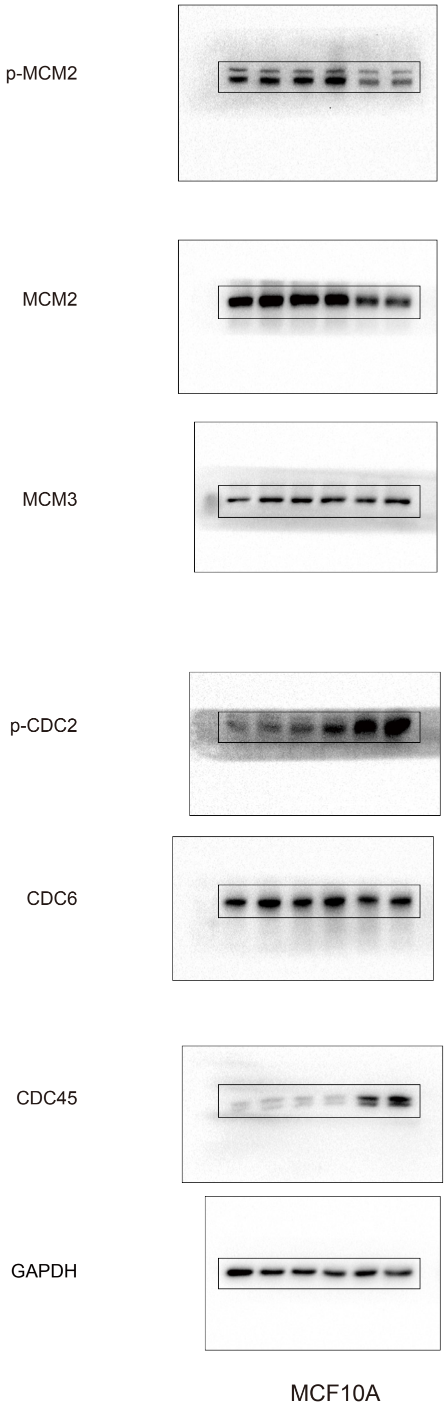

Figure 4i

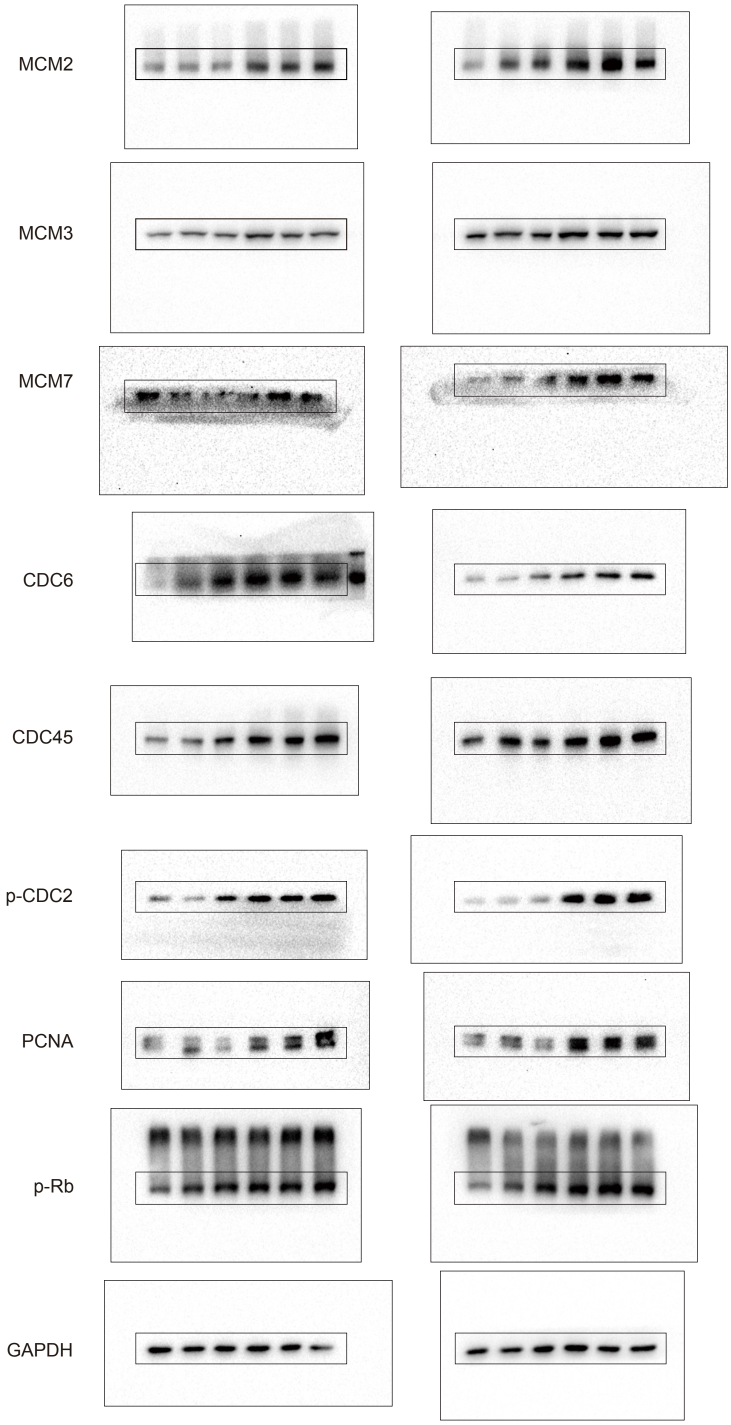


Figure S4a

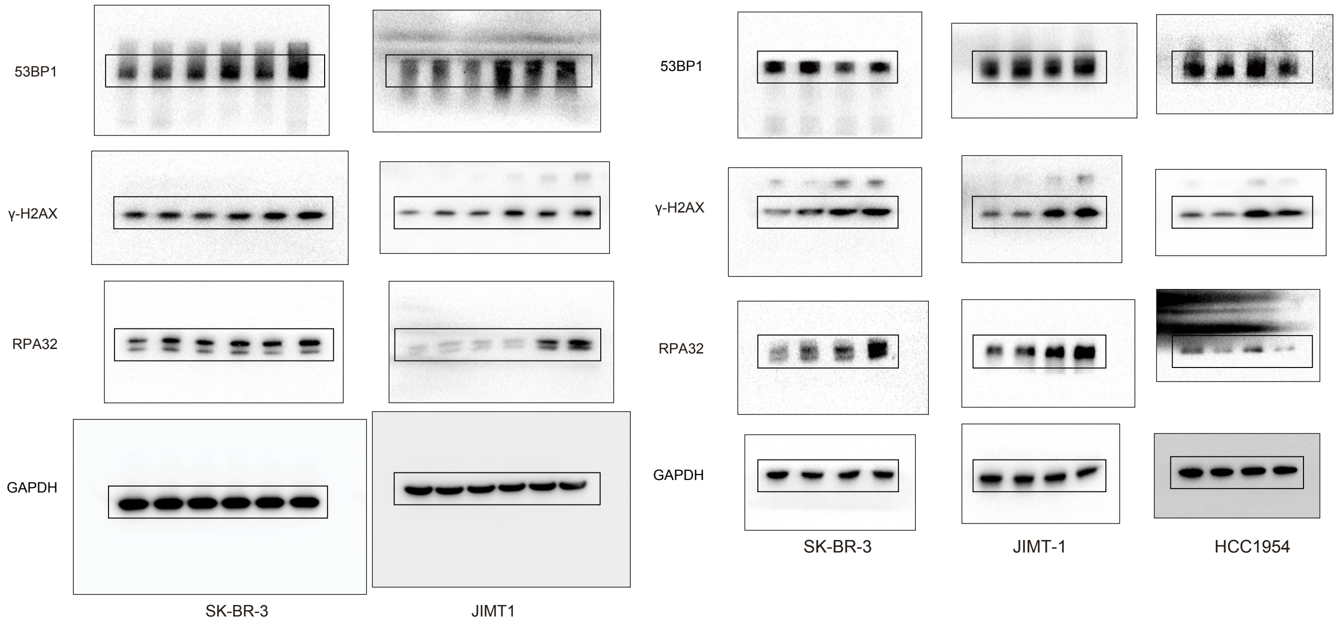

Fig. 6b

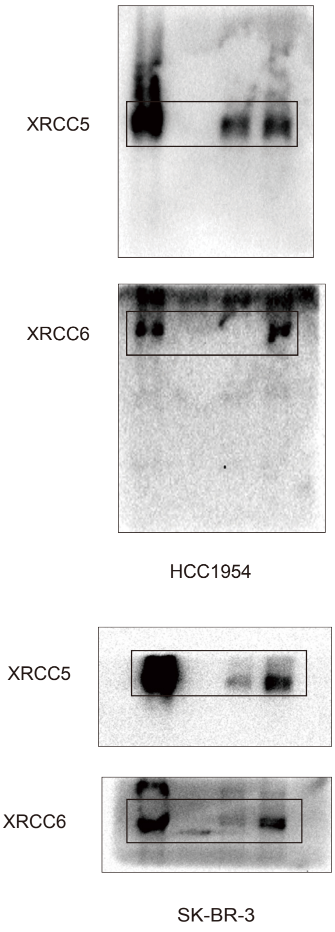


Fig. 6f

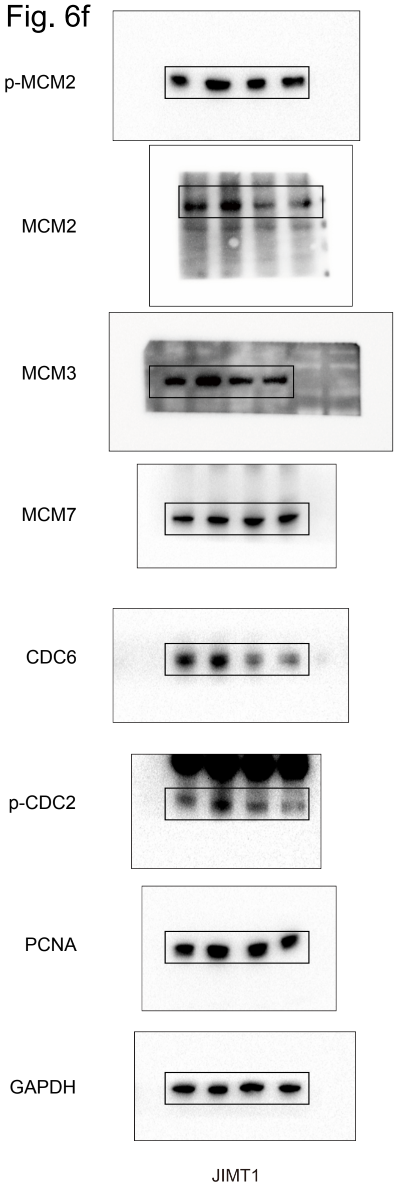


Fig. 6i

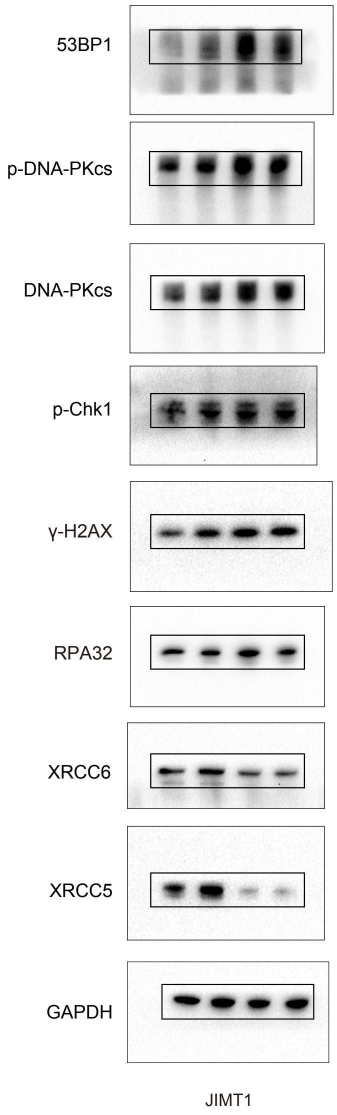


Figure S6c

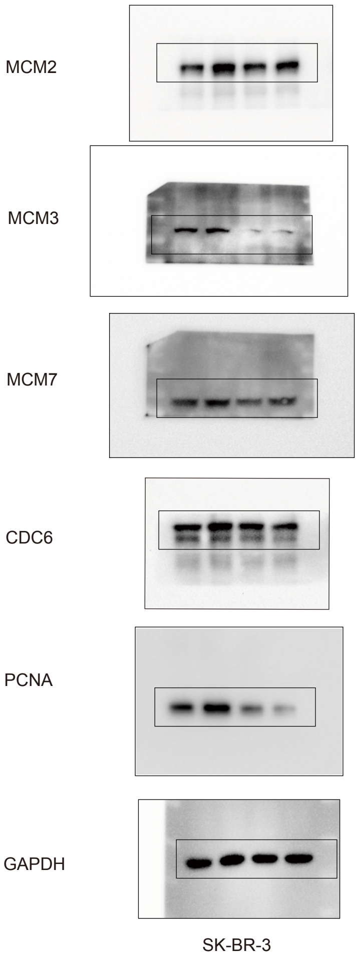


Fig. 7b

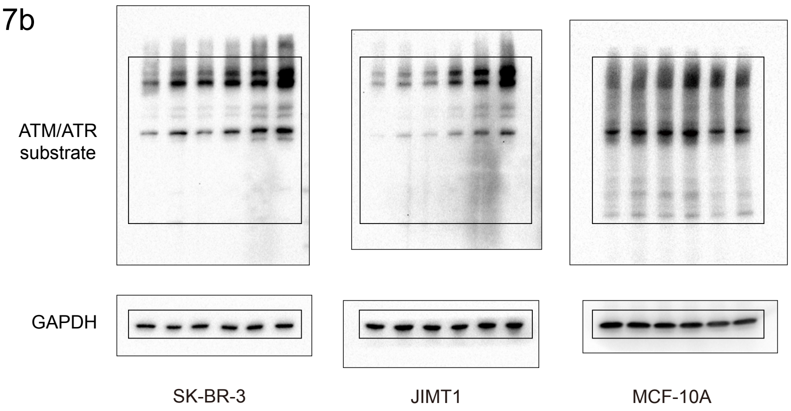

Supplement: Supplementary file 1 — Supporting Information [file ADVS-12-2413527-s001.pdf]
